# Effectiveness, quality and implementation of pain, sedation, delirium, and iatrogenic withdrawal syndrome algorithms in pediatric intensive care: a systematic review and meta-analysis

**DOI:** 10.3389/fped.2023.1204622

**Published:** 2023-06-16

**Authors:** Ibo MacDonald, Véronique de Goumoëns, Mark Marston, Silvia Alvarado, Eva Favre, Alexia Trombert, Maria-Helena Perez, Anne-Sylvie Ramelet

**Affiliations:** ^1^ Institute of Higher Education and Research in Healthcare, University of Lausanne, Lausanne, Switzerland; ^2^ La Source School of Nursing, HES-SO University of Applied Sciences and Arts Western Switzerland, Lausanne, Switzerland; ^3^ Bureau d’Echange des Savoirs pour des praTiques exemplaires de soins (BEST) a JBI Center of Excellence, Lausanne, Switzerland; ^4^ Department Woman-Mother-Child, Lausanne University Hospital, Lausanne, Switzerland; ^5^ Department of Adult Intensive Care, Lausanne University Hospital, Lausanne, Switzerland; ^6^ Medical Library, Lausanne University Hospital and University of Lausanne, Lausanne, Switzerland

**Keywords:** algorithm, delirium, iatrogenic withdrawal syndrome, meta-analysis, pain, pediatric intensive care, sedation, systematic reveiw

## Abstract

**Background:**

Pain, sedation, delirium, and iatrogenic withdrawal syndrome are conditions that often coexist, algorithms can be used to assist healthcare professionals in decision making. However, a comprehensive review is lacking. This systematic review aimed to assess the effectiveness, quality, and implementation of algorithms for the management of pain, sedation, delirium, and iatrogenic withdrawal syndrome in all pediatric intensive care settings.

**Methods:**

A literature search was conducted on November 29, 2022, in PubMed, Embase, CINAHL and Cochrane Library, ProQuest Dissertations & Theses, and Google Scholar to identify algorithms implemented in pediatric intensive care and published since 2005. Three reviewers independently screened the records for inclusion, verified and extracted data. Included studies were assessed for risk of bias using the JBI checklists, and algorithm quality was assessed using the PROFILE tool (higher % = higher quality). Meta-analyses were performed to compare algorithms to usual care on various outcomes (length of stay, duration and cumulative dose of analgesics and sedatives, length of mechanical ventilation, and incidence of withdrawal).

**Results:**

From 6,779 records, 32 studies, including 28 algorithms, were included. The majority of algorithms (68%) focused on sedation in combination with other conditions. Risk of bias was low in 28 studies. The average overall quality score of the algorithm was 54%, with 11 (39%) scoring as high quality. Four algorithms used clinical practice guidelines during development. The use of algorithms was found to be effective in reducing length of stay (intensive care and hospital), length of mechanical ventilation, duration of analgesic and sedative medications, cumulative dose of analgesics and sedatives, and incidence of withdrawal. Implementation strategies included education and distribution of materials (95%). Supportive determinants of algorithm implementation included leadership support and buy-in, staff training, and integration into electronic health records. The fidelity to algorithm varied from 8.2% to 100%.

**Conclusions:**

The review suggests that algorithm-based management of pain, sedation and withdrawal is more effective than usual care in pediatric intensive care settings. There is a need for more rigorous use of evidence in the development of algorithms and the provision of details on the implementation process.

**Systematic Review Registration:**

https://www.crd.york.ac.uk/prospero/display_record.php?ID=CRD42021276053, PROSPERO [CRD42021276053].

## 1. Introduction

To optimize comfort and minimize distress, analgesic and sedative medications are integral to the care of children in pediatric intensive care units (PICUs). However, prolonged intravenous administration of opioids and sedatives can lead to tolerance, delirium, and iatrogenic withdrawal syndrome (IWS) (1–3). Maintaining optimal sedation levels is challenging and depends on drug pharmacokinetics that can be altered by clinical factors. Only 57.6% of children achieve optimal sedation levels (4). Under-recognition of pain, agitation, delirium or IWS can have negative consequences for children, including delayed recovery, increased morbidity and mortality, and increased length of intensive care unit (ICU) stay (5–7). The challenge is that pain, sedation, delirium and IWS are distinct but interrelated conditions with overlapping behavioral indicators (3, 8, 9). These overlaps complicate and convolute the assessment process for healthcare professionals (HCPs), as they may use up to four different measurement instruments, each taking time to complete. Up to 50% of children in PICUs are younger than one year of age or are mechanically ventilated (10, 11); thus, they are unable to self-report. They are at the greatest risk and HCPs report this group as the most challenging to assess (12, 13). Appropriate assessment is a prerequisite for appropriate treatment. To promote best practice, available evidence-based (EB) clinical practice guidelines (CPGs) recommend that validated measurement instruments be used for each condition (3, 14). Although multiple measurement instruments exist for assessing pain and sedation (15), delirium (16) and IWS (17), their uptake in clinical practice has been slow and varies worldwide (18, 19). One suggested strategy for facilitating the use of measurement instruments is to incorporate them into management algorithms (3, 20). An algorithm is a visual representation or flowchart that provides a step-by-step sequence of actions and decision points related to a condition (21–23). This facilitates clinical decision-making and standardizes the process in the local context.

Several studies on algorithm implementation in PICUs has been published in the last decade, predominantly quasi-experimental and focused on sedation algorithms. In studies where patients were managed using a sedation algorithm, mixed results have emerged; positive outcomes included reduced PICU length of stay (LOS), decreased total duration of sedation, decreased doses of sedatives, and decreased prevalence of IWS (24, 25). However, two systematic reviews published in 2014 and 2018 were unable to show the effectiveness of algorithm-based sedation management vs. non-protocolized sedation in pediatric patients due to small sample sizes and a lack of randomized controlled trials (RCTs) (24, 26). Quasi-experimental studies can generate strong causal evidence, particularly when RCTs are not possible (27). Establishing the effectiveness of sedation algorithms is important to informing clinical practice; thus, their use in this evaluation is warranted. Sedation algorithms that integrate pain, delirium, and/or IWS contribute to standardized management of sedation and should be pooled as evidence to measure the effect on children in intensive care. To date, no systematic review has reported the effectiveness of algorithm-based management of these four conditions. One systematic review that pooled the results of all available clinical practice pathways showed reduced in-hospital complications and improved documentation (28).

While effectiveness is an important component of implementation (29), researchers have called for greater generalizability of interventions and implementation processes into real-world practice to reduce research waste through the use of systematic reviews (30, 31). However, the implementation of complex health interventions, such as algorithms, are compounded by multiple factors, including the suboptimal evidence base of the intervention of interest (32), the poor planning of the implementation process without considering the context (33, 34) or determinants (barriers or facilitators) (35), and the implementation strategies used for intervention implementation (36, 37). To bridge this gap, systematic reviews of effectiveness can also identify elements of the implementation process, such as determinants and common implementation strategies. Organizations trying to adopt an algorithm-based management intervention can use these results and save time by not reassessing known determinants. None of the reviews on sedation algorithms have evaluated the structure and the content of algorithms or the implementation processes that contributed to the success or failure of these complex health interventions.

Effectiveness is not the sole indicator of quality of care. Donabedian's concepts of “structure-process-outcome” are universally accepted as a framework for quality assessment (38). Structure refers to the attributes of the setting (38); in this review, these are the attributes of the algorithm. Process refers to the components of care delivery, and outcome refers to the health status of patients (38). These three concepts are important for understanding complex health intervention implementation. Using Donabedian's concepts, in order of research priorities, the three objectives of this systematic review for evaluating algorithms for managing pain, sedation, delirium and IWS, are as follows:

1) To evaluate the effectiveness of the algorithm for pediatric intensive care patient outcomes (outcome).
2) To evaluate the quality of the content and the development of algorithm attributes (structure).
3) To describe the implementation of the algorithms, including strategies of implementation, the determinants (barriers and facilitators), the fidelity to the algorithm and/or its components, and users' satisfaction (process).

## 2. Methods

The Cochrane Guidelines for Systematic Reviews handbook guided this review (39). The Preferred Reporting Items for Systematic Reviews and Meta-Analyses (PRISMA) (40) and the extension for literature searches (PRISMA-S) (41) were used for reporting. (Completed PRISMA and PRISMA-S checklists available in Supplementary File S1, Tables S1, S2) The review protocol was registered in the International Prospective Register of Systematic Reviews (PROSPERO) CRD42021276053.

### 2.1. Eligibility criteria (PICO)

All studies with a before and after implementation design, including RCTs, quasi-experimental and cohort studies with prospective or retrospective controls. The PICO criteria: population (P) of interest: premature infants and children up to 18 years of age admitted to any pediatric intensive care setting (pediatric and neonatal units were included because the most at-risk are pre-verbal). Intervention (I): studies using an algorithm for at least one of the four conditions with an embedded measurement instrument. Compared (C) to usual/baseline care. Each study had to contain at least one patient outcome (O) of interest: LOS in the ICU or hospital, length of mechanical ventilation (MV), duration of analgesics and sedatives, cumulative dose of analgesics and sedatives, incidence of IWS and delirium, adequate pain and sedation management, scores, length of medication weaning, and methadone use. Studies published after 2005, as this aligns with the publication year of the first CPG on analgesia and sedation in pediatric intensive care (42). Language was restricted to English and French due to review team's knowledge.

### 2.2. Information sources and search strategies

A three-step approach was used to retrieve studies meeting the eligibility criteria:

1) Database searches in PubMed, Embase.com, CINAHL with Full Text (EBSCO), and Cochrane Library (Wiley, Cochrane Database of Systematic Reviews and Cochrane Central Register of Controlled Trials), ProQuest Dissertations and Thesis Global (ProQuest)
2) Complementary searches in Google Scholar. As recommended the first 300 entries (30 pages) were manually assessed (43).
3) Manual citation searches were conducted for all included studies, using the reference list and Web of Science and Google Scholar to identify additional relevant studies on development, implementation, or adaptation of the original algorithm.

A biomedical information specialist (AT) assisted in developing the search strategy. An advanced search strategy was developed for PubMed using Medical Subject Headings (MeSH) and free-terms describing: (1) pain, sedation, delirium or withdrawal, (2) pediatric intensive care, and (3) algorithm, clinical pathway, or protocol. The strategy was then adapted for each informational source, using the appropriate index terms and syntax. No published search filters or hedges were used. Contrary to what was stated in the protocol, no language limit was applied, and records published before 2005 were excluded. In Embase.com, conference abstracts and conference reviews published before 2016 were excluded. All search strategies were peer-reviewed by another librarian using the PRESS checklist (44). The first search strategy was completed on September 29, 2020, updated on December 7, 2021 and November 29, 2022. The full search strategies are available in Supplementary Table S3.

### 2.3. Study selection

The search records were uploaded to Endnote 20 reference manager (Clarivate Analytics, USA), to remove duplicates (AT). The remaining records were uploaded to Rayyan (Qatar Computing Research Institute, Doha, Qatar) for the screening process (45).

Three reviewers (IMD, VdG, and MM) independently screened titles and abstracts for inclusion. Full-text publications meeting inclusion criteria were assessed, and the reasons for exclusion were recorded. Disagreements were resolved through discussion and consensus. One reviewer (IMD) conducted the complementary search on Google Scholar and manual citation searches and identified full-texts, and two reviewers independently assessed inclusion criteria (IMD and MM).

### 2.4. Data extraction, quality assessment and analysis

The review team designed, and pilot tested the data extraction tables; one change was made, the addition of one category to the Effective Practice and Organisation of Care (EPOC) implementation strategies (see section 2.5.2 for details).

Two quality appraisal strategies were used to assess the quality of each study and the quality of each algorithm.

#### 2.4.1. Assessment of the methodological quality of studies

To appraise the methodological quality of each study, three JBI quality appraisal tools were used according to the study design: (1) RCTs (13 items), (2) quasi-experimental studies (9 items), and (3) cohort studies (11 items) (46). Each item was answered in one of the four following ways: yes, no, unclear, or not applicable. Quasi-experimental designs were defined as those with exogenous explanatory variables (treatment or exposure) that the investigator does not control (27). This includes before- and after-design studies without randomization, including quality improvement. No studies were excluded based on quality. Two independent reviewers (IMD and VdG) appraised all the studies. Disagreements were resolved through discussion and consensus, and no additional reviewer was required.

#### 2.4.2. Assessment of the methodological quality of algorithms

No tool exists for the appraisal of the methodological quality of algorithms. We modified the Appraisal of Guidelines for Research and Evaluation (AGREE) II instrument (47) to create the aPpRaisal OF algorIthm quaLity instrumEnt (PROFILE). Modifications included: a) replacing the word CPG with the word algorithm; b) eliminating domain 6: *editorial independence* as this is not applicable for locally developed algorithms; and c) adding eight items related to content and development based on a literature review of clinical pathway appraisal tools (48–51). This review was done because Govender identified this as missing from the AGREE II instrument when using it to appraise algorithms (52). The PROFILE contains 24 items across five domains: (1) *scope and purpose*; (2) *stakeholder involvement*; (3) *rigor of development*; (4) *content and process*; and (5) *implementation*. The domains are further categorized across three processes: (a) development (domains 1–3), (b) content (domain 4), and (c) implementation (domain 5). Each item was scored as either “1 = yes” or “0 = no”. No algorithm was excluded based on quality. The PROFILE has three types of scores: (1) an overall quality score that uses all items; (2) domain scores, five in total, one for each domain; and (3) process scores, three in total. It was pre-tested using one algorithm by all three reviewers (IMD, EF and MM), following which a consensus meeting was used to finalize the items and create a user manual with details for each item (as with the AGREE II instrument). An overview of the PROFILE is available in Supplementary Table S4. One appraiser (IMD) independently evaluated each algorithm, and four reviewers (EF, MM, SA and A-SR) cross-checked all data. Discrepancies were resolved through consensus discussion. The inter-rater reliability was calculated using kappa statistics (53). The scores were represented as a percentage by totaling the number of each present item (1 = yes) and dividing by the total number of items.

### 2.5. Data extraction

The information extracted from each study and how it was analyzed to meet the three objectives are described below. Descriptive information on the study characteristics and details of each algorithm was extracted and summarized in two tables.

#### 2.5.1. Objective 1: to evaluate the effectiveness of the algorithm for pediatric intensive care patient outcomes

Meta-analyses were conducted using STATA version 17 software (54). Random-effects (Hedges' g) models using the Sidik-Jonkman method were used to measure effectiveness of algorithms across continuous outcomes of interest using the standardized mean difference (SMD) and its 95% confidence interval (CI) (55). Random-effects models using the Sidik-Jonkman method was used for dichotomous outcomes of interest using pooled odds ratios (OR) and 95% CI (55).

When the median and interquartile range (IQR) were reported, they were transformed using Wan's method and Excel tool (56).

The *I*^2^ test was used to assess statistical heterogeneity, which was considered low if <40%, moderate 30%–60%, substantial 50%–90% and considerable 75%–100% (57). When heterogeneity was ≥40%, a sensitivity analysis of influencers was performed by removing one study at a time to assess the impact of each study on the overall effect size (57). To determine other sources of heterogeneity, sensitivity analyses were performed by stratifying studies based on the type of setting (PICU or NICU) when two or more studies were available. Risk of bias was not assessed due to the limited number of moderate quality studies. Additionally, study design was not assessed as pooling bodies of evidence has a mainly concordant direction of effect (58).

A subgroup analysis to estimate the treatment effect was performed on the type of algorithm, as recommended in a systematic review of clinical pathways (28). Type of algorithm was determined by the embedded measurement instrument(s) and medications used.

When more than one time point was measured post-implementation, the first point was used because fidelity to implementation was considered the highest. When studies on IWS/weaning used two different algorithms based on different medications, both groups were included in the meta-analysis.

For each outcome of interest a forest plot displaying the meta-analysis was created, additionally subgroup and sensitivity analyses figures were created.

To assess publication bias, funnel plots were generated, and the Egger's test was used to indicate the likelihood of publication bias.

When a meta-analysis could not be performed, the results were presented narratively.

To assess the certainty of the findings, a summary of findings (SoF) table was created using the Grading of Recommendations, Assessment, Development and Evaluation (GRADE) approach (59). The GRADE approach assesses all studies together for each outcome of interest and rates the level of uncertainty for the risk of bias, indirectness, consistency, imprecision, and publication bias (57). The main outcomes included in the SoF table were: (1) LOS intensive care (2) length of MV; (3) duration of analgesics; (4) duration of sedatives; (5) cumulative dose analgesics; (6) cumulative dose sedatives; and (7) incidents of IWS, as seven is the maximum suggestion (39). The selection of these main outcomes was based on the literature and its relevance to the clinical setting (60).

All corresponding authors were sent a personalized email to clarify unclear risk of bias items or missing details of algorithm content, development, and implementation. Six authors provided additional information included in the analysis (61–66).

#### 2.5.2. Objective 2: to evaluate the quality of the content and the development of algorithm attributes

The three PROFILE scores (as described in section 2.4.2) were categorized based on a three-step quality threshold as determined in a systematic review of AGREE II instrument usage (67). A score was classified as “high” if >60%, “medium” if between 59% and 30%, and “low” if <30% (67). The results are presented as a heat map.

#### 2.5.3. Objective 3: to describe the implementation of the algorithms, including strategies of implementation, the determinants, the fidelity to the algorithm and/or its components, and users' satisfaction

To analyze the process of establishing the algorithm in practice, the strategies in each study were categorized according to the EPOC subcategories of interventions targeted at healthcare workers (36). One item called “case-based and scenario evaluation” was added because this could not be categorized in the existing EPOC taxonomy. Results are presented in tabular format.

To analyze the determinants (barriers and facilitators) of algorithm implementation, narrative descriptions were extracted. and categorized as intervention, professional or organizational using Lau's framework (68). Each determinant was further categorized as a barrier or facilitator, and the level of implementation was categorized (pre-implementation, implementation, or post-implementation). The method used to obtain the determinant (measured vs. mentioned but not empirically verified) was recorded. Results are presented in tabular format.

To analyze algorithm fidelity, rates were extracted, reported as percentages and ranges, and presented in a table. Fidelity is defined as whether an intervention has been implemented as intended with two subcomponents: adherence and dose (69). Adherence refers to whether the intervention is delivered as intended, and dose refers to the number of intervention components delivered (69).

Staff and family satisfaction was reported in a table.

## 3. Results

**Figure 1:**
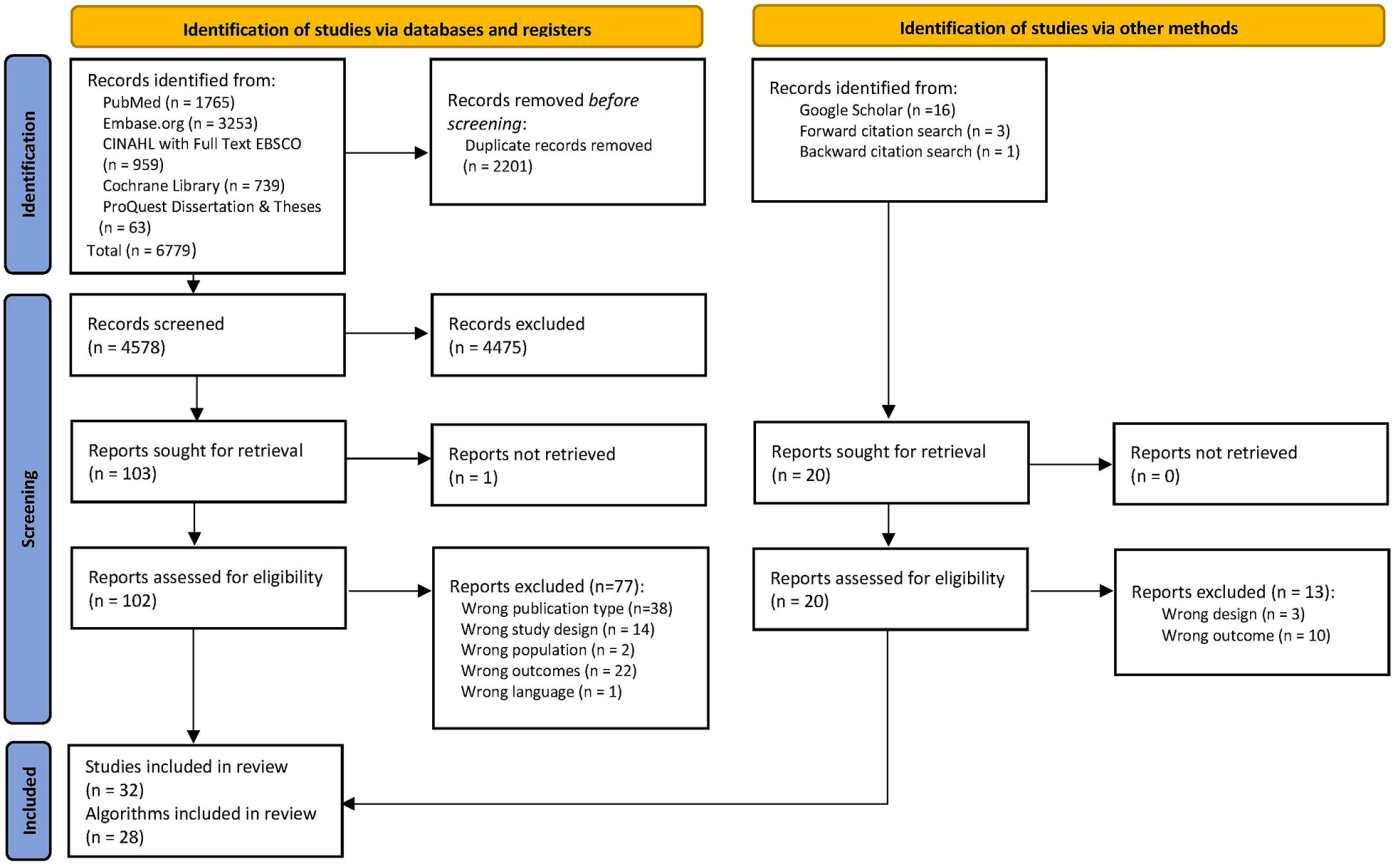
The PRISMA flow diagram summarizes the number of studies excluded in each phase of the selection process (40).

The database search yielded 6779 records and the complementary search (Google Scholar and citation screening) added 20 records. After removing duplicates, 123 full-text studies were screened, and 91 were excluded (Supplementary Table S5). Thirty-two studies met the inclusion criteria (61–66, 70–95), including 28 unique algorithms (Figure 1: PRISMA flow diagram) (40). There was high inter-rater agreement across all extraction tables (*K *= 0.92–0.97).

### 3.1. Study and algorithm characteristics

#### 3.1.1. Study characteristics

The characteristics of the 32 included studies are summarized in Supplementary Table S6, and the details of the algorithms are provided in Supplementary Table S7. Twenty-three studies used a quasi-experimental design (73%) (61, 63, 64, 66, 70–74, 76–83, 85–88, 93, 95), seven were cohort studies (21%) (65, 75, 84, 90–92, 94) and two were RCTs (6%) (62, 89). The studies were conducted across 11 countries: 15 in the United States (47%) (62, 64, 65, 70–72, 74, 75, 86–88, 90–93), 11 in six European countries (34.3%) (61, 63, 66, 73, 76–80, 94, 95), three in Australia (9.4%) (83–85), and three in three Asian countries (9.4%) (81, 82, 89). All but two studies (62, 83) were single-centers (94%) (61, 63–66, 70–82, 84–95). Two thirds of the studies were published in the last five years (66%) (63–66, 71–74, 77, 79–81, 85–93).

The sample comprised 9,289 children (55% in the algorithm/intervention group). Seventy-five percent of the studies (*n* = 24) had a sample with a mean or median age of less than 4 years (61–63, 65, 66, 70–75, 77, 78, 80, 82–85, 87–89, 91, 92, 94, 95). The majority of the settings were PICUs (72%) (61–66, 70, 72, 73, 75–77, 79–83, 85, 89–93), 16% were neonatal ICU (86–88, 94, 95), and 22% were pediatric cardiac ICUs (71, 74, 78, 84, 85).

#### 3.1.2. Algorithm characteristics

**Figure 2:**
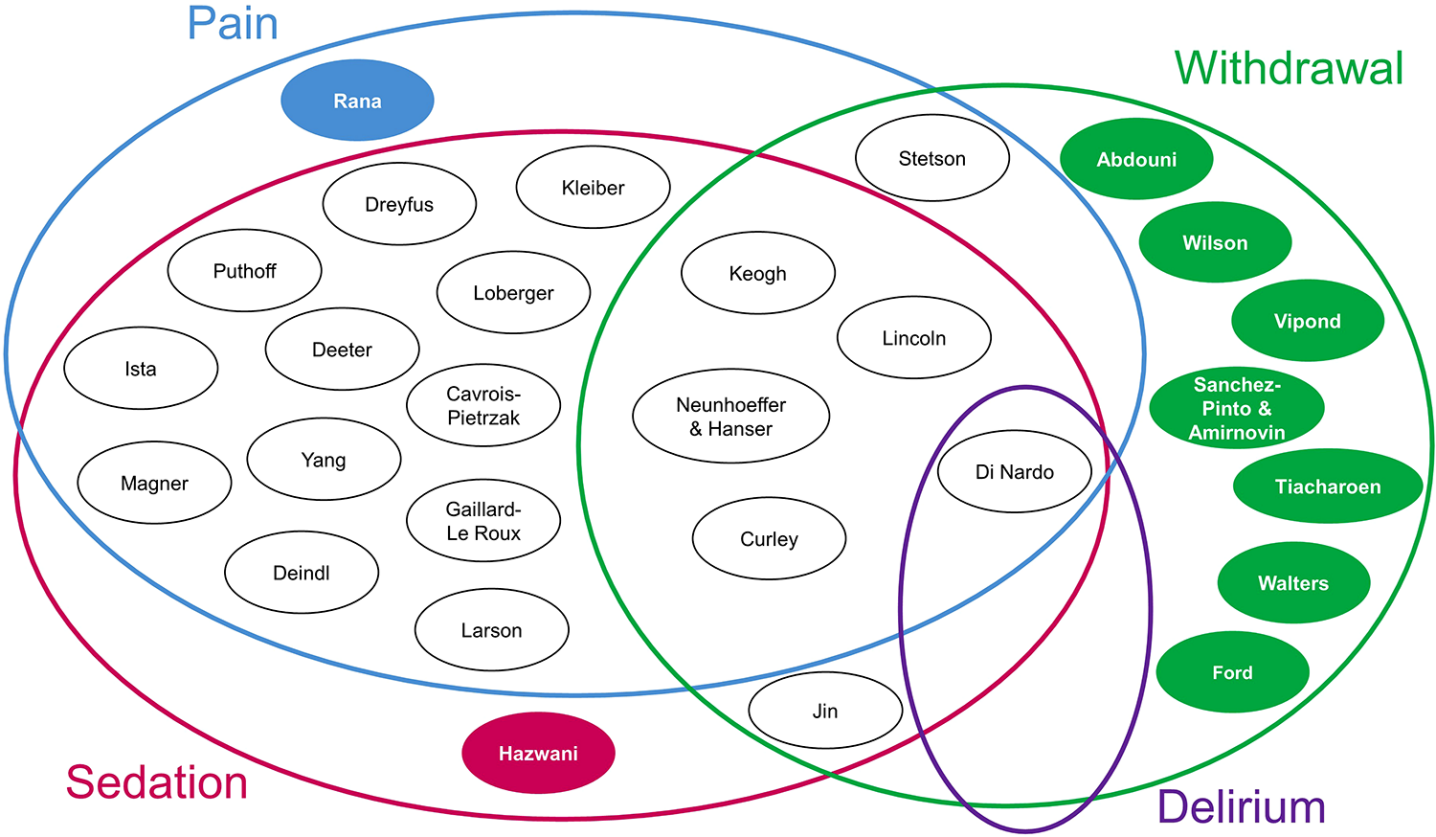
Each circle represents an algorithm, with the first author(s) of each study listed inside the circle. The solid-colored circles represent algorithms that focus on one condition, while the white circles represent algorithms that have overlapping conditions, as presented by the Venn diagram.

The distribution of the 28 algorithms by type of condition represented is shown in Figure 2. Nine (32%) of the 28 algorithms focused on one condition alone (65, 70–72, 81, 87, 89–92), one on pain (87), one on sedation (81), and seven on IWS/weaning (65, 70–72, 89–92). Of the remaining 19 algorithms (68%), all but one (88) combined sedation with at least one other condition, which were distributed as follows: 12 “*pain-sedation*” algorithms (61, 63, 64, 73, 75–77, 84–86, 93–95), four “*pain-sedation-IWS*” algorithms (62, 74, 78–80, 83), one “*pain-sedation-delirium-IWS*” algorithm (66), and one “*sedation-IWS*” algorithm (82). The other combination was “*pain-IWS*” (88).

Nurses were responsible for managing the algorithm (88.9%) (61–66, 70–81, 83–86, 88, 89, 91–93, 95), one algorithm was managed by a pharmacist (3.7%) (82), another by a pharmacist, a critical care physician, and a nurse (3.7%) (90), and one by a pharmacist and nurse (3.7%) (65). One study did not report the HCP responsible for management (87).

Fifteen studies included information on the process and documentation of algorithms. Of these, ten (67%) integrated the measurement instruments into the electronic health record (61, 65, 66, 74, 81, 86, 88, 92–95), two (13%) included a portion as an order set in the electronic health record (70, 85), and three were paper-based at the bedside (63, 64, 71).

The primary analgesic agent used by algorithms was morphine (77%) (61–63, 65, 73–75, 77–80, 83–89, 91, 92, 94, 95) and the primary sedative agent was midazolam (77%) (61–63, 71, 73, 74, 76, 78–80, 82, 83, 85, 86, 89, 92, 94, 95).

The measurement instruments used by algorithm, condition, and measurement frequency are summarized in Supplementary Table S8*.* Of the 14 algorithms that included IWS (62, 65, 66, 70–72, 74, 78–80, 82, 83, 88–92), the most commonly used measurement instrument was the WAT-1 (69%) (62, 65, 66, 71, 74, 89–92), and the monitoring frequency varied from 4 to 12 h (62, 65, 66, 71, 89–92). Nineteen algorithms included pain measurement instruments (61–64, 66, 73–80, 83–88, 93–95), of which seven (37%) assessed pain without using a combined pain and sedation measurement instrument (62, 64, 74, 83, 87, 88, 93), the most commonly used was the FLACC (57%) (62, 64, 74, 93), and was monitored every 4 h (62, 74, 83, 93). Nineteen algorithms included sedation measurement instruments (61–64, 66, 73–86, 93–95), eight studies assessed sedation alone (62, 64, 74, 75, 81–83, 93), and the remaining 11 used a combined measurement instrument (one which combines the assessment of pain and sedation) (61, 63, 66, 73, 76–80, 84–86, 94, 95). When only sedation was assessed, the most often used measurement instrument was the SBS (62, 64, 74, 83, 93), and the most often used monitoring frequency was every 4 h (75%). One algorithm used the COMFORT-B but assessed only sedation (81), another used the COMFORT and assessed only sedation (82), and another used a non-validated measurement instrument, the Seattle PICU Comfort Tool (75). The 11 algorithms that used a combined pain and sedation instrument, 45% used the COMFORT-B (73, 76, 77, 84, 85), 27% used the COMFORT-B + NISS (30%) (61, 66, 78, 80), one used the COMFORT-B + NRS (9%) (63), and 18% used the NPASS (86, 95). The monitoring timeframe varied between 3 and 8 h. One algorithm assessed delirium using the CAPD (66).

### 3.2. Study quality

An overview of the JBI checklist for each study is presented in Supplementary Tables S9–S11. All studies were of high to moderate quality. RCTs were moderate quality, scoring 8 out of 13 (62, 89). All quasi-experimental studies were high quality (61, 63, 64, 66, 70–74, 76–80, 82, 83, 85–88, 93, 95), except one moderate quality, scoring 5 out of 8 (81). Six of the cohort studies were high quality, with scores ranging from 6 to 7 out of 8 (65, 75, 84, 90–92), and one was moderate quality, with a score of 5 out of 8 (94).

### 3.3. Effectiveness

Twenty-six studies were included in meta-analyses on algorithm effectiveness compared to usual care for the outcomes of interest (62–66, 70–80, 82, 87–95). No studies reported on incidents of delirium.

#### 3.3.1. Intensive care unit length of stay

**Figure 3:**
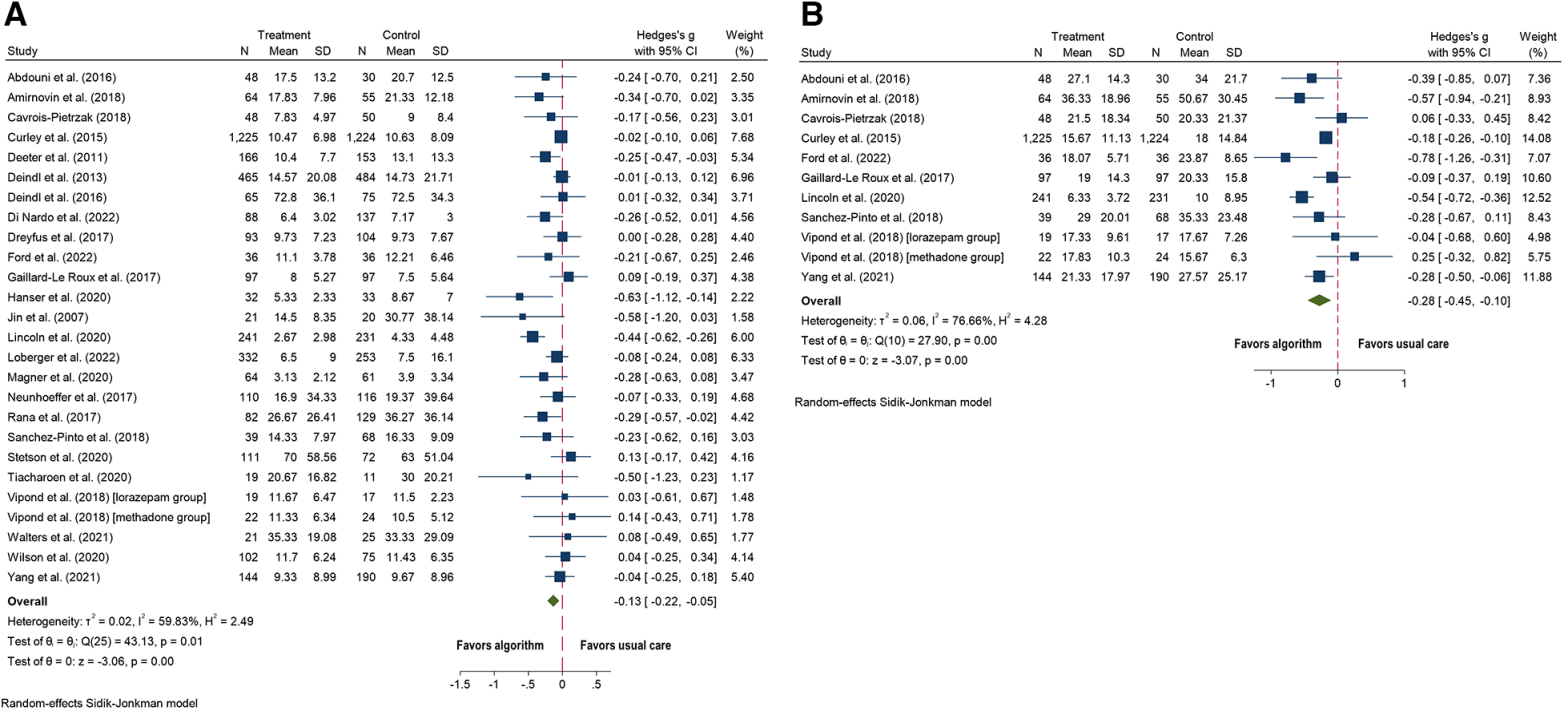
Forest plots for algorithm versus usual care for the outcomes of: (**A**) length of stay in intensive care (days) and (**B**) length of stay in hospital (days).

Twenty-five of the 29 studies with data on ICU LOS were included, with Vipond et al. having two different medication groups (62–66, 70–80, 82, 87–95). The algorithm group showed a small decrease in ICU LOS (SMD = −0.13; 95% CI = −0.22 to −0.05; *p* = 0.01; Figure 3A) compared to the usual care group. Substantial heterogeneity was observed (*I*^2^ = 60%).

Four studies were not included in the meta-analysis because of missing data, three of them showed no significant differences between the two groups (78, 83, 84), while Larson et al. found a significant increase in PICU LOS, but this outcome was measured in hours instead of days (85).

#### 3.3.2. Hospital length of stay

All ten studies with outcome data on hospital LOS were included, and the algorithm group showed a small decrease in hospital LOS (SMD = −0.28; 95% CI = −0.45 to −0.10; *p* = 0.001; Figure 3B) compared to the usual care group (62, 65, 70–74, 77, 90, 93). Substantial heterogeneity was observed (*I*^2^ = 77%).

#### 3.3.3. Length of mechanical ventilation

**Figure 4:**
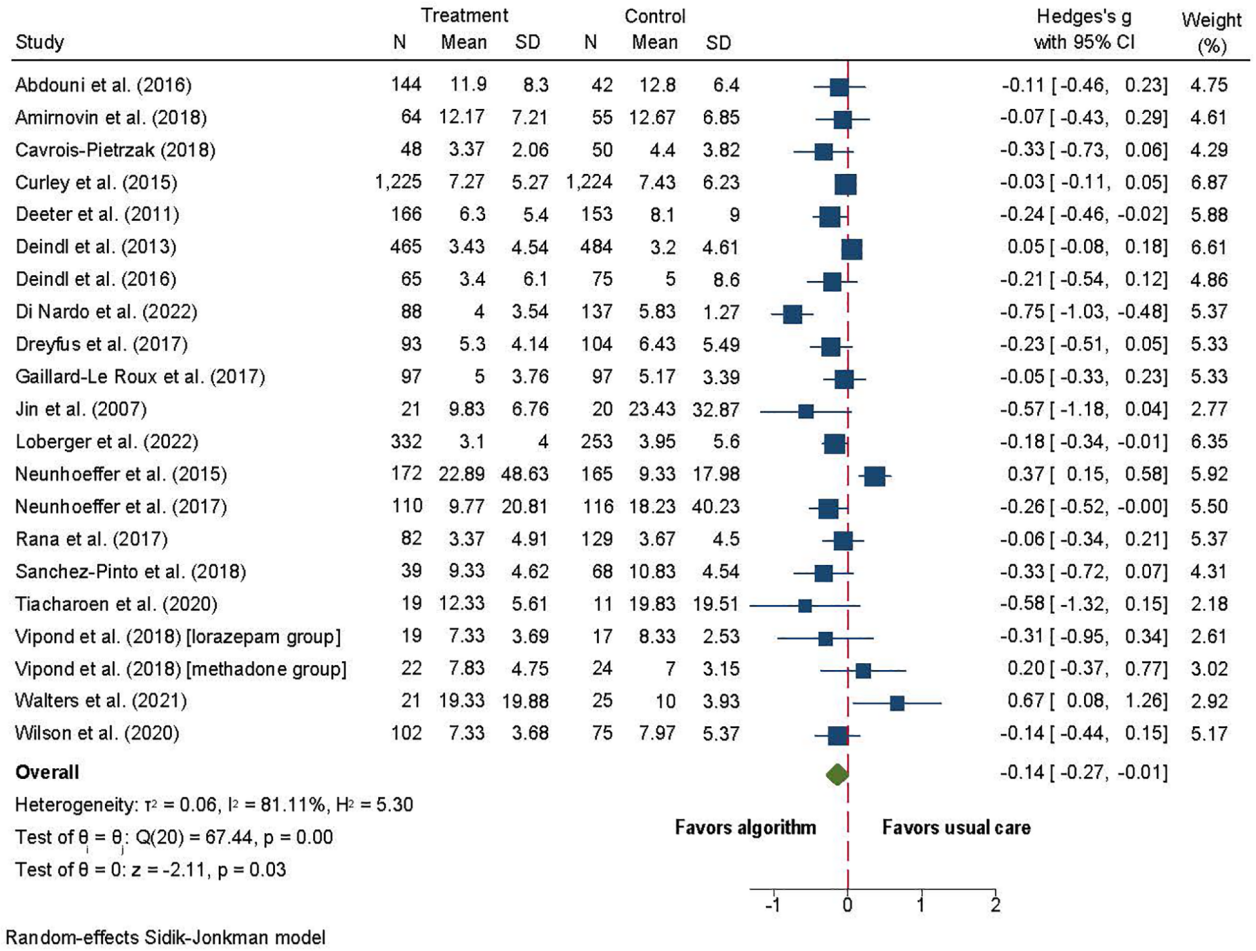
Forest plot for length of mechanical ventilation (days) algorithm versus usual care.

Twenty-six studies included duration of MV (62–64, 66, 70–80, 82–85, 87, 89–92, 94, 95), of which two were excluded due to missing data (83, 84). Four studies were analyzed separately because duration of MV was reported in hours (63, 74, 80, 85). Of twenty studies, the algorithms group showed a small decrease in time on MV (SMD = −0.14; 95% CI = −0.27 to −0.01; *p* = 0.03; Figure 4) (62, 64, 66, 70–73, 75–79, 82, 87, 89–92, 94, 95) compared to usual care. Substantial heterogeneity was observed (*I*^2^ = 81%).

The four studies that reported MV in hours showed no difference in the length of MV between the algorithm and usual care groups (SMD = −0.11; 95% CI = −0.49 to 0.27; *p* = 0.58) (63, 74, 80, 85). Substantial heterogeneity was observed (*I*^2^ = 81). Sensitivity analysis showed that Larson et al. (85) was an outlier, and when removed *I*^2^ was reduced to 0. A small statistically significant effect in hours of MV was observed (SMD = −0.30; 95% CI = −0.46 to −0.15; *p* = 0.0001; Figure not shown).

One study with missing data and showed no significant difference between the algorithm and the usual care groups (84).

#### 3.3.4. Cumulative dose of analgesic medications

**Figure 5:**
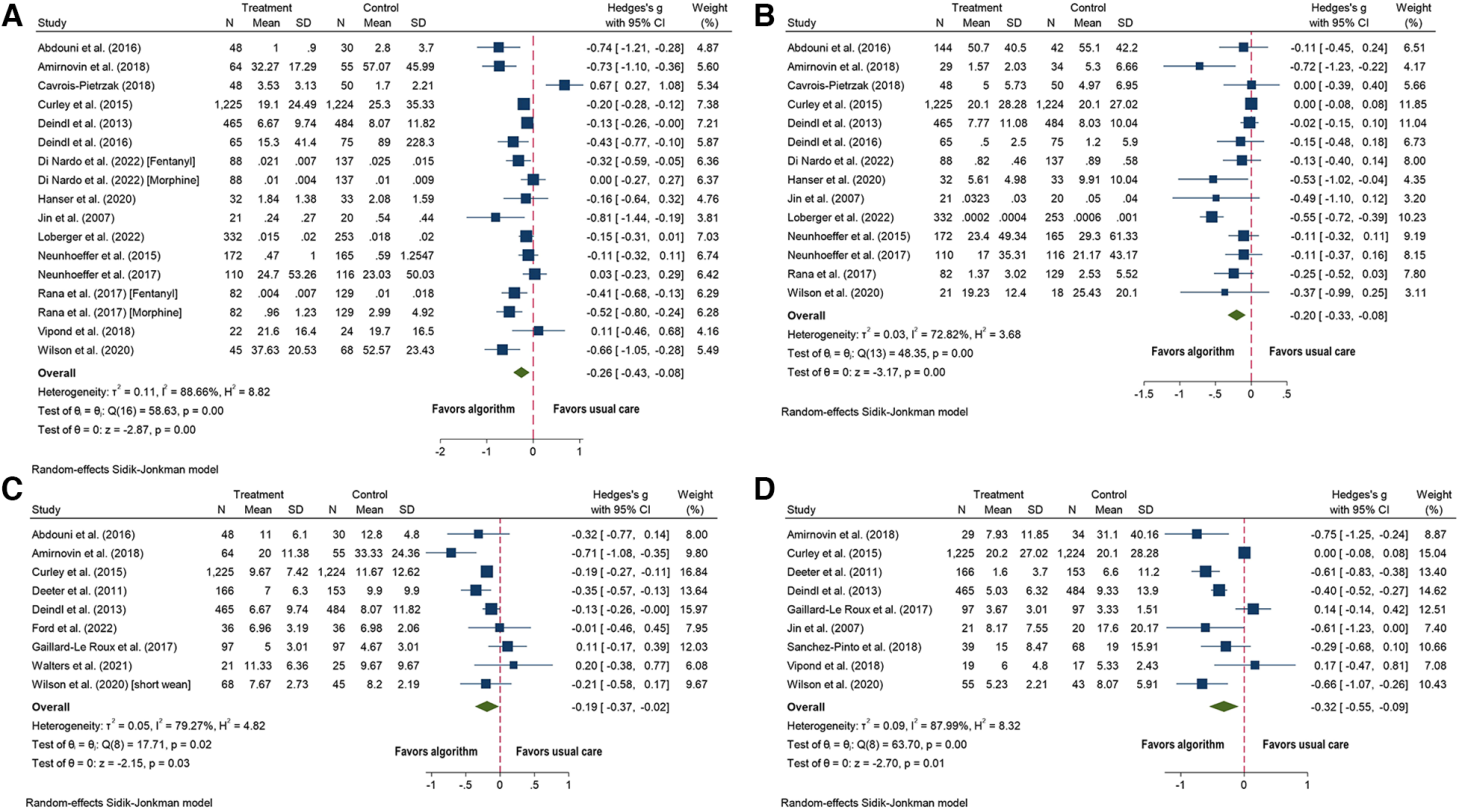
Forest plots for algorithm versus usual care for the outcomes of: (**A**) cumulative dose of analgesic medications; (**B**) cumulative dose of sedative medications: (**C**) duration of analgesics medications (days); and (**D**) duration of sedative medications (days).

Nineteen studies included cumulative dose of analgesic medications (62, 64, 66, 70–73, 78–80, 82, 84, 87, 90, 92–95). Four studies were excluded due to measuring outcomes differently: per visit (74), per patient (72) instead of over time, cumulative dose for the first 12 h, instead of the entire admission period (84), and Yang et al. included data in box plots (93). Of the 15 studies; the algorithm group showed a decrease in the cumulative dose of analgesic medications (SMD = −0.26; 95% CI = −0.43 to −0.08; *p* = 0.0001; Figure 5A) (62, 64, 66, 70, 71, 73, 78–80, 82, 87, 90, 92, 94, 95) compared to usual care. Considerable heterogeneity was observed (*I*^2^ = 89).

Of the four studies not included in the meta-analysis, one study showed a statistically significant decrease in the cumulative morphine dose (mg/kg/visit) in the algorithm group (74), while the other three studies showed no differences between the algorithm and usual care groups (72, 84, 93).

#### 3.3.5. Cumulative dose of sedative medications

Eighteen studies included the outcome of cumulative dose of sedative medications (62, 64, 66, 70–74, 78–80, 82, 84, 87, 89, 92–95). Three studies were excluded because the outcomes were measured differently: per visit (74), per patient (72), per hour instead of days (84). Yang et al. included data in box plots, so was not included (93). Of 14 studies; the algorithm group showed a decrease in the cumulative dose of sedative medications (SMD = −0.20; 95% CI = −0.33 to −0.08; *p* = 0.0001; Figure 5B) (62, 64, 66, 70, 71, 73, 78–80, 82, 87, 92, 94, 95) compared to usual care. Substantial heterogeneity was observed (*I*^2^ = 73).

Three studies not included in the meta-analysis showed statistically significant decreases in the algorithm group: one in cumulative lorazepam dose (mg/kg/visit), another in total dose of midazolam (displayed as box plots) (93), and the last in cumulative dose of midazolam per hour (74). The study by Sanchez-Pinto et al. showed no statistically significant difference between the algorithm and the usual care groups (72).

#### 3.3.6. Duration of analgesic medications

Twelve studies evaluated the duration of use of analgesic medications (62, 65, 70, 71, 73, 75–77, 83, 91, 92, 95). Three studies were excluded, one due to missing data (83), and two for measuring duration in hours instead of days (73, 76). Of nine studies; the algorithm group showed a decrease in the number of days of analgesic administration (SMD = −0.19; 95% CI = −0.37 to −0.02; *p* = 0.03; Figure 5C) (62, 65, 70, 71, 75, 77, 91, 92, 95) compared to usual care. Substantial heterogeneity was observed (*I*^2^ = 79).

Three studies not included in the meta-analysis showed no statistically significant differences between the algorithm and the usual care groups (73, 76, 83).

#### 3.3.7. Duration of sedative medications

Fifteen studies included the duration of sedative medications (62, 71–73, 75–77, 81–83, 86, 89, 90, 92, 95). Three studies were excluded because of missing data (81, 83, 86). Three additional studies were excluded, two for measuring duration in hours instead of days (73, 76), and one for measuring duration of sedative medications per patient (72). Of nine studies; the algorithm group showed a decrease in the number of days sedatives were administered (SMD = −0.32, 95% CI = −0.55 to −0.09; *p* = 0.01; Figure 5D) compared to usual care (62, 71, 72, 75, 77, 82, 90, 92, 95). Heterogeneity was substantial (*I*^2^ = 88).

Three studies not included in the meta-analysis showed no statistically significant difference between the algorithm and usual care groups when measuring duration in hours instead of days (73, 76, 83). Hawzani et al. included run charts and showed a significant decrease in the hours of sedative use over the course of algorithm implementation (81). Puthoff et al. did not include the data for the control group but noted a statistically significant decrease in days on benzodiazepines (86).

#### 3.3.8. Incidents of withdrawal

**Figure 6:**
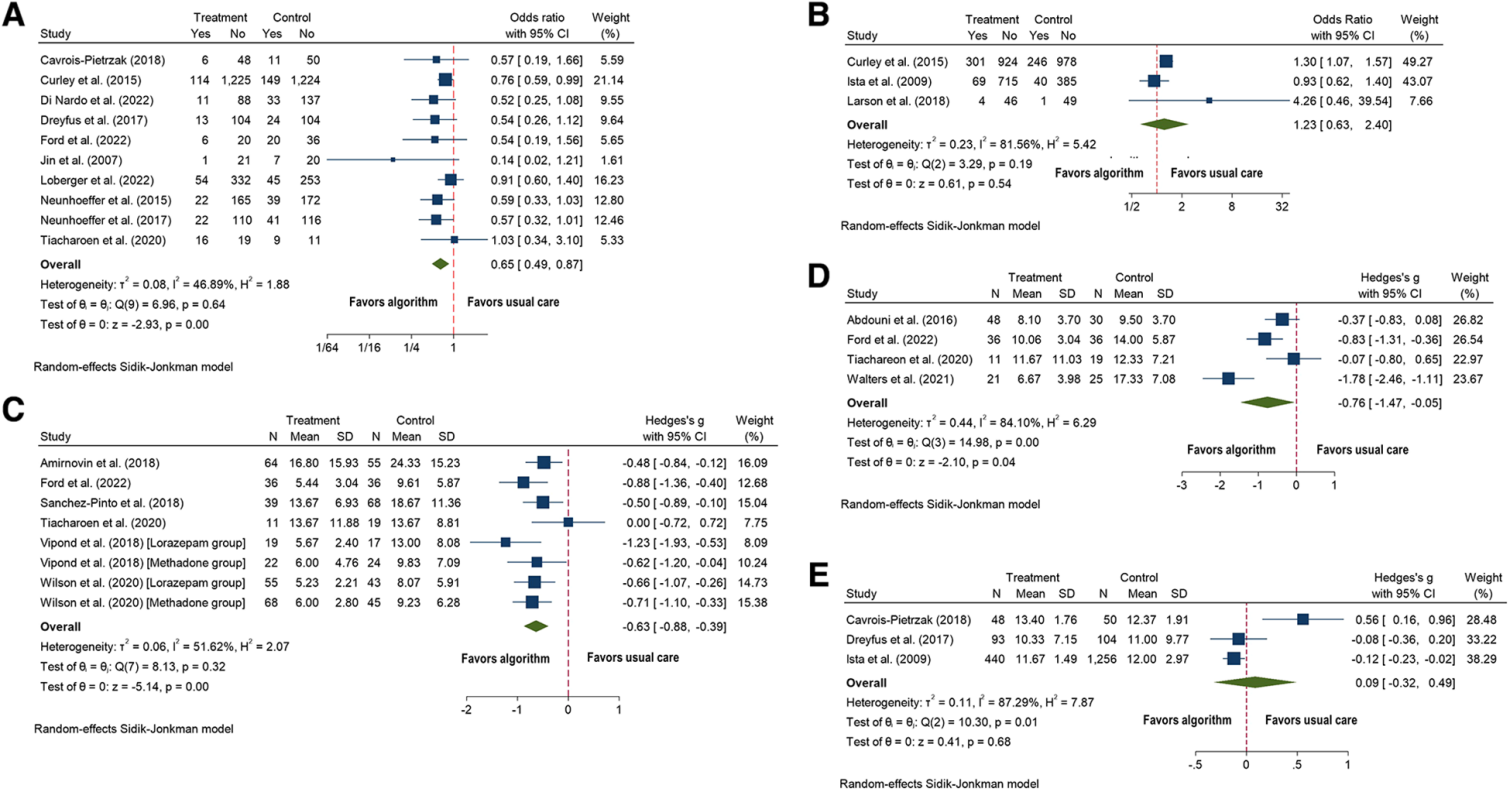
Forest plots for algorithm versus usual care for the outcomes of: (**A**) incidents of withdrawal; (**B**) inadequate sedation management (under-sedation): (**C**) duration of medication weaning (days); (**D**) duration of methadone exposure (days), and (**E**) mean COMFORT-B score.

Thirteen algorithms reported the percentage of patients with IWS symptoms (62, 64–66, 71–73, 76–79, 82, 89). Ten studies included the event of developing IWS for both groups, and the analysis showed that the odds of experiencing IWS were reduced by 35% for children in the algorithm group (OR 0.65; CI = 0.49 to 0.87; *p* < .0001; *I*^2^ = 47; Figure 6A) (62, 64–66, 73, 76, 78, 79, 82, 89) compared to usual care.

Two studies not included in the meta-analysis showed a decrease in the rates of IWS in the algorithm compared to the usual care group (71, 82), and the remaining five showed no significant difference between the groups (72, 73, 76, 77, 92).

#### 3.3.9. Incidents of delirium

One study reported on the treatment of delirium with antipsychotics and there was no significant difference between the two groups (*p* = 0.09) (64).

#### 3.3.10. Inadequate sedation management

Seven studies reported on inadequate sedation management (61, 62, 76–78, 80, 85). One study was excluded because it did not report total numbers (76); two reported only on the post-implementation period (78, 80); and another did not include data (77). Of three studies, no difference in under-sedation was observed between the two groups (OR 1.23; CI = 0.63–2.40; *p* = 0.54; *I*^2^ = 82; Figure 6B) (61, 62, 85).

Sensitivity and subgroup analyses were not performed because there were only three studies.

Two studies reported on over-sedation and were not statistically significant but had opposite directions of effect, one with more incidents of over-sedation in the algorithm group (85) and the other with more incidents in the usual care group (61). Dreyfus et al. found a decrease in the mean number of over-sedation levels in the algorithm group (76). Gaillard et al. stated no significant difference was observed between groups (77).

#### 3.3.11. Inadequate pain management

Three studies reported on inadequate pain management (62, 76, 95), but a meta-analysis could not be performed due to the difference in data. One study reported only post-implementation results (95). One study reported no difference between the two groups (62), while another showed an increase in the number of adequate mean pain levels documented (76).

#### 3.3.12. Duration of medication weaning and methadone use

Six studies reported on the duration of weaning, and all were included in the analysis (65, 71, 72, 89, 90, 92). The algorithm group had fewer days of weaning from medications (SMD = −0.63, 95% CI = −0.88 to −0.39; *p* = 0.0001; Figure 6C) than the usual care group. Heterogeneity was moderate (*I*^2^ = 52).

Four studies reported the duration of methadone exposure and were all included in the analysis (65, 70, 89, 91). The algorithm group had fewer days of methadone exposure (SMD = −0.76, 95% CI = −1.78 to −0.05; *p* = 0.04; Figure 6D) than the usual care group. Heterogeneity was substantial (*I*^2^ = 84).

#### 3.3.13. Scores for pain, sedation and withdrawal

Two studies reported on pain scores (62, 74). Curley et al. reported that the intervention group had a greater percentage of days with any report of a pain score of 4 or higher compared to the control group (*p* < .001); however, there was no difference between the two groups for modal pain scores of less than 4 (62). Lincoln et al. reported no significant differences in pain scores between the groups (74).

Nine studies reported on sedation scores (61, 64, 73, 74, 76, 77, 81, 85, 93). Three studies reported median COMFORT-B scores per patient, and showed no statistically significant difference between the algorithm (SMD = 0.09, 95% CI = −0.32 to 0.49; *p* = 0.68; *I*^2^ = 87; Figure 6E) and the usual care groups (61, 73, 76).

Among the studies not included in the meta-analysis. One study reported the percentage of COMFORT-B scores per patient increased significantly from 40% in the usual care group to 75%-85% in the algorithm group (81). Three studies that implemented a “*pain + sedation*” algorithm reported a significant increase in the number of COMFORT-B assessments completed daily (73, 76, 85). Two studies reported no difference between the number of assessments per day, one using the COMFORT-B (77) and another using the SBS (74). Three studies using the SBS reported no statistically significant difference between SBS scores before and after implementation (64, 74, 93).

Two studies reported on withdrawal scores (62, 74); both showed no difference between groups, one in peak WAT-1 scores (62), and the other in mean WAT-1 scores (74).

#### 3.3.14. Subgroup analyses

The subgroup analyses by algorithm type indicated no statistically significant subgroup effects for cumulative dose of sedative medications, duration of analgesic medications and methadone exposure (Supplementary Figures S1–S8), suggesting that algorithm type does not modify the effect of algorithms compared to usual care. However, the small number of studies and participants in some subgroups may indicate that the analysis may have been unable to detect subgroup differences.

There were some differences observed by algorithm subgroup types:

• IWS algorithms showed a decrease in length of hospital stay (SMD = −0.34; 95% CI = −0.64 to −0.03; *p* = 0.08; *I*^2 ^= 60); a decrease in the cumulative dose of analgesic medications (SMD = −0.54; 95% CI = −0.93 to −0.15; *p* = 0.07; *I*^2^ = 69%) (70, 71, 90, 92); and a decrease in the number of days analgesic medications were administered (SMD = −0.41; 95% CI = −0.82 to −0.01; *I*^2^ = 65; *p* = 0.09) (71, 72, 90, 92).
• *“Pain-sedation”* algorithms showed a decrease in length of MV (SMD = −0.13; 95% CI = −0.25 to −0.02; *p* = 0.10; *I*^2^ = 41) (64, 73, 75–77, 94, 95).
• “*Pain-sedation-IWS*” algorithms showed a decrease in the cumulative dose of analgesic medications (SMD = −0.14; 95% CI = −0.26 to −0.03; *p* = 0.34; *I*^2^ = 28%) (62, 78–80); and a decrease in incidents of IWS (OR = −0.48; 95% CI = −0.80 to −0.16; *I*^2^ = 28; *p* = 0.29) (62, 78, 79).

#### 3.3.15. Summary of findings

The summary of findings table for the seven key outcomes, using GRADE, is presented in Table 1.

**Table 1:** GRADE summary of findings.

**Patient**: Pediatric patients, from 23 weeks gestation to 18 years of age **Setting**: Pediatric and neonatal intensive care units **Intervention**: Algorithm for the management of pain, sedation, delirium, and IWS **Comparison**: Usual care						
**Outcomes**	Illustrative comparative risks* **(95% CI)**		**Relative effect (95% CI)**	**No of participants (studies)**	**Certainty of evidence (GRADE)**	**Comment**
	**Assumed risk**	**Corresponding risk**				
	**Usual care**	**Algorithm**				
**Intensive care LOS** (days)	The SMD ranged from **4 to 72 days**	The SMD was **0.13 fewer days** (0.22 to 0.05 fewer)	**_**	*N* = 7,524 (17 Q-E; 2 RCT; 6 cohort)	**VERY LOW** ⨁OOO^1,2^	There may be little or no difference in intensive care LOS
**Duration of MV** (days)	The SMD ranged from **3 to 23 days**	The SMD was **0.14 fewer days** (0.27 to 0.01 fewer)	**_**	*N* = 6,718 (2 RCT, 13 Q-E; 5 cohort)	**VERY LOW** ⨁OOO^1,2^	There may be little or no difference in duration of MV
**Duration of analgesics** (days)	The SMD ranged from **4.5 to 33 days**	The SMD was **0.19 fewer days** (0.37 to 0.02 fewer)	**_**	*N* = 4,318 (1 RCT, 4 Q-E, 4 cohort)	**VERY LOW** ⨁OOO^1,2^	There may be little or no difference in duration of analgesics
**Duration of sedatives** (days)	The SMD ranged from **5 to 31 days**	The SMD was **0.32 fewer days** (0.55 to 0.09 fewer)	**_**	*N* = 4,256 (1 RCT, 5 Q-E, 3 cohort)	**VERY LOW** ⨁OOO^1,2^	There may be a decrease in duration of sedatives
**Cumulative dose analgesic medications** (mg/kg/day)	The SMD ranged from **0 to 57 mg/kg/days**	The SMD was **0.26 fewer mg/kg/days** (0.43 to 0.08 fewer)	**_**	*N* = 6,118 (1 RCT, 11 Q-E, 3 cohort)	**VERY LOW** ⨁OOO^1,2^	There may be a decrease in the cumulative dose of analgesic medications
**Cumulative dose sedative medications** (mg/kg/day)	The SMD ranged from **0 to 55 mg/kg/days**	The SMD was **0.20 fewer mg/kg/days** (0.33 to 0.08 fewer)	**_**	*N* = 5,614 (1 RCT, 11 Q-E, 2 cohort)	**VERY LOW** ⨁OOO^1,2^	There may be a decrease in the cumulative dose of sedative medications
**Incidents of withdrawal**	178 per 1,000	**123 per 1,000** (96 to 159)	**OR 0.65** (0.49 to 0.87)	*N* = 4,255 (2 RCT, 7 Q-E, 1 cohort)	**VERY LOW** ⨁OOO^2,3^	There may be a decrease in the incidents of withdrawal

**SMD**, standardized mean difference; **CI**, confidence interval; **OR**, odds ratio, **LOS**, length of stay; **MV**, mechanical ventilation; **RCT**, randomized controlled trial; **Q-E**, quasi-experimental.

GRADE (Grading of Recommendations, Assessment, Developments and Evaluation) Working Group grades of evidence:.

**High quality**: Further research is very unlikely to change our confidence in the estimate of effect.

**Moderate quality**: Further research is very likely to have an important impact on our confidence in the estimate of effect and may change the estimate.

**Low Quality**: Further research is very likely to have an important impact on our confidence in the estimate of effect and is likely to change the estimate.

**Very low quality**: Any estimate of effect is uncertain.

* The basis for the **assumed risk** (e.g. the median control group risk across studies) is provided in footnotes. The **corresponding risk** (and its 95% confidence interval) is based on the assumed risk in the comparison group and the **relative effect** of the intervention (and its 95% CI).

Explanations.

^1^ Quality of evidence downgraded one level for inconsistency of the estimates due to considerable unexplained heterogeneity (*I*^2^ > 40%).

^2^ Quality of evidence downgraded one level for indirectness of the population.

^3^ Quality of evidence downgraded one level for imprecision of the estimates as the CI crosses the appreciable effect line (0.75).

#### 3.3.16. Publication bias

No publication bias was observed for any of the outcomes with more than 10 studies (Supplementary Figure S9).

#### 3.3.17. Sensitivity analysis

The sensitivity analyses of removing one study at a time showed no significant reduction in heterogeneity for all but three outcomes of interest. Firstly, for length of MV, three studies individually decreased the heterogeneity by 2%–7% (66, 78, 91) but removed together, *I*^2^ decreased by 30% with the same small-sized effect (SMD = −0.14; 95% CI = −0.23 to −0.05; *p* = 0.001; *I*^2 ^= 51). Secondly, for duration of analgesic medications, two studies were outliers (71, 77) with no significant decrease in heterogeneity when individually removed, but when removed together, *I*^2^ decreased by 24% (SMD = −0.18; 95% CI = −0.31 to −0.05; *p* = 0.01; *I*^2^ = 55). Lastly, for incidence of withdrawal where one study (82) when removed decreased the *I*^2^ by 28% with a slight effect size change (OR 0.69; CI = 0.56 to 0.86; *p* < .0001; *I*^2^ = 19) (Supplementary Figures S10–S20).

Sensitivity analysis by type of setting showed a decrease in LOS ICU for PICUs (SMD = −0.12; 95% CI = −0.22 to −0.02; *p* = 0.34; I2 = 53%) but not for NICUs, and a decrease in the cumulative dose of analgesic medications for NICUs (SMD = −0.33; 95% CI = −0.52 to −0.15; *p* = 0.03; *I*^2^ = 57%) but not for PICUs. The test of group difference was not significant for either analysis (results not shown).

Sensitivity analyses by setting could not be performed for the outcomes of hospital LOS, duration of analgesic medications, duration of sedative medications, and incidents of withdrawal because there were no NICUs or >2 studies.

All sensitivity analyses by study design showed that quasi-experimental studies favored algorithms (results not shown).

### 3.4. Algorithm quality and evidence

The overall PROFILE and the three process scores of the 28 algorithms are displayed in Table 2.

**Table 2:** Heatmap PROFILE scores.

Algorithm focus	Author	Process scores			Overall (24)
		Development (15)	Content (5)	Implementation (4)	
*P*	Rana (87)	6	2	0	8
*S*	Hazwani (81)	8	3	4	15
*W*	Abdouni (70)	10	5	4	19
	Amirnovin (71), Sanchez-Pinto (72)	5	4	1	10
	Ford (65)	12	5	3	20
	Tiachareon (89)	4	3	0	7
	Vipond (90)	7	3	1	11
	Walters (91)	3	5	0	8
	Wilson (92)	6	3	1	10
*P-S*	Cavrois-Pietrzak (73)	5	4	4	13
	Deeter (75)	8	3	2	13
	Deindl (94, 95)	5	3	3	11
	Dreyfus (76)	3	4	1	8
	Gaillard-Le Roux (77)	5	4	1	10
	Ista (61)	10	5	3	18
	Kleiber (84)	2	3	1	6
	Larson (85)	4	4	2	10
	Loberger (64)	8	5	4	17
	Magner (63)	15	5	4	24
	Puthoff (86)	7	4	4	15
	Yang (93)	9	4	2	15
*P-S-W*	Curley (62)	11	5	4	20
	Keogh (83)	11	4	2	17
	Lincoln(74)	5	3	2	10
	Neunheoffer (78, 79), Hanser (80)	5	4	1	10
*P-S-D-W*	Di Nardo (66)	11	3	4	18
*S-W*	Jin (82)	3	3	0	6
*P-W*	Stetson (88)	6	4	4	14
Threshold ranges					
High	>60%	≥9	≥3		≥15
Medium	30%–59%	5–8	2		8–14
Low	<30%	≤4	≤1		≤7

P, pain; S, sedation; W, iatrogenic withdrawal; D, delirium.

The mean percentage of the overall score was 54%. Eleven algorithms scored as high (39%) (61–66, 70, 81, 83, 86, 93), 14 as medium (50%) (71–80, 85, 87, 88, 90–92, 94, 95), and three as low (11%) (82, 84, 89).

The mean percentage of the development process score was 46%. Eight algorithms scored as high (29%) (61–63, 65, 66, 70, 83, 93), 14 as medium (50%) (64, 71–81, 86–88, 90, 92, 94, 95), and six as low (21%) (76, 82, 84, 85, 89, 91).

The mean percentage of the content process score was 76%. All but one algorithm scored as high (61–66, 70–86, 88–95), and it was medium (87).

The mean percentage of the implementation process score was 55%. Twelve algorithms scored as high (43%) (61–66, 70, 73, 81, 86, 88, 94, 95), five as medium (18%) (74, 75, 83, 85, 93), and 11 as low (39%) (71, 72, 76–80, 82, 84, 87, 89–92).

Among the five domains of the PROFILE, Domain 1 (scope and purpose) scored high for all algorithms, with at least two out of the three items being present. Domains 4 and 5 were discussed in the previous section and are related to two of the three process scores (content and implementation process scores, respectively). The lowest represented domain was Domain 2 (stakeholders), with only one algorithm including the patient or family, during development (63). Details of each item by algorithm are in Supplementary Table S12.

Concerning content and development, 15 algorithms were developed because of an identified clinical problem (54%) (61–64, 66, 70, 73, 81, 83, 85, 86, 88, 90, 93–95). Eighteen algorithms reported using an interdisciplinary approach to development (64%) (61–64, 66, 70, 73–75, 77–81, 85–88, 93–95). Eighteen algorithms reported using evidence during development (64%) (61–63, 66, 70, 75, 77–81, 83, 87, 89, 90, 92–95), with four (14%) using a CPG as the highest quality of evidence (63, 73, 83, 93).

### 3.5. Implementation process

#### 3.5.1. Implementation strategies

Implementation strategies were reported in 26 of 32 studies (61–63, 65, 66, 70–78, 81, 83–88, 91–93, 95). Of the 17 EPOC categories, 13 were used (76.5%), the details are presented in Supplementary Table S13. Apart from one study (91), all other studies applied multiple strategies. The most frequently applied strategies were educational meetings (*n* = 24, 92%) (61–63, 70–78, 81, 83–88, 91–93, 95), the provision of materials (*n* = 24, 92%) (61–63, 65, 66, 70–78, 81, 83, 85–88, 92, 93, 95), followed by out-reach visits (*n* = 13, 50%) (62, 63, 66, 70, 71, 75, 77, 78, 81, 83, 84, 93, 95). The most frequently used non-educational strategy was continuous quality improvement (*n* = 12, 46%) (63, 70–75, 81, 86, 88, 93, 95). The least frequently used implementation strategies were audit and feedback (*n* = 2, 8%) (62, 81), tailored interventions (*n* = 2, 8%) (70, 81), use of local champions (*n* = 1, 4%) (62), managerial supervision (*n* = 1, 4%) (63), and monitoring performance (*n* = 1, 4%) (65). The mean number of implementation strategies used across all studies was 4.2 (median = 3.5, IQR 2). High-quality algorithms used a mean of 5.4 (median = 4.5, IQR 5) implementation strategies, compared to medium- and low-quality algorithms, with a mean of 3.4 (median = 3.5, IQR 2.5).

#### 3.5.2. Determinants

Fifty-five determinants (barriers or facilitators) were reported across ten studies (61, 66, 70, 73, 81, 83, 88, 90, 95) and grouped into 48 unique determinants. Five studies measured determinants (66, 73, 81, 83, 90), three measured determinants quantitatively using surveys (66, 83, 90), and two measured determinants qualitatively (73, 81). Supplementary Table S14 presents the organizational, professional, and interventional determinants.

The major organizational barriers were lack of leadership support, lack of planning for training, and competing priorities. The organizational facilitators were team buy-in, support, and involvement.

The major professional barriers relate to the complexity of applying algorithms to patients due to stability and age, and nurses' lack of knowledge of the algorithm. Professional facilitators included education and a positive attitude.

The major intervention facilitators were the structure of the algorithm, automatic alerts for assessment, and support for decision-making, resulting in ease and efficiency. No intervention barriers were reported.

#### 3.5.3. Fidelity (adherence and dose of the intervention)

Fidelity to the algorithm was reported in 19 studies (68%) (61–63, 66, 70–73, 77–79, 81, 83, 85, 87, 90, 92, 93, 95). Five studies reported both adherence to the algorithm and the dose of algorithm components (26%) (62, 66, 70, 83, 87). Eight studies reported on adherence (42%) (71–73, 78, 79, 81, 90, 93) and six reported on the dose of delivery of components (32%) (61, 63, 77, 85, 92, 95). The dose category had two overarching sub-categories: (1) dose related to the use of the measurement instrument, reported by eight studies (63, 66, 71, 73, 77, 83, 85, 95), and (2) dose related to medication delivery reported by four studies (61, 63, 87, 92). Overall adherence varied considerably, ranging from 36% to 100%. The dose associated with using the measurement instrument across all conditions was high, ranging from 65% for IWS (62) to 95.9% for pain and sedation (77). One study reported adherence to medication at 61% (63), and two studies reported loading dose delivery ranging from 8.2 to 86% (63, 92). One study reported both bolus prescription and medication prescription delivery with a 90.6% adherence rate (63). Supplementary Table S15 presents details of fidelity.

#### 3.5.4. Satisfaction with algorithms

Six studies reported on staff satisfaction levels (63, 66, 74, 83, 90, 95) and one on parental satisfaction (87) (Supplementary Table S16). All studies with pre- and post-implementation statistics supported improvements; however, physicians showed greater satisfaction, as compared to nurses. Lincoln did not include a percentage score, instead indicated nurses' satisfaction with patient sedation management post-implementation was 8.5 (74).

## 4. Discussion

To the best of our knowledge, this was the first systematic review that examined the effectiveness of 28 algorithms for the management of pain, sedation, delirium and IWS in pediatric and neonatal intensive care. There were four key findings; firstly, standardized algorithm-based management across the four conditions had a weak but positive effect on improving most outcomes of interest for critically ill children in pediatric intensive care settings. Secondly, the evidence base used to develop algorithms varied with inconsistent multidisciplinary and little patient and family involvement in development and implementation. Thirdly, implementation processes were poorly described. Fourthly, although, adherence rates varied, they showed improved assessment and documentation practices.

Standardizing the management of pain, sedation, delirium and IWS using an algorithm is recommended as a strategy (3, 20); however, evidence for its effectiveness is limited and restricted to sedation management algorithms (24, 26). This review was designed to overcome this challenge by including the four interlinked conditions of pain, sedation, delirium, and IWS. The negative outcome of IWS related to prolonged analgesic and sedative exposure in pediatric intensive care has long been recognized (1, 5). The results of these meta-analyses of algorithms compared with usual care showed an impact on several outcomes of interest, with a significant reduction in IWS and small decreases in the length of ICU and hospital stays, length of MV, cumulative dose and duration of analgesics and sedative medications, and duration of weaning from medications and methadone exposure. The results showed moderate to substantial heterogeneity, which is expected given the variations in study design, the algorithms, and in the populations of pediatric intensive care settings. These varying factors may have contributed to the small effective sizes observed but potential sources were investigated using sensitivity analyses. These results were similar to those reported in other reviews of sedation algorithms, demonstrating that patients receiving more effective sedation resulted in decreased LOS and cost (24, 25). One study demonstrated a cost savings of approximately $17,000 per patient when an IWS algorithm was used (71). Delirium bundles were designed as a preventive strategy, and although they include delirium screening, the results do not have a described course of action, and as such were excluded from this review. This explained why delirium is poorly integrated in algorithms. A recently published systematic review and meta-analysis (96) suggests, however, the need for prevention and the results from this review supported their inclusion and incorporation into algorithms that standardized care for critically ill children. Protocolized management of pain, sedation and weaning were recommended in the recently published PANDEM CPG (14). The small decrease in both duration and cumulative dose of sedatives per day in this review is promising, as the association with the development of delirium and withdrawal is undisputed.

In this study, over a third of the algorithms were scored as high-quality, indicating their potential to improve the management of the four conditions. However, given the undisputed benefits of CPGs, the lack of their use in the development of algorithms was an important finding. Out of 28 algorithms, only four used CPGs during development, suggesting that CPGs are not fully utilized, possibly due to lack of awareness or trust among HCPs. This finding was consistent with two recent surveys of PICUs showing that PICU practices related to pain and sedations management varied widely across Europe (19, 97). Out of the 215 PICUs surveyed, only 71% reported having a protocol in place (19) and the implementation of measurement instruments for the four conditions was inconsistent (97). The lack use of CPGs in the development of algorithms and the inconsistency in implementing measurement instruments for the four conditions highlighted the need for more standardized practices, better implementation of measurement instruments and CPGs. While some algorithms scored as high-quality, it is concerning that many algorithms relied on expert opinion rather than rigorous EB CPGs. Even when CPGs are rigorously developed, they do not consider the local needs. Therefore, they should be integrated into internal unit specific algorithms (98, 99) to ensure that management practices are standardized and used consistently, that decision-making and actions are EB and clearly defined. Furthermore, the wide international variation in practices related to pain and sedation underscored the need for greater attention to the issues of CPG, algorithm, and measurement instrument implementation. The use of non-validated scoring ranges (84–86) or combining measurement instruments (i.e., Comfort-B + NISS (61, 63, 78–80), as observed in some algorithms, was not supported in a recent EB CPG and is problematic, as it could render the evaluation invalid. Therefore, it is important to continue to improve the quality of algorithms using EB CPGs. While guidelines are well-established, the ideal sedation and analgesia regime for pediatric patients remains undefined, and their application in practice is challenging. Algorithms have been proposed to overcome this gap and provide standardized, EB care. Although many studies reported contradictory results, pooling them together revealed a small effect, indicating that algorithms can contribute to delivering consistent and EB care for pediatric patients.

In this review, barriers to the implementation process of algorithms in pediatric intensive care settings were identified. However, the implementation processes were often poorly reported, highlighting the importance of robust implementation processes as a barrier to transferability. Implementation strategies for algorithms tended to focus on education and training strategies, but these are often considered insufficient for successful implementation (100). Other studies have also emphasized the importance of education and training in the implementation process (34, 101). High-quality algorithms tended to utilize more implementation strategies compared to medium- and low-quality algorithms, suggesting a potential link between the number and types of strategies used and successful implementation. Furthermore, studies in this review that examined determinants emphasized the significance of continuous quality improvement, local outreach, and local opinion leaders (champions) in the implementation process. Although, satisfaction with the implementation of algorithms was not widely reported, increasing levels were noted for HCPs and families.

The review identified organizational barriers, such as planning and leadership support, as crucial facilitators, which is consistent with existing literature (34). It also found that ensuring seamless integration of algorithms into the electronic health system is a crucial consideration. Given the increasing digitalization of healthcare, several studies have described the need for optimizing the design and ensuring integration into the workflow, as this can impact the use and burden nurses (102). Involving staff in technology design (103, 104) and implementation planning was recognized as a necessary strategy for improving implementation and sustainability efforts (104), which aligns with many process models in implementation science (105). Although, no studies directly evaluated algorithm implementation into the electronic health record, one study evaluating an integrated rounding checklist in the PICU showed improved outcomes for patients across many of the measured outcomes (106). During implementation planning, the identification of barriers and facilitators is undisputed to facilitate implementation efforts, as highlighted by determinant frameworks (105). However, many studies identified mostly barriers post-implementation or were researcher perceived, and those that identified them pre-implementation did not map specific strategies to those identified.

In terms of professional barriers, this review highlighted the importance of planning for training, leadership support, and team buy-in to supporting the implementation process. This is supported by a study that described knowledge translation strategies used across 16 pediatric units, including neonatal and pediatric ICUs (101). Algorithms can be difficult to update and maintain over time (107, 108), but these challenges can be overcome by utilizing and maintaining more complex implementation strategies, such as champions, continuous quality improvement, and auditing and monitoring (34). While few studies in this review reported on fidelity, studies with high-quality algorithms more often reported favorable rates. Improved understanding of barriers and facilitatos, along with the use of increased implementation strategies, can enhance the likelihood of successful algorithm implementation, particularly, when integrated into the electronic health records as clinical decision support systems (109). A recent qualitative study using the iPARIHS framework also identified several similar barriers and facilitators to practice change in PICUs (34), and these same variables were further supported in a recent realist review (110), suggesting their foundational importance in implementation in pediatric centers. These findings underscore the value of conducting reviews to establish known determinants and strategies that can aid teams during implementation, leading to reduced research waste and faster implementation (30, 31).

The levels of adherence to the algorithm, including the dose, components, and measurement instruments were mixed. However, given the varied use of measurement instruments, increased compliance with assessment recommendations, and limited change in scores, this suggests that embedding measurement instruments into algorithms increases their frequency of use without changing the scores. This demonstrates the adequacy of the management strategies outlined in the algorithm. The use of algorithms improved both assessment and documentation practices, which are often cited as areas of concern by HCPs (111).

### 4.1. Strengths and limitations

An important contribution and strength of this review is the comprehensive evaluation of the development (structure), implementation (process), and outcomes of algorithm use. To the best of our knowledge, this method of evaluation has not been performed in a systematic review. However, this is also a limitation, as many of the algorithms were developed prior to the recommendations for using implementation frameworks and their standardized reporting, such as the template for intervention description and replication (112). Reporting of all aspects may not have been possible in all studies due to a lack of awareness or limitations in word count, as has been identified in other areas of research (113). This may have resulted in lower quality scores for some algorithms. To overcome this limitation, a standardized method was employed to contact all study authors, as recommended by Reynders et al. (114).

Another strength of this review is the recognition of the importance of ensuring the quality of algorithms, which was the reason for developing and using the PROFILE tool. Given the potential variability in algorithm quality and the impact this can have on patient outcomes, the assessment of rigor and transparency of algorithm development is crucial. The PROFILE provides a comprehensive framework for performing evaluations of algorithms and serves to identify areas for improvement in development and reporting. This underscores the need for a standardized approach to algorithm development and evaluation to ensure they are sfe, effective, and reliable.

Another limitation is the possible sources of heterogeneity, including the non-standardized reporting of outcomes, which affected the ability to include outcomes in meta-analyses. Some outcomes had considerable differences, such as the different dosages and forms of medication administration (e.g., continuous administration of one medication or continuous administration that included boluses), and the definitions of the treatment point for the outcomes being examined. Other factors could also have affected the results, such as age, underlying disease and severity, and concomitant medications. Currently, standardized core outcome sets do not exist in the PICU, which likely leads to variability.

The variability in research designs is another limitation. While RCTs are considered the gold standard, they are tightly controlled and conducted with homogenous populations. Therefore, although RCTs may show better treatment effects for outcomes of interest, their results cannot be readily used in the clinical setting where these algorithms are often applied to the entire unit or much broader populations. With unit-based algorithms (quasi-experimental studies), the best possible results are achieved for these children based on continuous assessment and individualized care. However, retrospectively acquired methods may decrease the treatment effects.

### 4.2. Implications and recommendations for practice and research

This systematic review has established that algorithm-based management is potentially associated with reductions in most of the outcomes of interest and improved fidelity to algorithm components, including assessment and documentation across the four conditions. While many of the algorithms contained a mixture of these conditions, only one combined all four conditions (66). Likely due to the complexity of combining all four together, however, many researchers have called for their inclusion (3, 9). In fact one team is currently developing a combined measurement instrument (115), demonstrating both the feasibility and need for combining these four conditions. Teams in pediatric ICUs considering the implementation of an algorithm for the assessment and management of pain, sedation, delirium, and IWS should consider using the currently available algorithms and adapting them to their practice environment if necessary. These teams should incorporate the latest recommendations from quality CPGs (14), as these were missing in many of the included algorithms. Teams should publish their implementation efforts and use the PROFILE (user manual available upon request to author) to ensure complete reporting.

This review provides an overview of the implementation strategies used and the common determinants to consider when developing implementation plans. Implementation teams should consider these determinants in their planning to hasten implementation efforts by focusing on the contextual assessment of missing factors. The review also demonstrates the need to include all outcomes related to the implementation process, such as fidelity, to interpret successful implementation efforts or ineffective interventions. The use of implementation frameworks to guide the process is essential for advancing algorithms into practice (34, 116), along with the use of systematic reviews (30). Applying Donabedian's outcomes to the implementation process would help researchers and healthcare teams assess the required aspects to explain the implementation process of complex health interventions.

This review highlights the limited inclusion of patients and families in both algorithm development and implementation planning, and it is recommended that teams consider their inclusion throughout the process. Patient and family involvement has been recognized as integral in CPG development (117), and it is likely transferable to algorithm development. Additionally, the inclusion of staff throughout the process was also haphazard, and their inclusion is crucial for success.

Although the implementation of an algorithm for the management of pain, sedation, delirium, and IWS is an important first step for critically ill children, a cultural shift that includes the ABCDEF bundles is important (97, 118), along with engaging patients and families through family-centered care (119, 120).

This review highlights that many patient outcomes are not defined, and those that are vary in the depth of their definitions, making them difficult to pool. This study further supports the need for researchers to develop a PICU-specific core outcome set to examine effectiveness.

As indicated in the review of clinical pathway effectiveness, this study confirms the need for future systematic reviews to group algorithms and clinical pathways by condition to reduce heterogeneity (28). Both reviews highlight the need for clinical pathway and algorithm integration into clinical decision support systems. This was a facilitator indicated in this review and has been supported by other studies (109). Further research is needed to understand technology embeddedness in the development and implementation of complex healthcare interventions.

## 5. Conclusion

This comprehensive systematic review provides valuable insight and a much-needed evaluation of the use of algorithms for the management of pain, sedation, delirium, and IWS in children in pediatric intensive care settings. The study results demonstrate that the implementation of algorithms can lead to improved patient outcomes and increased adherence to EB practices and documentation. Algorithms not only improve outcomes but also ensure standardization, preventing important interventions from being missed and ensure timely and appropriate treatments are applied, leading to improved documentation and satisfaction of HCPs. However, this review has highlighted gaps in the processes and reporting of algorithm development and implementation. The implementation of algorithms can be challenging, and evidence suggests that barriers such as lack of staff buy-in, resistance to change, and difficulties with implementation can affect the effectiveness of algorithms. There is a need for rigorous use of EB recommendations in the development of algorithms and overcoming these challenges with the use of implementation frameworks to facilitate algorithm quality and successful implementation aligned with the clinical setting.

## Data Availability

The original contributions presented in the study are included in the article/**Supplementary Material**, further inquiries can be directed to the corresponding author.
