# Supplementary material for: Effectiveness, quality and implementation of pain, sedation, delirium, and iatrogenic withdrawal syndrome algorithms in pediatric intensive care: a systematic review and meta-analysis
Source: Front Pediatr. 2023 Jun 16;11:1204622. doi: 10.3389/fped.2023.1204622 (PMC10313131; doi:10.3389/fped.2023.1204622)
Supplement: Supplementary file 1 [file Presentation1.pdf]

# *Supplementary Material*


**Table S1:** PRISMA checklist (1) ........ 3
**Table S2:** PRISMA-S checklist (2) ........ 7
**Table S3:** Search strategies ........ 9
**Table S4:** PROFILE structure and items ........ 14
**Table S5:** Study exclusion table with reasons ........ 15
**Table S6:** Characteristics of included studies ........ 24
**Table S7:** Characteristics of included algorithms ........ 43
**Table S8:** Measurement instruments and frequency of monitoring grouped by condition[*] ........ 52
**JBI Tables** ........ 53
  **Table S9:** Quality appraisal - JBI RCT (n=2) ........ 53
  **Table S10:** Quality appraisal - JBI Quasi-experimental (n=23) ........ 54
  **Table S11:** Quality appraisal - JBI Cohort (n=7) ........ 55
**Subgroup analysis by algorithm type** ........ 56
  **Figure S1**: Subgroup analysis - ICU length of stay ........ 56
  **Figure S2**: Subgroup analysis - Hospital length of stay ........ 57
  **Figure S3**: Subgroup analysis - Length of MV ........ 58
  **Figure S4**: Subgroup analysis: Duration of analgesic medications ........ 59
  **Figure S5**: Subgroup analysis: Duration of sedative medications ........ 60
  **Figure S6**: Subgroup analysis: Cumulative dose of analgesic medications ........ 61
  **Figure S7**: Subgroup analysis: Cumulative dose of sedative medications ........ 62
  **Figure S8**: Subgroup analysis: Incidents of iatrogenic withdrawal syndrome ........ 63
**Figure S9**: Funnel plots for continuous outcomes of interest ........ 64

**Sensitivity analysis (leave one study at a time out)** ... 66
**Figure S10:** Sensitivity analysis – intensive care unit LOS ... 66
**Figure S11:** Sensitivity analysis – hospital LOS ... 67
**Figure S12:** Sensitivity analysis – length of MV ... 68
**Figure S13**: Sensitivity analysis – Duration of analgesic medications ... 69
**Figure S14:** Sensitivity analysis – Duration of sedative medications ... 69
**Figure S15:** Sensitivity analysis – Cumulative dose of analgesic medications ... 70
**Figure S16:** Sensitivity analysis – Cumulative dose of sedative medications ... 71
**Figure S17:** Sensitivity analysis – Incidents of iatrogenic withdrawal syndrome ... 72
**Figure S18:** Sensitivity analysis – Incidents of under-sedation ... 72
**Figure S19:** Sensitivity analysis – Duration of weaning ... 73
**Figure S20:** Sensitivity analysis – Duration of methadone ... 73
**Table S12:** PROFILE: Items and Quality Scores ... 74
**Table S13:** EPOC strategies for algorithm implementation ... 75
**Table S14:** Barriers and facilitators of algorithm implementation ... 76
**Table S15:** Fidelity to algorithms and subcomponents ... 79
**Table S16:** Staff and parental satisfaction ... 80
**References** ... 81

43 **Table S1:** PRISMA checklist (1)

| Section and Topic | Item # | Checklist item | Location where item is reported: page#* |
|---|---|---|---|
| **TITLE** | | | |
| Title | 1 | Identify the report as a systematic review. | Abstract p1, p3 |
| **ABSTRACT** | | | |
| Abstract | 2 | See the PRISMA 2020 for Abstracts checklist. | P1 |
| **INTRODUCTION** | | | |
| Rationale | 3 | Describe the rationale for the review in the context of existing knowledge. | P2 |
| Objectives | 4 | Provide an explicit statement of the objective(s) or question(s) the review addresses. | P3 |
| **METHODS** | | | |
| Eligibility criteria | 5 | Specify the inclusion and exclusion criteria for the review and how studies were grouped for the syntheses. | P3 |
| Information sources | 6 | Specify all databases, registers, websites, organisations, reference lists and other sources searched or consulted to identify studies. Specify the date when each source was last searched or consulted. | P3 |
| Search strategy | 7 | Present the full search strategies for all databases, registers and websites, including any filters and limits used. | Supplementary material (P9-13) |
| Selection process | 8 | Specify the methods used to decide whether a study met the inclusion criteria of the review, including how many reviewers screened each record and each report retrieved, whether they worked independently, and if applicable, details of automation tools used in the process. | P3 |
| Data collection process | 9 | Specify the methods used to collect data from reports, including how many reviewers collected data from each report, whether they worked independently, any processes for obtaining or confirming data from study investigators, and if applicable, details of automation tools used in the process. | P4-6 |
| Data items | 10a | List and define all outcomes for which data were sought. Specify whether all results that were compatible with each outcome domain in each study were sought (e.g. for all measures, time points, analyses), and if not, the methods used to decide which results to collect. | P4-6 |
| | 10b | List and define all other variables for which data were sought (e.g. participant and intervention characteristics, funding sources). Describe any assumptions made about any missing or unclear information. | P4-6 |
| Study risk of bias assessment | 11 | Specify the methods used to assess risk of bias in the included studies, including details of the tool(s) used, how many reviewers assessed each study and whether they worked independently, and if applicable, details of automation tools used in the process. | P4 |
| Effect measures | 12 | Specify for each outcome the effect measure(s) (e.g. risk ratio, mean difference) used in the synthesis or presentation of results. | P4 |

| Section and Topic | Item # | Checklist item | Location where item is reported: page#* |
|---|---|---|---|
| Synthesis methods | 13a | Describe the processes used to decide which studies were eligible for each synthesis (e.g. tabulating the study intervention characteristics and comparing against the planned groups for each synthesis (item #5)). | P4-6 |
| | 13b | Describe any methods required to prepare the data for presentation or synthesis, such as handling of missing summary statistics, or data conversions. | P4-6 |
| | 13c | Describe any methods used to tabulate or visually display results of individual studies and syntheses. | P4-6 |
| | 13d | Describe any methods used to synthesize results and provide a rationale for the choice(s). If meta-analysis was performed, describe the model(s), method(s) to identify the presence and extent of statistical heterogeneity, and software package(s) used. | P4-6 |
| | 13e | Describe any methods used to explore possible causes of heterogeneity among study results (e.g. subgroup analysis, meta-regression). | P4 |
| | 13f | Describe any sensitivity analyses conducted to assess robustness of the synthesized results. | P4 |
| Reporting bias assessment | 14 | Describe any methods used to assess risk of bias due to missing results in a synthesis (arising from reporting biases). | P4 |
| Certainty assessment | 15 | Describe any methods used to assess certainty (or confidence) in the body of evidence for an outcome. | P4-5 |
| **RESULTS** | | | |
| Study selection | 16a | Describe the results of the search and selection process, from the number of records identified in the search to the number of studies included in the review, ideally using a flow diagram. | P6 (PRISMA figure 1) |
| | 16b | Cite studies that might appear to meet the inclusion criteria, but which were excluded, and explain why they were excluded. | Supplementary material P15-23 |
| Study characteristics | 17 | Cite each included study and present its characteristics. | P6 (supplementary material P24 - 42) |
| Risk of bias in studies | 18 | Present assessments of risk of bias for each included study. | P7 (Supplementary material P53-55) |
| Results of individual studies | 19 | For all outcomes, present, for each study: (a) summary statistics for each group (where appropriate) and (b) an effect estimate and its precision (e.g. confidence/credible interval), ideally using structured tables or plots. | Figures 1-6 |

| Section and Topic | Item # | Checklist item | Location where item is reported: page#* |
|---|---|---|---|
| Results of syntheses | 20a | For each synthesis, briefly summarise the characteristics and risk of bias among contributing studies. | P7-11 |
| | 20b | Present results of all statistical syntheses conducted. If meta-analysis was done, present for each the summary estimate and its precision (e.g. confidence/credible interval) and measures of statistical heterogeneity. If comparing groups, describe the direction of the effect. | P7-11 |
| | 20c | Present results of all investigations of possible causes of heterogeneity among study results. | Supplementary materials P56-69 |
| | 20d | Present results of all sensitivity analyses conducted to assess the robustness of the synthesized results. | P11 (Supplementary materials P66-73) |
| Reporting biases | 21 | Present assessments of risk of bias due to missing results (arising from reporting biases) for each synthesis assessed. | P7-11 |
| Certainty of evidence | 22 | Present assessments of certainty (or confidence) in the body of evidence for each outcome assessed. | P11<br>Table 2 |
| **DISCUSSION** | | | |
| Discussion | 23a | Provide a general interpretation of the results in the context of other evidence. | P13-15 |
| | 23b | Discuss any limitations of the evidence included in the review. | P15 |
| | 23c | Discuss any limitations of the review processes used. | P15 |
| | 23d | Discuss implications of the results for practice, policy, and future research. | P15-16 |
| **OTHER INFORMATION** | | | |
| Registration and protocol | 24a | Provide registration information for the review, including register name and registration number, or state that the review was not registered. | P1- Abstract ; P3 |
| | 24b | Indicate where the review protocol can be accessed, or state that a protocol was not prepared. | Abstract P1 ; P3 |
| | 24c | Describe and explain any amendments to information provided at registration or in the protocol. | P3 |
| Support | 25 | Describe sources of financial or non-financial support for the review, and the role of the funders or sponsors in the review. | P16 |
| Competing interests | 26 | Declare any competing interests of review authors. | P16 |

| Section and Topic | Item # | Checklist item | Location where item is reported: page#* |
|---|---|---|---|
| Availability of data, code and other materials | 27 | Report which of the following are publicly available and where they can be found: template data collection forms; data extracted from included studies; data used for all analyses; analytic code; any other materials used in the review. | N/A |

*based on pages being numbers 1-19

**Table S2:** PRISMA-S checklist (2)

| Section/topic | # | Checklist item | Location(s) Reported (page#)* |
|---|---|---|---|
| **INFORMATION SOURCES AND METHODS** | | | |
| Database name | 1 | Name each individual database searched, stating the platform for each. | P3 |
| Multi-database searching | 2 | If databases were searched simultaneously on a single platform, state the name of the platform, listing all of the databases searched. | Not applicable |
| Study registries | 3 | List any study registries searched. | Not applicable |
| Online resources and browsing | 4 | Describe any online or print source purposefully searched or browsed (e.g., tables of contents, print conference proceedings, web sites), and how this was done. | P3 |
| Citation searching | 5 | Indicate whether cited references or citing references were examined, and describe any methods used for locating cited/citing references (e.g., browsing reference lists, using a citation index, setting up email alerts for references citing included studies). | P3 |
| Contacts | 6 | Indicate whether additional studies or data were sought by contacting authors, experts, manufacturers, or others. | P5 |
| Other methods | 7 | Describe any additional information sources or search methods used. | P3 |
| **SEARCH STRATEGIES** | | | |
| Full search strategies | 8 | Include the search strategies for each database and information source, copied and pasted exactly as run. | Supplementary material P9-13 |
| Limits and restrictions | 9 | Specify that no limits were used, or describe any limits or restrictions applied to a search (e.g., date or time period, language, study design) and provide justification for their use. | P3 |
| Search filters | 10 | Indicate whether published search filters were used (as originally designed or modified), and if so, cite the filter(s) used. | |

| | | | |
|---|---|---|---|
| Prior work | 11 | Indicate when search strategies from other literature reviews were adapted or reused for a substantive part or all of the search, citing the previous review(s). | Not applicable and indicated on P3 |
| Updates | 12 | Report the methods used to update the search(es) (e.g., rerunning searches, email alerts). | P3 (supplementary material P9) |
| Dates of searches | 13 | For each search strategy, provide the date when the last search occurred. | P3 (supplementary material P9-13) |
| **PEER REVIEW** | | | |
| Peer review | 14 | Describe any search peer review process. | P3 |
| **MANAGING RECORDS** | | | |
| Total Records | 15 | Document the total number of records identified from each database and other information sources. | PRISMA flow diagram (Figure 1)(Supplementary materials P9-13) |
| Deduplication | 16 | Describe the processes and any software used to deduplicate records from multiple database searches and other information sources. | P3 |

*based on pages being numbers 1-19

**Table S3:** Search strategies

The first search (September 29, 2020) limited publication languages to French and English and publication dates to 2000-current for all databases. When updating the search on December 7, 2021, the language limit was removed, and the date limit was changed to 2005-current. Before re-running the searches on December 7, 2021, and November 29, 2022, all search strategies' syntax and index terms were verified by AT.

| Database | Final search strategy (November 29, 2022) | Results |
|---|---|---|
| PubMed.gov | ("Pain"[Mesh] OR "Analgesia"[Mesh] OR "Pain Management"[Mesh] OR "Conscious Sedation"[Mesh] OR "Hypnotics and Sedatives"[Mesh:NoExp] OR "Confusion"[Mesh] OR "Psychomotor Agitation"[Mesh] OR "Substance Withdrawal Syndrome"[Mesh:NoExp] OR Pain[tiab] OR Discomfort[tiab] OR Distress[tiab] OR Agony[tiab] OR Suffer*[tiab] OR Hurt*[tiab] OR Throb*[tiab] OR Sore*[tiab] OR Analgesia[tiab] OR Sedation[tiab] OR Sedative*[tiab] OR Conscious*[tiab] OR Confusion[tiab] OR Delirium[tiab] OR agitation[tiab] OR delirious*[tiab] OR Inattentiveness[tiab] OR Disorientation[tiab] OR Withdrawal*[tiab] OR "abstinence symptom*"[tiab] OR "abstinence syndrom*"[tiab] OR "cessation symptom*"[tiab] OR "cessation syndrom*"[tiab] OR "discontinuation symptom*"[tiab] OR "discontinuation syndrom*"[tiab] OR weaning[tiab]) AND ("Intensive Care Units, Pediatric"[Mesh] OR "Intensive Care, Neonatal"[Mesh] OR "Pediatric Intensive Care"[tiab] OR "Paediatric Intensive Care"[tiab] OR "Pediatric ICU*"[tiab] OR "Paediatric ICU*"[tiab] OR PICU*[tiab] OR "newborn intensive care"[tiab] OR "newborn ICU*"[tiab] OR "neonatal intensive care"[tiab] OR "neonatal ICU*"[tiab] OR NICU*[tiab] OR "Pediatric Critical Care"[tiab] OR "Paediatric Critical Care"[tiab] OR (("Intensive Care Units"[Mesh:NoExp] OR "Critical Care"[Mesh] OR "Critical Care Nursing"[Mesh] OR "Intensive Care"[tiab] OR "Critical Care Unit*"[tiab] OR "Critical Care Setting*"[tiab] OR ICU[tiab] OR ICUs[tiab] OR GICU*[tiab] OR "Intensive Therapy Unit*"[tiab] OR "Intensive Treatment Unit*"[tiab]) AND ("Adolescent"[Mesh] OR "Child"[Mesh] OR "Pediatrics"[Mesh] OR "Infant"[Mesh] OR prepube*[tiab] OR preadolescen*[tiab] OR adolescen*[tiab] OR teenager*[tiab] OR youth[tiab] OR juvenile[tiab] OR child*[tiab] OR kid[tiab] OR kids[tiab] OR baby[tiab] OR babies[tiab] OR infant[tiab] OR infants[tiab] OR neonat*[tiab] OR newborn*[tiab] OR pediatric[tiab] OR pediatrics[tiab] OR paediatric[tiab] OR paediatrics[tiab] OR prematur*[tiab]))) AND ("Algorithms"[Mesh] OR "Decision Trees"[Mesh] OR "Checklist"[Mesh] OR "Critical Pathways"[Mesh] OR "Clinical Protocols"[Mesh] OR "Practice Guidelines as Topic"[Mesh] | First search September 29, 2020, 1383 records<br><br>Second search December 7, 2021, 1578 records of which 308 were new<br><br>Third search November 29, 2022, 1765 records of which 176 were new |

| Database | Final search strategy (November 29, 2022) | Results |
|---|---|---|
| | OR "Practice Guideline"[Publication Type] OR "Guideline Adherence"[Mesh] OR "Patient Care Bundles"[Mesh] OR Algorithm*[tiab] OR "Clinical pathway*"[tiab] OR guideline*[tiab] OR "Decision tree*"[tiab] OR Checklist*[tiab] OR Protocol*[tiab]) AND (2005[dp] : 3000[dp]) | |
| Embase.com | ('pain'/exp OR 'analgesia'/exp OR 'conscious sedation'/de OR 'hypnotic sedative agent'/de OR 'confusion'/exp OR 'restlessness'/de OR 'drug-induced akathisia'/de OR 'delirium'/de OR 'emergence agitation'/de OR 'hyperactive delirium'/de OR 'hypoactive delirium'/de OR 'withdrawal syndrome'/de OR (Pain OR Discomfort OR Distress OR Agony OR Suffer* OR Hurt* OR Throb* OR Sore* OR Analgesia OR Sedation OR Sedative* OR Conscious* OR Confusion OR Delirium OR Agitation OR delirious* OR Inattentiveness OR Disorientation OR Withdrawal* OR "abstinence symptom*" OR "abstinence syndrom*" OR "cessation symptom*" OR "cessation syndrom*" OR "discontinuation symptom*" OR "discontinuation syndrom*" OR weaning):ab,ti,kw) AND ('pediatric intensive care unit'/de OR 'pediatric intensive care nursing'/de OR 'neonatal intensive care unit'/de OR 'newborn intensive care nursing'/de OR 'newborn intensive care'/de OR ("Pediatric Intensive Care" OR "Paediatric Intensive Care" OR "Pediatric ICU*" OR "Paediatric ICU*" OR PICU* OR "newborn intensive care" OR "newborn ICU*" OR "neonatal intensive care" OR "neonatal ICU*" OR NICU* OR "Pediatric Critical Care" OR "Paediatric Critical Care"):ab,ti,kw OR (('intensive care unit'/de OR 'intensive care'/de OR 'intensive care nursing'/de OR ("Intensive Care" OR ICU* OR "Critical Care Unit*" OR "Critical Care Setting*" OR GICU* OR "Intensive Therapy Unit*" OR "Intensive Treatment Unit*"):ab,ti,kw) AND ('adolescent'/exp OR 'child'/exp OR 'pediatrics'/de OR 'neonatology'/exp OR (prepube* OR preadolescen* OR adolescen* OR teenager* OR youth OR juvenile OR child* OR kid OR kids OR baby OR babies OR infant OR infants OR neonat* OR newborn* OR pediatric OR pediatrics OR paediatric OR paediatrics OR prematur*):ab,ti,kw))) AND ('algorithm'/exp OR 'decision tree'/de OR 'checklist'/de OR 'clinical pathway'/de OR 'clinical protocol'/de OR 'nursing protocol'/de OR 'practice guideline'/de OR 'protocol compliance'/de OR 'care bundle'/de OR (Algorithm* OR "Clinical pathway*" OR guideline* OR "Decision tree*" OR Checklist* OR Protocol*):ab,ti,kw) AND [2005-3000]/py NOT (('conference abstract'/it OR 'conference review'/it) AND [2005-2015]/py) | First search September 29, 2020, 2274 records<br><br>Second search December 7, 2021, 2772 records of which 415 were new<br>Third search November 29, 2022, 3253 records of which 309 were new |

| Database | Final search strategy (November 29, 2022) | Results |
|---|---|---|
| CINAHL with Full Text (EBSCO) | ((MH "Pain+") OR (MH "Analgesia+") OR (MH "Pain Management") OR (MH "Conscious Sedation") OR (MH "Hypnotics and Sedatives") OR (MH "Confusion+") OR (MH "Psychomotor Agitation+") OR (MH "Substance Withdrawal Syndrome") OR TI(Pain OR Discomfort OR Distress OR Agony OR Suffer* OR Hurt* OR Throb* OR Sore* OR Analgesia OR Sedation OR Sedative* OR Conscious* OR Confusion OR Delirium OR Agitation OR delirious* OR Inattentiveness OR Disorientation OR Withdrawal* OR "abstinence symptom*" OR "abstinence syndrom*" OR "cessation symptom*" OR "cessation syndrom*" OR "discontinuation symptom*" OR "discontinuation syndrom*" OR weaning) OR AB(Pain OR Discomfort OR Distress OR Agony OR Suffer* OR Hurt* OR Throb* OR Sore* OR Analgesia OR Sedation OR Sedative* OR Conscious* OR Confusion OR Delirium OR Agitation OR delirious* OR Inattentiveness OR Disorientation OR Withdrawal* OR "abstinence symptom*" OR "abstinence syndrom*" OR "cessation symptom*" OR "cessation syndrom*" OR "discontinuation symptom*" OR "discontinuation syndrom*" OR weaning)) AND ((MH "Intensive Care Units, Pediatric+") OR (MH "Pediatric Critical Care Nursing+") OR (MH "Intensive Care, Neonatal") OR (MH "Pediatric Critical Care Nursing+") OR TI("Pediatric Intensive Care" OR "Paediatric Intensive Care" OR "Pediatric ICU*" OR "Paediatric ICU*" OR PICU* OR "newborn intensive care" OR "newborn ICU*" OR "neonatal intensive care" OR "neonatal ICU*" OR NICU* OR "Pediatric Critical Care" OR "Paediatric Critical Care") OR AB("Pediatric Intensive Care" OR "Paediatric Intensive Care" OR "Pediatric ICU*" OR "Paediatric ICU*" OR PICU* OR "newborn intensive care" OR "newborn ICU*" OR "neonatal intensive care" OR "neonatal ICU*" OR NICU* OR "Pediatric Critical Care" OR "Paediatric Critical Care") OR (((MH "Intensive Care Units") OR (MH "Critical Care") OR (MH "Critical Care Nursing") OR TI("Intensive Care" OR "Critical Care Unit*" OR "Critical Care Setting*" OR ICU* OR GICU* OR "Intensive Therapy Unit*" OR "Intensive Treatment Unit*") OR AB("Intensive Care" OR "Critical Care Unit*" OR "Critical Care Setting*" OR ICU* OR GICU* OR "Intensive Therapy Unit*" OR "Intensive Treatment Unit*")) AND ((MH "Adolescence+") OR (MH "Child+") OR (MH "Pediatrics+") OR TI(prepube* OR preadolescen* OR adolescen* OR teenager* OR youth OR juvenile OR child* OR kid OR kids OR baby OR babies OR infant OR infants OR neonat* OR newborn* OR pediatric OR pediatrics OR paediatric OR paediatrics OR prematur*) OR AB(prepube* OR preadolescen* OR adolescen* OR teenager* OR youth OR juvenile OR child* OR kid OR kids OR baby OR babies OR infant OR infants OR neonat* OR newborn* OR pediatric OR pediatrics OR | First search September 29, 2020, 745 records<br><br>Second search December 7, 2021, 863 records found of which 57 were new<br><br>Third search November 29, 2022, 959 records of which 30 were new |

| Database | Final search strategy (November 29, 2022) | Results |
|---|---|---|
| | paediatric OR paediatrics OR prematur*)))) AND ((MH "Algorithms") OR (MH "Decision Trees+") OR (MH "Checklists") OR (MH "Critical Path") OR (MH "Nursing Protocols") OR (MH "Practice Guidelines") OR (MH "Guideline Adherence") OR TI(Algorithm* OR "Clinical pathway*" OR guideline* OR "Decision tree*" OR Checklist* OR Protocol*) OR AB(Algorithm* OR "Clinical pathway*" OR guideline* OR "Decision tree*" OR Checklist* OR Protocol*)) AND PY 2005-3000 | |
| Cochrane Library Wiley (Cochrane Database of Systematic Reviews and Cochrane Central Register of Controlled Trials (CENTRAL)) | (Pain OR Discomfort OR Distress OR Agony OR Suffer* OR Hurt* OR Throb* OR Sore* OR Analgesia OR Sedation OR Sedative* OR Conscious* OR Confusion OR Delirium OR Agitation OR delirious* OR Inattentiveness OR Disorientation OR Withdrawal* OR (abstinence NEXT symptom*) OR (abstinence NEXT syndrom*) OR (cessation NEXT symptom*) OR (cessation NEXT syndrom*) OR (discontinuation NEXT symptom*) OR (discontinuation NEXT syndrom*) OR weaning):ab,ti,kw AND ("Pediatric Intensive Care" OR "Paediatric Intensive Care" OR (Pediatric NEXT ICU*) OR (Paediatric NEXT ICU*) OR PICU* OR "newborn intensive care" OR (newborn NEXT ICU*) OR "neonatal intensive care" OR (neonatal NEXT ICU*) OR NICU* OR "Pediatric Critical Care" OR "Paediatric Critical Care" OR (("Intensive Care" OR ICU* OR (Critical NEXT Care NEXT Unit*) OR (Critical NEXT Care NEXT Setting*) OR GICU* OR (Intensive NEXT Therapy NEXT Unit*) OR (Intensive NEXT Treatment NEXT Unit*)) AND (prepube* OR preadolescen* OR adolescen* OR teenager* OR youth OR juvenile OR child* OR kid OR kids OR baby OR babies OR infant OR infants OR neonat* OR newborn* OR pediatric OR pediatrics OR paediatric OR paediatrics OR prematur*))):ab,ti,kw AND (Algorithm* OR (Clinical NEXT pathway*) OR guideline* OR (Decision NEXT tree*) OR Checklist* OR Protocol*):ab,ti,kw<br><br>Publication date added manually:<br>Cochrane Reviews 01/01/2005 to 31/12/2022<br>Cochrane Protocols 01/01/2005 to 31/12/2022<br>CENTRAL Custom Range 2005 to 2022 | First search September 29, 2020, 536 records<br><br>Second search December 7, 2021, 662 records found of which 107 were new<br><br>Third search November 29, 2022, 739 records of which 68 were new |
| ProQuest Dissertations & Theses Global (ProQuest) | ALL((Pain OR Discomfort OR Distress OR Agony OR Suffer* OR Hurt* OR Throb* OR Sore* OR Analgesia OR Sedation OR Sedative* OR Conscious* OR Confusion OR Delirium OR Agitation OR delirious* OR Inattentiveness OR Disorientation OR Withdrawal* OR "abstinence symptom*" OR "abstinence syndrom*" OR "cessation symptom*" OR "cessation syndrom*" OR "discontinuation symptom*" OR "discontinuation syndrom*" OR weaning) | First search September 29, 2020, 41 records |

| Database | Final search strategy (November 29, 2022) | Results |
|---|---|---|
| | AND ("Pediatric Intensive Care" OR "Paediatric Intensive Care" OR "Pediatric ICU*" OR "Paediatric ICU*" OR PICU* OR "newborn intensive care" OR "newborn ICU*" OR "neonatal intensive care" OR "neonatal ICU*" OR NICU* OR "Pediatric Critical Care" OR "Paediatric Critical Care" OR (("Intensive Care" OR ICU* OR "Critical Care Unit*" OR "Critical Care Setting*" OR GICU* OR "Intensive Therapy Unit*" OR "Intensive Treatment Unit*") AND (prepube* OR preadolescen* OR adolescen* OR teenager* OR youth OR juvenile OR child* OR kid OR kids OR baby OR babies OR infant OR infants OR neonat* OR newborn* OR pediatric OR pediatrics OR paediatric OR paediatrics OR prematur*))) AND (Algorithm* OR "Clinical pathway*" OR guideline* OR "Decision tree*" OR Checklist* OR Protocol*)) AND YR(2005-3000) | Second search December 7, 2021, 52 records of which 15 were new<br><br>Third search November 29, 2022, 63 records of which 10 were new records |
| Google Scholar | Pain\|Sedation\|Delirium\|Withdrawal\|"abstinence\|cessation\|discontinuation symptom\|syndrom"\|weaning "Pediatric\|paediatric\|neonatal\|newborn Intensive Care" Algorithm\|"Clinical pathway"\|guideline\|"Decision tree"\|Checklist\|Protocol | First search November 7, 2020. First 300 records screened and 8 found<br><br>Second search January 7, 2022. First 300 records screened and 4 found<br><br>Third search November 30, 2022. First 300 records screened and 3 found |

**Table S4:** PROFILE structure and items

| Domains | Number | Items | Processes |
|---|---|---|---|
| **1: Scope and purpose** | 3 | 1. Algorithm objectives described (e.g. health intent, benefits, condition/clinical problem)<br>2. Algorithm management is interprofessional<br>3. The population (patients) for whom the algorithm applies are described | Development |
| **2: Stakeholder involvement** | 3 | 4. Algorithm development group is interprofessional<br>5. The views and preferences of staff on the unit were sought<br>6. The views and preferences of patients/families were sought | |
| **3: Rigor of development** | 9 | 7. Systematic methods were used to search for evidence<br>8. The evidence quality was evaluated<br>9. The highest level of evidence was used whenever available (e.g. clinical practice guidelines)<br>10. The strengths and limitations of the evidence were described<br>11. The process for developing the algorithm are described<br>12. The process for finalizing the algorithm is described (consensus, endorsed, piloted)<br>13. The health benefits, side effects, and risks are described as part of the decision-making process during the development of the algorithm<br>14. Parts of the algorithm are supported/linked to the evidence<br>15. A process for periodically reviewing the algorithm is described | |
| **4: Content, structure, documentation and roles** | 5 | 16. The steps/flow of the algorithm are easy to follow<br>17. The different options for management of the condition/health issue are presented<br>18. Information on documentation is provided<br>19. Roles and responsibilities of each team member are described<br>20. There are targets and timeframes included in the algorithm | Content |
| **5: Implementation** | 4 | 21. A need for the algorithm was identified<br>22. Staff participated in identifying barriers and facilitators towards algorithm implementation and these were used to supported implementation (i.e. PDSA cycles, focus groups)<br>23. Monitoring criteria were developed<br>24. At least two EPOC implementation strategies were used (must include education) | Implementation |

**Table S5:** Study exclusion table with reasons

| **Reference** | **Reason for exclusion** |
|---|---|
| Achuff BJ, Lemming K, Causey JC, Sembera KA, Checchia PA, Heinle JS, et al. Opioid Weaning Protocol Using Morphine Compared With Nonprotocolized Methadone Associated With Decreased Dose and Duration of Opioid After Norwood Procedure. Pediatr Crit Care Med. 2022;23(5):361-70. | 3b |
| Aikman, N., Kettle, R., Leonard, G., Whitby, T., Lant, A., & Yajamanyam, K. (2016). Management of Pain in Preterm Infants Undergoing Painful Procedures - Experience in a Uk Neonatal Intensive Care Unit. *European Journal of Pediatrics, 175*(11), 1823-1823. | 1a |
| Allen, R. (2022). Evaluation of a process change using an updated sedation protocol (Order No. 29319763). Available from ProQuest Dissertations & Theses Global. (2702448167). Retrieved from https://www.proquest.com/dissertations-theses/evaluation-process-change-using-updated-sedation/docview/2702448167/se-2 | 4a |
| Amigoni, A., Catalano, I., Vettore, E., Brugnaro, L., & Pettenazzo, A. (2012). Practice of analgesia and sedation in Italian Paediatric Intensive Care Units: did we progress? *Minerva Anestesiol, 78*(12), 1365-1371. | 3a |
| Angus, L., MacKay, M., Coughlin, M., DeWolfe, T., Fuller, K., & Bauwens, N. (2022). A pain and stress assessment and management quality improvement initiative to align with the BEST START recommendations. Journal of Neonatal Nursing | 4a |
| Aukes, D. I., Roofthooft, D. W. E., Simons, S. H. P., Tibboel, D., & van Dijk, M. (2015). Pain Management in Neonatal Intensive Care: Evaluation of the Compliance With Guidelines. *31*(9), 830-835. doi:10.1097/ajp.0000000000000168 | 4c |
| Barker, A., Spence, K., & Halliday, R. (2015). Can a reduction in ventilation days be maintained using a weaning protocol in the nicu? *Journal of paediatrics and child health., 51*, 87. | 1° |
| Berger, M. M., Davadant, M., Marin, C., Wasserfallen, J.-B., Pinget, C., Maravic, P., . . . Chiolero, R. L. (2010). Impact of a pain protocol including hypnosis in major burns. Burns, 36(5), 639-646. Doi:https://doi.org/10.1016/j.burns.2009.08.009 | 2 |
| Blackwood, Bronagh, et al. (2021). Effect of a sedation and ventilator liberation protocol vs usual care on duration of invasive mechanical ventilation in pediatric intensive care units: a randomized clinical trial. *JAMA,* 326(5), 401-410. | 4a |
| Blackwood, B., Morris, K. P., Jordan, J., McIlmurray, L., Agus, A., Boyle, R., ... & McAuley, D. F. (2022). Co-ordinated multidisciplinary intervention to reduce time to successful extubation for children on mechanical ventilation: the SANDWICH cluster stepped-wedge RCT. Health Technology Assessment, 26(18), 1-114 | 4a |

| Reference | Reason for exclusion |
|---|---|
| Borges, L. G. A., Savi, A., Teixeira, C., de Oliveira, R. P., De Camillis, M. L. F., Wickert, R., . . . Vieira, S. R. R. (2017). Mechanical ventilation weaning protocol improves medical adherence and results. *J Crit Care, 41*, 296-302. doi:10.1016/j.jcrc.2017.07.014. Epub 2017 Jul 12. | 2 |
| Brahmbhatt K. 9.1 Implementing Delirium Screening and Management Clinical Pathway in a Pediatric Intensive Care Unit. Journal of the American Academy of Child & Adolescent Psychiatry. 2022;61(10):S14. | 1a |
| Ceelie, I., de Wildt, S. N., de Jong, M., Ista, E., Tibboel, D., & van Dijk, M. (2012). Protocolized post-operative pain management in infants; do we stick to it? European Journal of Pain, 16(5), 760-766. doi:https://doi.org/10.1002/j.1532-2149.2011.00056.x | 4c |
| Chan, S. Y. (2021). Development, implementation, and evaluation of a clinical practice guideline for care of preterm infants receiving non-invasive ventilation: A before and after study (Order No. 29186276). Available from ProQuest Dissertations & Theses Global. (2655619210). Retrieved from https://www.proquest.com/dissertations-theses/development-implementation-evaluation-clinical/docview/2655619210/se-2 | 4a |
| Chan, J., Goldstein, M., & Maka, D. (2021). 1124: Utilizing a Standardized Pain and Agitation Protocol for Intubated Patients in the Pediatric ICU. Critical Care Medicine, 49(1), 563. | 1a |
| Chavananon, S., & Ruangnapa, K. (2016). Effectiveness of protocolized sedation with the comfort b scale in mechanically ventilated children. *Cogent Medicine, 3*(1). | 1a |
| Chloe, A., Cesar, R., & Fabrice M. (2022). Sedation-analgesia in pediatric ICU: PEDIASLEEP study. [Abstract] In proceedings of Reanimation 2022, the French Intensive Care Society International Congress. Ann. Intensive Care 12 (Suppl 1), 54 (2022). https://doi.org/10.1186/s13613-022-01016-6 | 1a |
| Cloedt LD, Benbouzid K, Lavoie A, Metras ME, Lavoie MC, Harakat S, et al. The Impact of Implementing a "Pain, Agitation, and Delirium Bundle" in a Pediatric Intensive Care Unit: Improved Delirium Diagnosis. Journal of Pediatric Intensive Care. 2021(EFirst). Epub 11.02.2021. doi: 10.1055/s-0041-1723037. PubMed PMID: WOS:000617034300002. | 4a |
| Cobb EB. Implementation of an Extubation Readiness Guideline for Preterm Infants: University of Maryland Baltimore; 2022 | 4b |
| Ctri. protocolized sedation vs non-protocolized sedation in mechanically ventilated patients. https://trialsearchwhoint/Trial2aspx?TrialID=CTRI/2021/04/033130. 2021. PubMed PMID: rayyan-244488237. | 1b |
| David, L., Forest, S., & Harris-Haman, P. A. (2022). Development and Implementation of a Neonatal Pain Management Guideline for Minor Surgeries. *Advances in Neonatal Care*, *22*(5), 391-399. | 3b |

| Reference | Reason for exclusion |
| --- | --- |
| Donnellan, A., Sawyer, J., Peach, A., Staveski, S., Nelson, D. P., & Pratap, J. N. (2019). Reducing exposure to opioid and benzodiazepine medications for pediatric cardiac intensive vare patients: A quality improvement project. Pediatric critical care medicine, 20(4), 340-349. | 3b |
| Dreyfus, L., Bordet, F., Touzet, S., Denis, A., & Javouhey, E. (2016). Implementation and evaluation of a paediatric nurse-driven sedation protocol in a PICU. *Annals of intensive care, 6*. | 1a |
| Duke, M., McCracken, C., Wetzel, M., & DeAlmeida, M. (2019). 382: Effect of analgosedation protocol and sedative use on outcomes in pediatric intensive care unit. *Critical Care Medicine, 47*(1). | 1a |
| Duyndam, A., Houmes, R. J., Tibboel, D., & Ista, E. (2016). Effects of a nurse-driven ventilation weaning protocol on the PICU. *European Journal of Pediatrics, 175*(11), 1472-1473. | 1a |
| Duyndam, A., Houmes, R. J., van Rosmalen, J., Tibboel, D., van Dijk, M., & Ista, E. (2020). Implementation of a nurse-driven ventilation weaning protocol in critically ill children: Can it improve patient outcome? *Aust Crit Care, 33*(1), 80-88. doi:10.1016/j.aucc.2019.01.005. Epub 2019 Mar 13. | 4a |
| Elella, R. A., Adalaty, H., Koay, Y. N., Mokrusova, P., Theresa, M., Male, B., . . . Al Wadai, A. (2015). The efficacy of the COMFORT score and pain management protocol in ventilated pediatric patients following cardiac surgery. *International Journal of Pediatrics Adolescent Medicine, 2*(3-4), 123-127. | 4b |
| Elyas M, Haggag S, Atef A1306 Implementation of withdrawal assessment tool and weaning protocol to reduce iatrogenic withdrawal syndrome in pediatric cardiac ICU: a quality improvement projectArchives of Disease in Childhood 2022;107:A486-A488. | 1a |
| Erickson, S. J., Millar, J., Anderson, B. J., Festa, M. S., Straney, L., Shehabi, Y., & Long, D. A. (2020). Dexmedetomidine sedation in mechanically ventilated critically ill children: a pilot randomized controlled trial. Pediatric Critical Care Medicine, 21(9), e731-e739. | 3b |
| Eriksson M, Campbell-Yeo M. Assessment of pain in newborn infants. Semin Fetal Neonatal Med. 2019;24(4):101003. Epub 2019/04/17. doi: 10.1016/j.siny.2019.04.003. PubMed PMID: 30987943. | 4a |
| Espinoza, Elizabeth1; Steuber, Hailey2; Levett, Paula3. 1311: IMPACT OF SEDATION TITRATION GUIDELINES ON NURSING SATISFACTION AND SEDATION EXPOSURE IN THE PICU. Critical Care Medicine 50(1):p 656, January 2022. \| DOI: 10.1097/01.ccm.0000811568.08153.80 | 1a |
| Fleishman R, Zhou C, Gleason C, Larison C, Myaing M, Mangione-Smith R. Standardizing morphine use for ventilated preterm neonates with a nursing-driven comfort protocol. Journal of Perinatology. 2015;35(1):46-51 | 4c |
| Frank, Deborah; Kragie, Jennifer; Frank, Nicole; Sacco, Melissa; Lunsford, Kelly. 1296: REFINING SEDATION PRACTICES IN A PEDIATRIC ICU REQUIRES SUSTAINED EFFORT. Critical Care Medicine 50(1):p 649, January 2022. \| DOI: 10.1097/01.ccm.0000811508.13259.32 | 1a |

frontiers

| Reference | Reason for exclusion |
|---|---|
| Frankel WC, Maul TM, Chrysostomou C, Wearden PD, Lowry AW, Baker KN, et al. A Minimal Opioid Postoperative Management Protocol in Congenital Cardiac Surgery: Safe and Effective. Semin Thorac Cardiovasc Surg. 2020 | 3b |
| Franken A, Sebbens D, Mensik J. Pediatric delirium: Early identification of barriers to optimize success of screening and prevention. Journal of Pediatric Health Care. 2019;33(3):228-33 | 4a (1) |
| Garten L, Demirakca S, Harth I, Huth R, Kumpf M, Schindler M, et al. [Analgesia, sedation and management of delir in children and neonates]. Anasthesiol Intensivmed Notfallmed Schmerzther. 2015;50(11):712-21; | 5 |
| Grabski DF, Vavolizza RD, Lepore S, Levin D, Rasmussen SK, Swanson JR, et al. A Quality Improvement Intervention to Reduce Postoperative Opiate Use in Neonates. Pediatrics. 2020;146(6). | 4a |
| Habich, M., Wilson, D., Thielk, D., Melles, G. L., Crumlett, H. S., Masterton, J., & McGuire, J. (2012). Evaluating the effectiveness of pediatric pain management guidelines. *Journal of pediatric nursing, 27*(4), 336-345. doi:10.1016/j.pedn.2011.06.002 | 4a |
| Holland, C. L. (2005). *Development of a clinical practice guideline for managing sedation in intubated patients in the pediatric intensive care unit.* University of Manitoba (Canada), Ann Arbor. | 3b |
| Irct20210308050636N. (2021). Detrmining the effect of applying pain management protocol on nurses' clinical performance. https://trialsearchwhoint/Trial2aspx?TrialID=IRCT20210308050636N1. | 1b |
| Karmarkar, M., Moyer, L., Speziale, M., & Jenkins, W. (2021, January). IMPLEMENTING SCREENING FOR NEONATAL DELIRIUM IN THE NICU AT RADY CHILDREN'S HOSPITAL. In JOURNAL OF INVESTIGATIVE MEDICINE (Vol. 69, No. 1, pp. 282-282). BRITISH MED ASSOC HOUSE, TAVISTOCK SQUARE, LONDON WC1H 9JR, ENGLAND: BMJ PUBLISHING GROUP | 1a |
| Kawai, Y., Weatherhead, J. R., Traube, C., Owens, T. A., Shaw, B. E., Fraser, E. J., . . . Niedner, M. F. (2019). Quality Improvement Initiative to Reduce Pediatric Intensive Care Unit Noise Pollution With the Use of a Pediatric Delirium Bundle. *J Intensive Care Med, 34*(5), 383-390. doi:10.1177/0885066617728030. Epub 2017 Sep 1. | 4a |
| King, K., Holmes, S., Comery, K., Dugdale, C., Harrison, S., McCarthy, K., & Weavers, A. (2016). Pain and sedation in paediatric intensive care: A bundle of care approach. *Anaesthesia and Intensive Care, 44*(2), 321-322. | 1a |
| Kishk, O. A., Simone, S., Lardieri, A. B., Graciano, A. L., Tumulty, J., & Edwards, S. (2019). Antipsychotic Treatment of Delirium in Critically Ill Children: A Retrospective Matched Cohort Study. *J Pediatr Pharmacol Ther, 24*(3), 204-213. doi:10.5863/1551-6776-24.3.204. | 3b |
| Kongkiattikul, L., Dagenais, M., Ruo, N., Di Genova, T., Fontela, P., & Zavalkoff, S. (2018). A quality improvement project to standardize pain, agitation, and withdrawal assessments reduces usage of morphine and midazolam in the pediatric intensive care unit. *Pediatric critical care medicine, 19*(6), 257. | 1a |

| **Reference** | **Reason for exclusion** |
|---|---|
| Kongkiattikul, L., Dagenais, M., Ruo, N., Fontela, P., Di Genova, T., & Zavalkoff, S. (2019). The impact of a quality improvement project to standardize pain, agitation, and withdrawal assessments on the use of morphine and midazolam in the Pediatric Intensive Care Unit. *Paediatr Anaesth, 29*(4), 322-330. doi:10.1111/pan.13591. Epub 2019 Feb 22. | 4a |
| Lampin, M. E., Cavrois, C., Rousseaux, J., Lereun, C., & Leteurtre, S. (2019). Proceedings of Reanimation 2019, the French Intensive Care Society International Congress. *Ann Intensive Care, 9*(Suppl 1), 1-153. doi:10.1186/s13613-018-0474-7 | 1a |
| Larson, G. E., Arnup, S. J., Clifford, M., & Evans, J. (2013). How does the introduction of a pain and sedation management guideline in the paediatric intensive care impact on clinical practice? A comparison of audits pre and post guideline introduction. *Aust Crit Care, 26*(3), 118-123. doi:10.1016/j.aucc.2013.04.001. Epub 2013 May 14. | 4a |
| Larson, G., & McKeever, S. (2015). Introduction of a pain and sedation protocol can change paediatric intensive care practice. *Australian Critical Care, 28*(1), 51-51. | 1a |
| Laures, E. L., Bruene, D., Fayram, L. R., Houston, A., Kephart, K., Merrifield, E., & Vitale, S. (2021). Pediatric Pain Assessment in the Intensive Care Unit: An Evidence-Based Algorithm. *Pain Management Nursing*, *22*(3), 260-267. | 4c |
| Laures, E. L. (2021). Pain assessment in the pediatric intensive care unit (Order No. 28862823). Available from ProQuest Dissertations & Theses Global. (2633844524). Retrieved from https://www.proquest.com/dissertations-theses/pain-assessment-pediatric-intensive-care-unit/docview/2633844524/se-2 | 4c |
| Lavigne-Sims, S., Iheagwara, K., Mathews, R., Walker, G., & McMahon, P. (2019). Improving sedation levels: A quality improvement project. Journal of Investigative Medicine, 67(2), 626. doi:10.1136/jim-2018-000974.694 | 1a |
| Maloney, C. G. (2007). *Computerized weaning of childhood respiratory failure.* The University of Utah, Ann Arbor. | 6 – unable to retrieve |
| Matlock, D. N., & Ross, A. S. (2018). Neonatal non-invasive respiratory weaning protocol implementation. Journal of Investigative Medicine, 66(2), 522. doi:10.1136/jim-2017-000697.420 | 1a |
| Mazars, N., Milési, C., Carbajal, R., Mesnage, R., Combes, C., Novais, A. R. B., & Cambonie, G. (2012). Implementation of a neonatal pain management module in the computerized physician order entry system. Annals of intensive care, 2(1), 38. | 3b |
| Michel, J., Hofbeck, M., Gerbig, I., Icheva, V., Heimberg, E., Jost, W., . . . Neunhoeffer, F. (2017). Nurse-driven analgesia and sedation in pediatric patients with univentricular hearts requiring extracorporeal life support after first-stage palliation surgery: A pilot study. *Pediatric Anesthesia, 27*(12), 1261-1270. | 3b |

| Reference | Reason for exclusion |
| --- | --- |
| Michel, J., Hofbeck, M., Peper, A. K., Kumpf, M., & Neunhoeffer, F. (2020). Evaluation of an updated sedation protocol to reduce benzodiazepines in a pediatric intensive care unit. *Curr Med Res Opin, 36*(1), 1-6. doi:10.1080/03007995.2019.1663689. Epub 2019 Sep 17. | 3b |
| Michel J, Schepan E, Hofbeck M, Engel J, Simma A, Neunhoeffer F. Implementation of a Delirium Bundle for Pediatric Intensive Care Patients. Front Pediatr. 2022;10:826259. | 4a |
| Mondardini, M. C., Daverio, M., Caramelli, F., Conti, G., Zaggia, C., Lazzarini, R., ... & Amigoni, A. (2022). Dexmedetomidine for prevention of opioid/benzodiazepine withdrawal syndrome in pediatric intensive care unit: Interim analysis of a randomized controlled trial. Pharmacotherapy: The Journal of Human Pharmacology and Drug Therapy, 42(2), 145-153 | 3b |
| Morton SU, Labrecque M, Moline M, Hansen A, Leeman K. Reducing Benzodiazepine Exposure by Instituting a Guideline for Dexmedetomidine Usage in the NICU. Pediatrics. 2021;148(5) | 4b |
| Muirhead, R., & Kynoch, K. (2019). Implementation of an opioid weaning protocol to improve pain management, and to prevent or decrease iatrogenic withdrawal syndrome in the neonatal intensive care. *Int J Evid Based Healthc, 17*(3), 147-156. doi:10.1097/XEB.0000000000000169 | 4c |
| Muthu Chidambaram N. An audit of protocolized sedation among mechanically ventilated children in a pediatric intensive care unit. Indian Journal of Critical Care Medicine. 2020;24(SUPPL 2):S58 | 1a |
| Nasser, W. (2019). Management of iatrogenic withdrawal syndrome in the pediatric patient to reduce length of stay. *Critical Care Medicine, 47*(1). | 1a |
| Nct. (2015). Pilot Study for Sedation Interruption in Children. *https://clinicaltrials.gov/show/NCT02426320*. Retrieved from https://www.cochranelibrary.com/central/doi/10.1002/central/CN-01505986/full | 1b |
| Nct. (2005). Sedation Management in Pediatric Patients Supported on Mechanical Ventilation. *https://clinicaltrials.gov/show/NCT00142766*. Retrieved from https://www.cochranelibrary.com/central/doi/10.1002/central/CN-02029668/full | 1b |
| Nct. (2012). Sedation and Ventilator Weaning Protocol in PICU. Clinicaltrials.gov. Retrieved from https://clinicaltrials.gov/ct2/show/NCT04788589 | 1b |
| O'Donnell, K., Morrill, D., & Solodiuk, J. (2017). Pediatric sedation weaning protocol decreases prescriptions for high risk opioids. *Clinical Toxicology, 55*(7), 695-696. | 1a |
| Pappachan, J., et al. (2021). P0057 / #885: AN ANALYSIS OF THE INCIDENCE OF IATROGENIC WITHDRAWAL SYNDROME BEFORE AND AFTER PARTICIPATION IN AN RCT - SANDWICH. Pediatric Critical Care Medicine: 22(1 3S), 62. doi: 10.1097/01.pcc.0000738572.44490.b7 | 1a |

| Reference | Reason for exclusion |
|---|---|
| Pratap N, Donnellan A, Sawyer J, Peach A, Staveski S. Reducing opioid use in the pediatric cardiac intensive care unit a quality improvement project. Cardiology in the Young. 2017;27(4):S333-S4 | 1a |
| Rushforth, K. (2005). A randomised controlled trial of weaning from mechanical ventilation in paediatric intensive care (PIC). Methodological and practical issues. *Intensive Crit Care Nurs, 21*(2), 76-86. doi:10.1016/j.iccn.2004.07.009. | 4b |
| Rushforth, K. (2013). The challenges of conducting a randomised controlled trial of nurse led ventilation weaning. *Intensive care medicine, 39*, S9-. Retrieved from https://www.cochranelibrary.com/central/doi/10.1002/central/CN-01010679/full | 1b |
| Saelim K, Chavananon S, Ruangnapa K, Prasertsan P, Anuntaseree W. Effectiveness of Protocolized Sedation Utilizing the COMFORT-B Scale in Mechanically Ventilated Children in a Pediatric Intensive Care Unit. J Pediatr Intensive Care. 2019;8(3):156-63 | 4d |
| Sanavia E, Mencía S, Lafever SN, Solana MJ, Garcia M, López-Herce J. Sedative and analgesic drug rotation protocol in critically Ill children with prolonged sedation. Evaluation of implementation and efficacy to reduce withdrawal syndrome. Pediatr Crit Care Med. (2019) 20:1111–7. doi: 10.1097/PCC.0000000000002071 | 3b |
| Schmees, L., Quiroz, W., Stitt, G., & Pinto, V. (2019). Development of risk-based sedation weaning protocol for opioid and benzodiazepine habituation. *Critical Care Medicine, 47*(1). | 1a |
| Schneider, J. B., Sweberg, T., Asaro, L. A., Kirby, A., Wypij, D., Thiagarajan, R. R., . . . Randomized Evaluation of Sedation Titration for Respiratory Failure Study, I. (2017). Sedation Management in Children Supported on Extracorporeal Membrane Oxygenation for Acute Respiratory Failure. *Critical Care Medicine, 45*(10), e1001-e1010. doi:10.1097/CCM.0000000000002540 | 3b |
| Schultz, T. R., Lin, R. J., Watzman, H. M., Durning, S. M., Hales, R., Woodson, A., ... & Godinez, R. I. (2001). Weaning children from mechanical ventilation: a prospective randomized trial of protocol-directed versus physician-directed weaning. Respiratory care, 46(8), 772-782 | 4b |
| Shackelford, M., & Kudchadkar, S. (2018). Optimizing goal-directed sedation with the state behavioral scale in the PICU. *Critical Care Medicine, 46*, 639. | 1a |
| Shildt, N., Traube, C., Dealmeida, M., Dave, I., Gillespie, S., Moore, W., ... & Kamat, P. P. (2021). "Difficult to Sedate": successful implementation of a Benzodiazepine-Sparing Analgosedation-Protocol in mechanically ventilated children. *Children*, *8*(5), 348. | 3b |
| Silver, G. H., Kearney, J. A., Bora, S., De Souza, C., Giles, L., Hrycko, S., ... & PATHWAYS FOR CLINICAL CARE WORKGROUP. (2019). A clinical pathway to standardize care of children with delirium in pediatric inpatient settings. *Hospital Pediatrics*, *9*(11), 909-916. | 4c |

| Reference | Reason for exclusion |
|---|---|
| Simone, S., Edwards, S., Lardieri, A., Walker, L. K., Graciano, A. L., Kishk, O. A., & Custer, J. W. (2017). Implementation of an ICU Bundle: An Interprofessional Quality Improvement Project to Enhance Delirium Management and Monitor Delirium Prevalence in a Single PICU. *Pediatr Crit Care Med, 18*(6), 531-540. doi:10.1097/PCC.0000000000001127. | 3b |
| Solodiuk, J. C., Greco, C. D., O'Donnell, K. A., Morrill, D. R., & Curley, M. A. Q. (2019). Effect of a Sedation Weaning Protocol on Safety and Medication Use among Hospitalized Children Post Critical Illness. *Journal of pediatric nursing, 49*, 18-23. doi:10.1016/j.pedn.2019.08.001. Epub 2019 Aug 27. | 4b |
| Staveski, S. L., Wu, M., Tesoro, T. M., Roth, S. J., & Cisco, M. J. (2017). Interprofessional Team's Perception of Care Delivery After Implementation of a Pediatric Pain and Sedation Protocol. *Critical care nurse, 37*(3), 66-76. doi:10.4037/ccn2017538 | 4c |
| Swartzman, C., Andrews, P., Feibelman, R., Penoyer, D., & Sole, M. (2016). Outcomes associated with implementation of a pain, agitation, and delirium guideline in the ICU. *Critical Care Medicine, 44*(12), 375. | 1a |
| Thomas, M., Dhanani, S., Irwin, D., Writer, H., & Doherty, D. (2010). Development, dissemination and implementation of a sedation and analgesic guideline in a pediatric intensive care unit...it takes creativity and collaboration. Dynamics, 21(4), 16-25. | 4c |
| Torres, N. L., Selaya, J. S., & Hernández, M. H. (2021). P0050/# 714: EFFECTIVENESS OF IMPLEMENTING A SEDATION AND WEANING PROTOCOL IN THE PEDIATRIC INTENSIVE CARE UNIT. *Pediatric Critical Care Medicine*, *22*(Supplement 1 3S), 59. | 1a |
| Tume LN, Blackwood B, McAuley DF, Morris K, Peters MJ, Jordan J, et al. Using the TIDieR checklist to describe the intervention of the Sedation and Weaning in Children (SANDWICH) trial. Nurs Crit Care. 2022. | 1c |
| Vet, N. J., de Wildt, S. N., Verlaat, C. W., Knibbe, C. A., Mooij, M. G., van Woensel, J. B., . . . de Hoog, M. (2016). A randomized controlled trial of daily sedation interruption in critically ill children. Intensive care medicine, 42(2), 233-244. | 3b |
| Vyas, Dipen MD*,†; Quinones Cardona, Vilmaris MD*,†; Carroll, Amanda MSN*,‡; Markel, Catherine BSN*,‡; Young, Megan PharmD, BCPS*,§; Fleishman, Rachel MD¶ Standardized Scoring Tool and Weaning Guideline to Reduce Opioids in Critically Ill Neonates, Pediatric Quality and Safety: May/June 2022 - Volume 7 - Issue 3 - p e562 doi: 10.1097/pq9.0000000000000562 | 3b |
| Waak, M., Harnischfeger, J., Ferguson, A., Gibbons, K., Nguyen, K. H., & Long, D. (2022). Every child, every day, back to play: the PICUstars protocol-implementation of a nurse-led PICU liberation program. BMC pediatrics, 22(1), 1-18. | 1b |

| **Reference** | **Reason for exclusion** |
|---|---|
| Weatherhead, J., Niedner, M., & Kawai, Y. (2019). Nonpharmacologic bundle to reduce delirium in a pediatric intensive care unit. Critical Care Medicine, 47(1). | 1a |
| Yaghmai, B. F., Di Gennaro, J. L., Irby, G. A., Deeter, K. H., & Zimmerman, J. J. (2016). A Pediatric Sedation Protocol for Mechanically Ventilated Patients Requires Sustenance Beyond Implementation. *Pediatr Crit Care Med, 17*(8), 721-726. doi:10.1097/PCC.0000000000000846. | 3b |

1) Wrong publication:
    a. Conference proceedings/abstract
    b. Protocol
    c. Other
2) Wrong population
3) Wrong design:
    a. Cross-sectional survey
    b. No pre-/post-interventions
4) Wrong outcome:
    a. Not an algorithm/ does not include an algorithm
    b. No measurement instrument
    c. No patient outcome
    d. Researcher only algorithm
5) Wrong language

**Table S6:** Characteristics of included studies

| | Study characteristics | | | | | | Outcome | Usual care (Comparator) | Funding source (COI) |
|---|---|---|---|---|---|---|---|---|---|
| **Author** | **Country** | **Design** | **Study aim** | **Setting (type) [# beds]** | **Population** | **Sample (age)** | | | |
| **Pain** | | | | | | | | | |
| Rana et al. (2017) (3) | USA | Retrospective pre-guideline and prospective post-guideline | To assess the opioid and BZD usage after implementation of a pain management guideline | 1 NICU<br>(Level IV)<br>[60] | Neonates undergoing surgical procedures and hypoxic respiratory failure requiring MV | **N** = 211<br>**I:** n = 82 (Md 33.9 GA, wks)<br>**C:** n = 129 (Md 34.5 GA, wks) | 1, 3, 5a,b | Initiation and length of treatment with narcotics and sedatives was decided by the attending neonatologist for each individual patient | NI (ND) |
| **Sedation** | | | | | | | | | |
| Hazwani et al. (2022) (4) | Saudi Arabia | Prospective before and after implementation using quality improvement methodology | To develop, implement and test a sedation protocol | 1 PICU<br>(med/surg)<br>[24] | Children 0–14 yrs predicted to require MV for >24H | **N** = 101<br>**I**: n = 31 (NI)<br>**C**: n = 70 (NI) | 4b, 10b | The optimal sedation-level were achieved by sedation infusions that required less frequent use of non-procedural intermittent sedation doses. The bedside nurses assessed | ND (ND) |

| | Study characteristics | | | | | | Outcome | Usual care (Comparator) | Funding source (COI) |
|---|---|---|---|---|---|---|---|---|---|
| Author | Country | Design | Study aim | Setting (type) [# beds] | Population | Sample (age) | | | |
| | | | | | | | | sedation levels by scoring the COMFORT-B Scale every 4 hours regularly and 30 min after each administration of intermittent sedation doses. | |
| **Withdrawal/weaning** | | | | | | | | | |
| Abdouni et al. (2016) (5) | USA | Retrospective chart review of before- after-implementation (three phases: baseline; early-; post-implementation) | To determine whether utilization of a clinical practice guideline for the care of pediatric iatrogenic opioid dependence would decrease opioid exposure and | 1 PICU (med/surg) [24] | All children on MV and received continuous opioid infusion for >7days | **N** = 176<br>**I:** n = 134 (Md 1.1 yrs)<br>**C:** n = 42 (Md 1.9 yrs) | 1, 2, 3, 4a, 5a,b, 9b | iatrogenic opioid dependence (IOD) management was undertaken at the discretion of the treating physicians on a patient-by-patient basis. | ND (ND) |

| | Study characteristics | | | | | | Outcome | Usual care (Comparator) | Funding source (COI) |
|---|---|---|---|---|---|---|---|---|---|
| Author | Country | Design | Study aim | Setting (type) [# beds] | Population | Sample (age) | | | |
| | | | improve management of opioid abstinence syndrome | | | | | Approaches for discontinuation of opioid infusions and weaning intermittent opioids varied | |
| Amirnovin et al. (2018) (6) | USA | Single-center prospective study, pre- and post-intervention | To study the effectiveness of a risk-stratified opioid and benzodiazepine weaning protocol to reduce drug burden without increasing withdrawal symptoms in cardiac pediatric patients | 1 CICU (cardiac) [24] | All children with an acquired or congenital cardiac disease exposed to ≥7 days of scheduled opioids and/or BZD | **N** = 119<br>**I:** n = 64 (Md 10 d)<br>**C:** n = 55 (Md 10 d) | 1, 2, 3, 4a,b, 5a,b, 6, 9a | Weaning of opioids and benzodiazepines was at the discretion of the treating physicians | NI (ND) |
| Sanchez-Pinto et al. (2018) (7) | USA | Prospective, pre- and post-intervention | To study the effectiveness of a risk-stratified opioid | 1 PICU (mixed) [24] | All patients received scheduled opioids for >7 days | **N** = 107<br>**I:** n = 39 (mean 2.3 yrs) | 1, 2, 3, 5a,b, 6, 9a | Weaning of opioids was at the discretion of the treating physicians | ND (ND) |

| | Study characteristics | | | | | | Outcome | Usual care (Comparator) | Funding source (COI) |
|---|---|---|---|---|---|---|---|---|---|
| **Author** | **Country** | **Design** | **Study aim** | **Setting (type) [# beds]** | **Population** | **Sample (age)** | | | |
| | | | weaning protocol at decreasing the opioid drug burden in critically ill children at risk of withdrawal without increasing the amount of withdrawal symptoms | | | **C:** n = 68 (mean 2.1 yrs) | | | |
| Ford et al. (2022) (8) | USA | Retrospective cohort study | To evaluate the implementation of a pharmacist-managed opioid weaning regimen and an objective opioid withdrawal assessment tool in pediatric patients | PICU/I (med/surg) (32 (16/16)) | All children who received an opioid for ≥ 5 days and were ≤ 18 years old | **N** = 72<br>**I:** n = 36 (Md 0.48 yrs)<br>**C**: n = 36 (Md 0.84 yrs) | 1, 2, 4a, 6, 9a,b | There was no standardized weaning protocol, assessment tools, or nursing interventions and weaning off methadone was at the discretion of the attending provider | ND (ND) |

| | Study characteristics | | | | | | Outcome | Usual care (Comparator) | Funding source (COI) |
|---|---|---|---|---|---|---|---|---|---|
| **Author** | **Country** | **Design** | **Study aim** | **Setting (type) [# beds]** | **Population** | **Sample (age)** | | | |
| | | | requiring an opioid wean | | | | | | |
| Tiacharoen et al. (2020) (9) | Thailand | Randomized controlled trial | To compare a sedative-weaning protocol to usual care weaning on withdrawal rates | 1 PICU (NI) [NI] | All MV children 1 mth -18yrs who received continuous IV opioid or BZD infusion for >5days | **N** = 30<br>**I:** n = 19 (mean 1.6 yrs)<br>**C:** n = 11 (mean 1.7 yrs) | 1, 3, 6, 9a,b | The control group was managed by four pediatric intensivists. | ND (ND) |
| Vipond et al. (2018) (10) | USA | Prospective cohort study with retrospective control to compare results of implementing the protocol | To compare a withdrawal taper program to standard care on length of taper, LOS and provider satisfaction/ to reduce the length of taper of methadone and lorazepam for patients with iatrogenic withdrawal | 1 PICU (med/surg) [12] | All children receiving continuous opioid and/or BZD infusions | **N** = 49<br>**I:** n = 25 (NI)<br>**C:** n = 24 (NI) | 1, 2, 3, 4b, 5a, 9a | A retrospective chart review for comparison data. | ND (NI) |
| Walters et al. | USA | Retrospective cohort, before and | To compare the duration of methadone | 1 PICU (NI) [NI] | Children <18 yrs who received | N = 46 | 1, 3, 4a, 9b | Patients were weaned off of | ND (ND) |

| | Study characteristics | | | | | | Outcome | Usual care (Comparator) | Funding source (COI) |
|---|---|---|---|---|---|---|---|---|---|
| **Author** | **Country** | **Design** | **Study aim** | **Setting (type) [# beds]** | **Population** | **Sample (age)** | | | |
| (2021) (11) | | after protocol implementation | exposure and the incidence of withdrawal symptoms pre- and post-protocol implementation | | continuous morphine or fentanyl infusions in the PICU > 5 days prior to the methadone wean | I: n = 25 (Md 5.3 mths)<br><br>C: n = 21 (Md 5.8 mths) | | methadone on a case-by-case basis, with dose adjustments made at the discretion of the critical care physician | |
| Wilson et al. (2021) (12) | USA | Retrospective cohort study, chart review, before and after implementation | To study the effects of a weaning protocol on length of wean for opioids and BZD and decrease LOS | 1 PICU<br><br>(med/surg)<br><br>[24] | Children receiving opioid and/or BZD infusions for >3 days | **N** = 177<br><br>**I:** n = 102 (Md 0.4 yrs)<br><br>**C:** n = 75 (Md 0.6 yrs) | 1, 3, 4a,b, 5a,b, 9a | patients were weaned from opioids and benzodiazepines per attending physician discretion | ND (ND) |
| **Pain+sedation** | | | | | | | | | |
| Cavrois-Pietrzak (2018) (13) | France | Single-center prospective observational study (before-, after-implementation) (phase 3) | To evaluate the effects of implementation of a pain-sedation protocol managed by nurses on the duration of MV | 1 PICU<br><br>(med/surg)<br><br>[NI] | Children 28 days to 17 years who required MV for > 24H | **N** = 98<br><br>**I:** n = 48 (Md 23.5 mths) (phase 3)<br><br>**C:** n=50 (Md 13.5 mths) | 1, 2, 3, 4a,b, 5a,b, 6, 10b | All patients ventilated for > 24 H received a hypnotic and morphine unless contraindicated. Comfort was assessed using the | NI (NI) |

| | Study characteristics | | | | | | Outcome | Usual care (Comparator) | Funding source (COI) |
|---|---|---|---|---|---|---|---|---|---|
| Author | Country | Design | Study aim | Setting (type) [# beds] | Population | Sample (age) | | | |
| | | | | | | | | COMFORT-B scale and a global sedation (over-sedated/well-sedated/not sedated). The management and titration was at the discretion of the physician | |
| Deeter et al (2011) (14) | USA | Retrospective cohort study with historical controls | To evaluate the effect of a nursing-driven sedation protocol for mechanically ventilated patients on duration of use of analgesic and sedative medications | 1 PICU (cardiac med/surg) [31] | Children requiring mechanical ventilation >48 H | **N** = 319 **I:** n = 166 (mean 2.6 yrs) **C:** n = 153 (mean 3 yrs) | 1, 3, 4a,b | Patients were managed by using independent physician-directed approaches requiring specific physician's orders for sedation selection, dosing, and titration. | NIH (ND) |

<table>
<tr><th rowspan="2">Author</th><th colspan="6">Study characteristics</th><th rowspan="2">Outcome</th><th rowspan="2">Usual care (Comparator)</th><th rowspan="2">Funding source (COI)</th></tr>
<tr><th>Country</th><th>Design</th><th>Study aim</th><th>Setting (type) [# beds]</th><th>Population</th><th>Sample (age)</th></tr>
<tr><td>Deindl et al. (2013) (15)</td><td>Austria</td><td>Retrospective pre – and prospective post-implementation</td><td>To evaluate the implementation of a neonatal pain and sedation protocol on clinical outcomes</td><td>2 NICUs in 1 hospital<br><br>(Level III)<br><br>[10 in each]</td><td>Infants in the NICU</td><td>N = 949<br><br>I: n=465 (mean 32.6 GA, wks)<br><br>C: n = 484 (mean 31.9 GA, wks)</td><td>1, 3, 4a,b, 5a,b, 8</td><td rowspan="2">There was no instrument to assess nor a protocol to treat pain, agitation, and sedation. The management of pain, agitation, and sedation was based on irregular and subjective evaluations of the patient's condition in terms of pain and sedation.</td><td>ND (ND)</td></tr>
<tr><td>Deindl et al. (2016) (16)</td><td>Austria</td><td>Retrospective cohort, pre– post-implementation</td><td>To examine the effect of the systematic assessment and management of pain and sedation on the in-hospital outcomes of extremely preterm infants</td><td>2 NICUs in 1 hospital<br><br>(Level III)<br><br>[10 in each]</td><td>Extremely preterm neonates</td><td>N = 140<br><br>I: n = 65 (mean 26.4 GA in wks)<br><br>C: n = 75 (mean 26.5 GA in wks)</td><td>1, 3, 5a,b</td><td>VUK (ND)</td></tr>
<tr><td>Dreyfus et al. (2017) (17)</td><td>France</td><td>Before and after protocol implementation</td><td>To evaluate the impact of a nurse-driven sedation protocol implemented at a PICU on outcomes</td><td>1 PICU<br><br>(med/surg)<br><br>[23]</td><td>Children 0–18 yrs who required MV for at least 24H</td><td>N = 197<br><br>I: n = 93 (mean 5.2 yrs)<br><br>C: n = 104 (mean 4.9 yrs)</td><td>1, 3, 4a,b, 6, 7, 8, 10b</td><td>Usual care prior to implementation of protocol</td><td>APICIL foundation & ALLP Association (ND)</td></tr>
</table>

| | Study characteristics | | | | | | | | |
|---|---|---|---|---|---|---|---|---|---|
| **Author** | **Country** | **Design** | **Study aim** | **Setting (type) [# beds]** | **Population** | **Sample (age)** | **Outcome** | **Usual care (Comparator)** | **Funding source (COI)** |
| | | | (duration of MV, cumulative doses of drugs (continuous and bolus), duration of drugs, LOS in PICU, incidence of ventilator-associated pneumonia, and occurrence of withdrawal symptoms) | | | | | | |
| Gaillard-Le Roux et al. (2017) (18) | France | Prospective before and after implementation | To evaluate the impact of a nurse-driven sedation protocol on clinical outcomes (MV, total daily dose of sedatives, and complications of sedation) | 1 PICU (med/surg) [12] | Children 28 days to 18 yrs, requiring MV for at least 24 hrs | **N** = 194<br>**I:** n = 97 (Md 5.9 mths)<br>**C:** n = 97 (Md 6.1 mths) | 1, 2, 3, 4b, 6, 10b | During control phase, no protocol was used to manage sedation | NI (ND) |

| | Study characteristics | | | | | | Outcome | Usual care (Comparator) | Funding source (COI) |
|---|---|---|---|---|---|---|---|---|---|
| **Author** | **Country** | **Design** | **Study aim** | **Setting (type) [# beds]** | **Population** | **Sample (age)** | | | |
| Ista et al. (2009) (19) | Netherlands | Pretest-, post-test intervention | To study the effects of a sedation protocol on doses of sedatives and nurses' compliance with the protocol | 1 PICU<br><br>(NI)<br><br>[15] | Children up to 3 yr old, MV and on continuous IV sedative infusion for >48H and/or receiving midazolam or morphine | **N** =187<br><br>**I:** (posttest): n = 29 (Md 3.1 mths)<br><br>**I:** (long run): n = 131 (NI)<br><br>**C**: n = 27 (Md 4.1 mths) | 7, 10b | Pain/sedation were managed at the discretion of the attending physician | Dutch Organization for Scientific Research (NI) |
| Kleiber et al. (2016) (20) | Australia | Retrospective cohort study with matched controls | To compared cardiovascular stability of young infants at high risk of low cardiac output syndrome receiving two sedation practices: the routine early use of continuous midazolam infusion (pre-emptive sedation) and the | 1 PCICU<br><br>(cardiac)<br><br>[15] | Children between a postconceptional age >37 weeks and postnatal age <6 months who received cardiopulmonary bypass time greater than 150 minutes | **N** = 66<br><br>**I**; n = 33 (Md 9 d)<br><br>**C**: n = 33 (Md 8 d) | 1,3,5a,b | Restrospective matched controls, patients treated with discretionary use of sedatives | SdW institution consulted for Koehler Chemie and received grant supports from European Union, ZonMW, Nuts Ohra, and Novartis (investigator-initiated research) all other authors ND (NI) |

| | Study characteristics | | | | | | Outcome | Usual care (Comparator) | Funding source (COI) |
|---|---|---|---|---|---|---|---|---|---|
| **Author** | **Country** | **Design** | **Study aim** | **Setting (type) [# beds]** | **Population** | **Sample (age)** | | | |
| | | | discretionary use of sedative drugs tailored to the patient's clinical condition (targeted sedation) | | | | | | |
| Larson et al. (2018) (21) | Australia | Retrospective pre- and post-analgesic and sedative protocol introduction | To assess the influence of using a pain and sedation protocol on management of children admitted to PICU following cardiac surgery on clinical outcomes (frequency of pain and sedation assessment, dosage/ administration of analgesics or sedatives | 1 PICU (cardiac) [21] | Children >18 yrs, intubated and MV after cardiac surgery | **N** = 100<br>**I:** n = 50 (Md 163 d)<br>**C:** n = 50 (Md 792 d) | 1, 3, 7, 10b | Prior to the protocol there was prescription for the escalation of pharmacological treatment. Post operative pain management did not use boluses of morphine prior to increasing the rate of infusion. Clonidine was the first line sedative agent | NI (NI) |

| | Study characteristics | | | | | | Outcome | Usual care (Comparator) | Funding source (COI) |
|---|---|---|---|---|---|---|---|---|---|
| Author | Country | Design | Study aim | Setting (type) [# beds] | Population | Sample (age) | | | |
| | | | and length of ventilation) | | | | | | |
| Loberger et al. (2022) (22) | USA | Quality improvement initiative with a retrospective pre-/ prospective post-implementation design | To decrease the invasive mechanical ventilation duration | PICU<br><br>(med/surg)<br><br>(24) | Children >18 years of age requiring invasive MV via an oral or nasal endotracheal tube | **N** = 585<br><br>**I**: n = 332 (Md 57 mth )<br><br>**C**: n = 253 (Md 43 mth) | 1, 3, 5a,b, 6, 10b | No standardized processes for analgosedation and was at the discretion of the attending intensivist | None<br><br>(ND) |
| Magner et al. (2020) (23) | Ireland | Retrospective before and after design | To determine if the introduction of a standardised nurse-led analgesia and sedation practice impacts on amount of morphine administered in the first 72 hours in a population of post cardiac PICU patients | 1 PICU/ 1 HDU<br><br>(NI)<br><br>[23 (18/5)] | Children aged 1 mth - 1 yr with open cardiothoracic surgery | All admitted patients<br><br>**N** = 125<br><br>**I:** n = 64 (Md 26 wks)<br><br>**C:** n = 61 (Md 24 wks) | 1, 3 | Usual care prior to implementation of protocol | NCRC (ND) |

| | Study characteristics | | | | | | Outcome | Usual care (Comparator) | Funding source (COI) |
|---|---|---|---|---|---|---|---|---|---|
| **Author** | **Country** | **Design** | **Study aim** | **Setting (type) [# beds]** | **Population** | **Sample (age)** | | | |
| Puthoff et al. (2018) (24) | USA | Quality improvement pre- and post-intervention implementation | To decrease opioid exposure through a quality improvement initiative | 1 NICU (Level IV) [114] | All children who were postoperative for a tracheostomy | **N** = NI **I:** n = 14 **C:** n = NI (mean corrected 51 GA, wks) | 4b | Postoperative opioid use in infants that required tracheostomy tube placement varied from 6 to 148 days | NI (ND) |
| Yang et al. (2021) (25) | USA | Retrospective chart review, before and after implementation of the analgesia-sedation protocol | Develop and examine if a revised analgesia-sedation protocol reduced midazolam usage when dexmedetomidine is prioritized. | 1 PICU (NI) [NI] | Children who are MV for >24H | N = 334 I: n = 144 (Md 4.1 yrs) C: n = 190 (Md 3.9 yrs) | 1, 2, 5a,b, 10b | A sedation-analgesia strategy in mechanically ventilated children that was based on midazolam and opioid infusions as the standard approach | NI (ND) |
| **Pain+sedation+withdrawal** | | | | | | | | | |
| Curley et al. (2015) (26) | USA | Multi-center, cluster randomized clinical trial | To evaluate the effect of a nurse-implemented, goal-directed sedation protocol on duration of MV and | 31 PICUs (NI) [11 sites 12-20, 12 sites 19-28, | Children aged 2 weeks to 17 years receiving invasive MV >24H for acute airways and/or parenchymal | **N** = 2449 **I:** n = 1225 (Md 1.4 yrs) **C:** n = 1224 (Md 2.6 yrs) | 1, 2, 3, 4a,b, 5a,b, 6, 7, 8, 10a,b,c | PICUs were randomized at the PICU level, so care continued in control sites as per usual with no protocol | NHLBI, NINR, NIH[1] (ND) |

| | Study characteristics | | | | | | Outcome | Usual care (Comparator) | Funding source (COI) |
|---|---|---|---|---|---|---|---|---|---|
| **Author** | **Country** | **Design** | **Study aim** | **Setting (type) [# beds]** | **Population** | **Sample (age)** | | | |
| | | | secondary clinical outcomes in pediatric patients with acute respiratory failure | eight sites 26-43] | lung disease | | | | |
| Keogh et al. (2015) (27) | Australia | Pre-post design using historical controls | To develop and implement guidelines for sedation and analgesia management and to evaluate the impact, and acceptability and feasibility of their use in the clinical setting | 2 PICU<br><br>(med/surg)<br><br>[8] | Children older than 1 month, MV for ≥24H, not admitted for seizure management or terminal care | **N** = 138<br><br>**I:** n = 63 (Md 1.8 yrs)<br><br>**C:** n = 75 (Md 2.1 yrs) | 1, 3, 4a,b | There were was no assessment acales or standardization of practice or algorithms available. | QHNR (ND) |
| Lincoln et al. (2020) (28) | USA | Prospective study with one pre-intervention and three post-intervention | To decrease practice variation in pain and sedation management by describing | 1 PCICU<br><br>(med/surg)<br><br>[31] | Postoperative cardiovascular surgical patients | **N** = 1243<br><br>**I** 1: n = 241 (Md 2.8 yrs)<br>**I** 2: n = 374 (Md 2.1 yrs)<br>**I** 3: n = 397 | 1, 2, 3, 5a,b, 10a,b,c | Usual care prior to implementation of protocol | NI (RRT funding from Pfizer and Bristol Myers |

| | Study characteristics | | | | | | Outcome | Usual care (Comparator) | Funding source (COI) |
|---|---|---|---|---|---|---|---|---|---|
| **Author** | **Country** | **Design** | **Study aim** | **Setting (type) [# beds]** | **Population** | **Sample (age)** | | | |
| | | time intervals. | a quality improvement project that applied a pain and sedation management protocol on clinical outcomes for children recovering from cardiac surgery | | | (Md 2.2 yrs)<br><br>**C**: n = 231 (Md 1.7 yrs) | | | Squibb. All other authors DN) |
| Neunhoeffer et al. (2015) (29) | Germany | Before and after protocol implementation | To analyze if implementation of a nurse-driven, goal-directed analgesia and sedation protocol improves clinical outcomes (MV, LOS, total doses of opioids and BZD, occurrence of withdrawal symptoms) in | 1 PICU<br><br>(med/surg/ cadiac)<br><br>[14] | Medical patients 0–18 yrs, on MV and PICU LOS >24H | **N** = 337<br><br>**I:** n = 172 (mean 3 yrs)<br><br>**C:** n = 165 (mean 3.3 yrs) | 1, 3, 5a,b, 6 | Analgesia and sedation were managed by the attending physician. | Departmental resource<br><br>(ND) |

| | Study characteristics | | | | | | Outcome | Usual care (Comparator) | Funding source (COI) |
|---|---|---|---|---|---|---|---|---|---|
| Author | Country | Design | Study aim | Setting (type) [# beds] | Population | Sample (age) | | | |
| | | | critically ill non-surgical children compared to non-protocol-directed analgesia and sedation | | | | | | |
| Neunhoeffer et al. (2017) (30) | Germany | Two-phases observational study, before and after implementation | To analyze if implementation of a nurse-driven, goal-directed analgesia and sedation protocol improves clinical outcomes (MV, LOS, total doses of opioids and BZD, occurrence of withdrawal symptoms) in critically ill non-surgical children compared to non-protocol- | 1 PICU<br><br>(med/surg/ cardiac)<br><br>[14] | All surgical patients 1–16 yrs, on MV and PICU LOS >24H | **N** = 226<br><br>**C:** n = 116 (mean 86.2 mth)<br><br>**I:** n = 110 (mean 81.4 mth) | 1, 3, 5a,b, 6 | Analgesia and sedation were managed by the attending physician. | Departmental resource<br><br>(ND) |

| | Study characteristics | | | | | | Outcome | Usual care (Comparator) | Funding source (COI) |
|---|---|---|---|---|---|---|---|---|---|
| **Author** | **Country** | **Design** | **Study aim** | **Setting (type) [# beds]** | **Population** | **Sample (age)** | | | |
| | | | directed analgesia and sedation | | | | | | |
| Hanser et al. (2020) (31) | Germany | Retrospective before-after implementation | To evaluate a nurse-driven, goal directed analgesia and sedation protocol on clinical outcomes (duration of MV, LOS in PICU, peak and cumulative doses of analgesic and sedative agents and occurrence of adverse events) following corrective surgery for tetralogy of Fallot | 1 PICU (cardiac) [14] | Children following corrective surgery for tetralogy of Fallot | **N** = 65<br>**I:** n = 32 (Md 4.3 mths)<br>**C**: n = 33 (Md 3.7 mths) | 1, 3, 5a,b | Postoperative care did not include a standardized procedure, when and how to change the dosages of analgesics and sedatives in case of undersedation or oversedation | Stiftung zur Förderung der Erforschung von Zivilisationserkrankungen; Stiftung KinderHerz (ND) |
| **Pain+sedation+delirium+withdrawal** | | | | | | | | | |

| | Study characteristics | | | | | | | | |
|---|---|---|---|---|---|---|---|---|---|
| **Author** | **Country** | **Design** | **Study aim** | **Setting (type) [# beds]** | **Population** | **Sample (age)** | **Outcome** | **Usual care (Comparator)** | **Funding source (COI)** |
| Di Nardo et al. (2021) (32) | Italy | Observational pre-post implementation study | To evaluate the feasibility of implementation of a structured and interdisciplinary liberation protocol on delirium and adverse events | 1 PICU (med) [6] | Children between 1 day and 18 yrs requiring PICU admission for >72H. Excluded if early mobilization was contraindicated | N = 225<br>I: n = 88 (Md 3.3 yrs)<br>C: n = 137 (Md 4.2 yrs) | 1, 3, 5a,b | Delirium was not screening, The sedation protocol, driven by both physicians and nurses but COMFORT-B scores were not used. | NI (ND) |
| **Sedation+withdrawal** | | | | | | | | | |
| Jin et al. (2007) (33) | Korea | Retrospective pre-intervention (control) and prospective post-intervention | To identify if protocol-directed sedation with the COMFORT scale in children affects clinical outcomes (duration of MV, LOS in ICU, total amount and duration of | 1 PICU (NI) [22] | Children intubated, on MV and receiving continuous IV sedative infusion for >48H | **N** = 41<br>**I:** n = 21 (Md 20.1 mths)<br>**C:** n = 20 (Md 21.7 mths) | 1, 3, 4b, 5a,b, 6 | Retrospective chart review, prior to implementation | NI (NI) |

| | Study characteristics | | | | | | Outcome | Usual care (Comparator) | Funding source (COI) |
|---|---|---|---|---|---|---|---|---|---|
| **Author** | **Country** | **Design** | **Study aim** | **Setting (type) [# beds]** | **Population** | **Sample (age)** | | | |
| | | | sedatives and withdrawal symptoms) | | | | | | |
| **Pain+withdrawal** | | | | | | | | | |
| Stetson et al. (2020) (34) | USA | Quality improvement project, pre- and post-implementation | To compare a quality improvement project to improve pain management to reduce the mean daily opioid exposure | 1 NICU (Level IV) [35] | Infants under 1,250 g at birth | **N** = 183<br>**I:** n = 111 (mean 26.7 GA, wks)<br>**C**: n = 72 (mean 26.6 GA, wks) | 1 | Usual care prior to implementation of protocol | NI (ND) |

**NI** = no information; **—** = not applicable; H = hour; Hs = hours
**Study aim**: BZD = benzodiazepine; LOS = length of stay; MV = mechanical ventilation
**Setting** : PICU = pediatric intensive care unit; NICU = neonatal intensive care unit; CSICU = cardiac surgical intensive care unit ; med/surg = medical-surgical; PCICU = pediatric cardiac intensive care unit; HDU = high dependency unit; I = intermediate
**Population:** C = control group; I = intervention group; d = days; wks = weeks; mths = months; yrs = years; GA = gestational age; Md = median
**Outcome**: 1 = ICU LOS, 2 = hospital LOS, 3 = duration MV, 4a = duration of opioids, 4b = duration of BZD, 5a = cumulative dose opioids, 5b = cumulative dose BZD, 6 = rate of withdrawal, 7 = inadequate sedation management, 8 = inadequate pain management, 9a = duration of weaning, 9b = duration of methadone, 10a = pain scoring, 10b = sedation scoring, 10c = withdrawal scoring
Funding source (COI = conflict of interest): ND = none disclosed, NIH = National Institutes of Health, VUK = Verein Unser Kind, NCRC = National Children's Research Centre, NHLBI = National Heart, Lung, and Blood Institute, NINR = National Institute of Nursing Research, QHNR = Queensland Health Nursing Research
Notes:
[1] IMC fees for serving as a medical advisor for Philips. MAM fees for serving as chair of a data and safety monitoring board for Roche-Genentech, consultant for Cerus, Quark Pharmaceuticals,Biogen, and GlaxoSmithKline. ND for all other authors

**Table S7:** Characteristics of included algorithms

| **Author** | **Eligible patient population** | **Intervention: Algorithm characteristics** | | | | | | | **Electronic health record** |
|---|---|---|---|---|---|---|---|---|---|
| | | HCP managing algorithm | Main measurement instrument | Frequency of monitoring | Timing of team review | Primary analgesic medication | Primary sedative medication | Primary weaning agent | Documentation (other method of inclusion) |
| **Pain** | | | | | | | | | |
| Rana et al. (2017) (3) | Neonates undergoing surgical procedures and hypoxic respiratory failure | NI | NIPS | NI | Daily | Morphine or fentanyl | NA | NA | NI |
| **Sedation** | | | | | | | | | |
| Hazwani et al. (2022) (4) | MV for > 24 hrs, 0 to 14 yrs old | Nurse | COMFORT-B | Q4H and Q30 mins after intervention | Daily huddles | NI | NI | NA | EHR |
| **Withdrawal/weaning** | | | | | | | | | |
| Abdouni et al. (2016) (5) | Patients with extended continuous IV opioid administration (> 7 days) for whom MV discontinuation is | Nurse | modified NAS | Q4H, or hourly if signs and symptoms of withdrawal | NI | Fentanyl | NA | Methadone | NI (Order set for MDs) |

| | | | | | | | | | |
|---|---|---|---|---|---|---|---|---|---|
| | anticipated within 24 - 48 Hrs | | | | | | | | |
| Amirnovin et al. (2018) (6)<br>Sanchez-Pinto et al. (2018) (7) | Patients ready for weaning of opioid or BZD and whom received medications for > 7 days | Nurse | WAT-1 | Q6H | Daily | Fentanyl or hydromorphone | Lorazepam or midazolam | BZD: lorazepam<br><br>Opioid: methadone or hydromorphone | NInt |
| Ford et al. (2022) (8) | All children who received an opioid for ≥ 5 days and were ≤ 18 years old | PharmacistNurse | WAT-1 | Q12H | Q12H pharmacist meetings | Fentanyl or morphine or hydromorphone | NA | Methadone | EHR |
| Tiacharoen et al. (2020) (9) | MV patients receiving continuous BDZ or opioid infusions for > 5 days | Nurse | WAT-1 | Q4H | NI | Morphine or fentanyl | Midazolam | Lorazepam or methadone | NI |
| Vipond et al. (2018) (10) | All patient receiving continuous opioid and/or benzodiazepine infusions | Pharmacist, Critical care physicians, Nurse | WAT-T | Q12H | Daily by pharmacist | NA | NA | Lorazepam or methadone | NI |

| | | | | | | | | | |
|---|---|---|---|---|---|---|---|---|---|
| Walters et al. (2021) (11) | Patients who received continuous morphine or fentanyl for > 5 days | Nurse | WAT-1 | Q12H | NI | Morphine or fentanyl | NA | Methadone | NI |
| Wilson et al. (2021) (12) | MV patients (1mth – 18yrs) receiving opioid or benzodiazepine continuous infusion for > 5 days | Nurse | WAT-1 | Q12H and after each rescue dose | Daily using electronic shreadsheet | Morphine or fentanyl | Midazolam | Methadone or lorazepam | EHR |
| **Pain+sedation** | | | | | | | | | |
| Cavrois-Pietrzak (2018) (13) | Patients who are MV > 24h, aged 28 days to 17 yrs old | Nurse | COMFORT-B | Initiation: first assessment after 5 min. If >17 Q5min x2, if <17 Q1H. Re-assessment: > 17 Q30min Maintenance: Q4H | Daily | Morphine | Midazolam | NA | NI |
| Deeter et al (2011) (14) | Patients admitted already intubated or | Nurse | Seattle PICU Comfort Tool | Q15min until comfort goal | Daily rounds | Morphine | Lorazepam | NA | NI |

| | | | | | | | | | |
|---|---|---|---|---|---|---|---|---|---|
| | those requiring intubation during admission | | | achieved for first 2Hs, Q4H for 24Hs, Q12H until extubation | | | | | |
| Deindl et al. (2013) (15)<br>Deindl et al. (2016) (16) | Neonate patients receiving continuous analgesia or sedation; in those receiving MV or continuous positive airway pressure; in patients requiring > 40% oxygen; in cases of severe dyspnea, postoperati ve care, sepsis, indwelling pleural or abdominal drains, or large skin defects; | Nurse | N-PASS | Q8H, Q30 mins after a procedure, ↑/↓ of continuous sedative of analgesic infusion | NI | Morphine or fentanyl | Midazolam | NA | N-PASS in EHR |

| | | | | | | | | | |
|---|---|---|---|---|---|---|---|---|---|
| | and in patients receiving palliative care | | | | | | | | |
| Dreyfus et al. (2017) (17) | 0-18 years, MV for >24h | Nurse | COMFORT-B | Initiation Q1H until optimal range reached than Q4H | NI | Sufentanil | Midazolam or ketamine | NA | NI |
| Gaillard-Le Roux et al. (2017) (18) | Patients on MV, 28 days – 18 yrs, using sedatives and analgesics for >24 hrs | Nurse | COMFORT-B | Q30 mins until optimal sedation levels reached, Q3H or PRN | Daily | Morphine | Midazolam | NA | NI |
| Ista et al. (2009) (19) | MV neonates and infants up to 3 years of age | Nurse | COMFORT-B + NISS | Q8H | Daily rounds | Morphine | Midazolam | NA | EHR |
| Kleiber et al. (2016) (20) | All cardiac patients, ventilated and less than 1 year old | Nurse | COMFORT-B | Q4H | NI | Morphine | clonidine | NA | NI |
| Larson et al. (2018) (21) | MV Pts from birth to 18 yrs | Nurse | COMFORT-B | Q4H | Daily rounds | Morphine | < 1 year-clonidine, > 1 year midazolam | NA | NI (order set) |
| Loberger et al. | All children who are | Nurse | Pain: FLACC | Pain: NI | Daily rounds | Morphine | dexmedetomidine | NA | NInt |

| | | | | | | | | | |
|---|---|---|---|---|---|---|---|---|---|
| (2022) (22) | ≤<18 years of age requiring invasive MV via an oral or nasal endotracheal tube | | Sedation: SBS | Sedation: Q2H | | | | | |
| Magner et al. (2020) (23) | All patients | Nurse | COMFORT-B + NRS | Initial assessment post-operative, Q2H for 8 hrs, Q2-4H thereafter | Daily rounds | Morphine | Midazolam or chloral hydrate | Clonidine | NInt |
| Puthoff et al. (2018) (24) | Postoperative tracheostomy patients | Nurse | N-PASS | NI | Daily rounds | Morphine | Midazolam | NA | EHR |
| Yang et al. (2021) (25) | MV patients for at least 24 hours | Nurse | Pain: FLACC, NRS Sedation: SBS | Q4H | Daily huddles | Dexmedetomidine | Lorazepam | NA | EHR |
| **Pain+sedation+withdrawal** | | | | | | | | | |
| Curley et al. (2015) (26) | MV >24H, aged 2 wks to 17 yrs old with acute airways and/or parenchymal | Nurse | Sedation: SBS Pain: FLACC, Ind NRS, WBFPS Withdrawal: WAT-1 | Q4H Q4H Q8H | Daily | Morphine | Midazolam | morphine or clonidine | NI |

| | | | | | | | | | |
|---|---|---|---|---|---|---|---|---|---|
| | lung disease | | | | | | | | |
| Keogh et al. (2015) (27) | MV patients for > 24hrs Hrs, aged > 1 month and not admitted for seizure management or terminal care | Nurse | Sedation: SBS<br>Pain: MAPS<br>Withdrawal: WAS | Acute phase: SBS+MAPS Q1H,<br>Plateau phase : SBS + MAPS Q4H<br>Weaning phase:<br>a) WAS = < 10: SBS, MAPS, WAS Q4H;<br>WAS = > 10: WAS Q2H | Daily | Morphine | Midazolam | Methadone and diazepam | NI |
| Lincoln et al. (2020) (28) | All cardiac patients following surgery, MV and PICU LOS >24h | Nurse | Sedation: SBS<br>Pain: FLACC, Ind NRS, WBFPS<br>Withdrawal: WAT-1 | Q8H<br>NI<br><br>NI | Daily rounds | Morphine | Midazolam | NI | EHR |
| Neunhoeffer et al. (2015) (29) | Non-surgical patients, 0 to 18 years of age, who | Nurse | Pain-sedation: COMFORT-B + NISS | Q8H<br><br>Q8H | Daily | Morphine or fentanyl | Midazolam | None | NI |

| | | | | | | | | | |
|---|---|---|---|---|---|---|---|---|---|
| | are MV for > 24hrs | | Withdrawal: SOS | | | | | | |
| Neunhoeffer et al. (2017) (30) | All postsurgical patients 1 to 16 years of age,who are receiving MV for > 24 hrs | | | | | | | | |
| Hanser et al. (2020) (31) | Postsurgical tetralogy of fallor patients < 6 months old, receiving MV > 24 hrs | | | | Daily | Morphine | Midazolam | None | NI |
| **Pain+sedation+delirium+withdrawal** | | | | | | | | | |
| Di Nardo et al. (2021) (32) | Patients aged between 1 day and 18 years who require PICU admission for > 72 hours | Nurse | Pain-sedation: COMFORT-B Delirium: CAPD Withdrawal: WAT-1 | Q6H<br><br>Q12H<br>Q12H | Daily rounds | Dexedetomidine + fentanyl | Lorazepam | None | EHR |
| **Sedation+withdrawal** | | | | | | | | | |
| Jin et al. (2007) (33) | MV and intudated patients, requiring continuous | Pharmacist | Sedation: COMFORT Withdrawal: | Q12H Q12-24H | NI | Fentanyl | Midazolam | NA | NI |

| | | | | | | | | | |
|---|---|---|---|---|---|---|---|---|---|
| | sedatives for >48hrs | | Modified FS | | | | | | |
| **Pain+withdrawal** | | | | | | | | | |
| Stetson et al. (2020) (34) | Pre-term infants with a weight less > 1,250 g in the first 2-weeks of life | Nurse | Pain: PIPP Withdrawal: Modified FS | NI NI | Daily rounds | Morphine | NA | Morphine or Dexmedetomidine | EHR |

**NI** = no information; **NA** = not applicable
**<u>Eligible patients:</u>** MV = mechanical ventilation; wks = weeks,
**<u>Measurement instruments</u>**: **Pain:** FLACC = Face, Legs, Activity, Cry, Consolibility; WBFPS = Wong-Baker Faces Pain Scale; Ind = individualized; NRS = Numeric Rating Scale; N-PASS = Neonatal Pain, Agitation, and Sedation Scale; BIIP = Behavioral Indicators of Infant Pain**;** MAPS = Multidisciplinary Assessment of Pain Scale; NIPS = Neonatal Infant Pain Scale; PIPP = Premature Infant Pain Profile; VAS = Visual Assessment Scale;
**Sedation**: SBS = State Behavioral Scale; NISS = nurse interpretation of sedation scale; COMFORT-B = COMFORT-Behavioural Scale
**Delirium**: CAPD = Cornell Assessment of Pediatric Delirium
**Withdrawal**: OBWS = Opioid and Benzodiazepine Withdrawal Score; WAT-1 = Withdrawal Assessment Tool version 1; SOS = Sophia Observation Scale; NAS = Neonatal Abstinence Scale**;** FS = Finnegan Score; WAS = Opioid Benzodiazepine Withdrawal Assessment Scale
**<u>Frequency of monitoring</u>:** Q = every; H = hour; mins = minutes; ↓ = decrease; ↑ = increase
Electronic health record: NInt = not integrated

**Table S8:** Measurement instruments and frequency of monitoring grouped by condition[∻]

| Condition | Main measurement instrument[§] | # used | Frequency of monitoring by measurement instrument (%)* | | | | | | | |
|---|---|---|---|---|---|---|---|---|---|---|
| | | | Q2h | Q3h | Q4h | Q6h | Q8h | Q12h | Range (h) | Not indicated |
| Pain | FLACC | 4 (22, 25, 26, 28) | | | 100 (25, 26) | | | | | X (22, 28) |
| | MAPS | 1 (27) | | | 100 (27) | | | | | |
| | NIIPS | 1 (3) | | | | | | | | X (3) |
| | PIPP | 1 (34) | | | | | | | | X (34) |
| Sedation | COMFORT | 1 (33) | | | | | | 100 (33) | | |
| | COMFORT-B | 1 (4) | | | 100 (4) | | | | | |
| | SBS | 5 (22, 25-28) | 20 (22) | | 60 (25-27) | | 20 (28) | | | |
| | Seattle PICU Comfort Tool | 1 (14) | | | | | | | 100 (4-12) (14) | |
| Pain-sedation | COMFORT-B | 5 (13, 17, 18, 20, 21) | | 20 (18) | 80 (13, 17, 20, 21) | | | | | |
| | COMFORT-B + NISS | 3 (19, 29, 31, 32) | | | | | 100 (19, 29, 31, 32) | | | |
| | COMFORT-B+NRS | 1 (23) | | | | | | | 100 (2-4) (23) | |
| | NPASS | 2 (15, 24) | | | | | 100 (15) | | | X (24) |
| Delirium | CAPD | 1 (32) | | | | | | 100 (32) | | |
| Withdrawal | Modified Finnegan Score | 2 (33, 34) | | | | | | | 100 (33) (12-24) | X (34) |
| | Modified NAS | 1 (5) | | | 100 (5) | | | | | |
| | SOS | 1 (29) | | | | 100 (29) | | | | |
| | WAS | 1 (27) | | | | | | | 100 (2-4) (27) | |
| | WAT-1 | 9 (6, 8-12, 26, 28, 32) | | | 12.5 (9) | 12.5 (6) | 12.5 (26) | 62.5 (8, 10-12, 32) | | X (28) |

Only first author of each grouped algorithm is referenced
§based on clinician administered measurement instruments only, self-report measurement instruments were excluded
*based on maintenance/on-going assessment, assessments upon initiation are more frequent.

## JBI Tables

**Table S9:** Quality appraisal - JBI RCT (n=2)

| **Author** | **Q 1** | **Q 2** | **Q 3** | **Q 4** | **Q 5** | **Q 6** | **Q 7** | **Q 8** | **Q9** | **Q10** | **Q11** | **Q12** | **Q13** | **Score/ 13** |
|---|---|---|---|---|---|---|---|---|---|---|---|---|---|---|
| Curley (26) | Y | Y | N | N | N | N | Y | Y | Y | Y | Y | Y | Y | 9 |
| Tiacharoen (9) | Y | Y | N | N | N | N | Y | Y | Y | Y | Y | Y | Y | 9 |

**Abbreviations for response options**: Y= yes, N = No, U = unclear, NA = not applicable (fields scored as NA are considered not relevant and are not included in the maximum possible score)

**Questions for quality appraisal of RCTs:**

Q1 = Was true randomization used for assignment of participants to treatment groups?
Q2 = Was allocation to treatment groups concealed?
Q3 = Were treatment groups similar at the baseline?
Q4 = Were participants blind to treatment assignment?
Q5 = Were those delivering treatment blind to treatment assignment?
Q6 = Were outcomes assessors blind to treatment assignment?
Q7 = Were treatment groups treated identically other than the intervention of interest?
Q8 = Was follow up complete and if not, were differences between groups in terms of their follow up adequately described and analyzed?
Q9 = Were participants analyzed in the groups to which they were randomized?
Q10 = Were outcomes measured in the same way for treatment groups?
Q11 = Were outcomes measured in a reliable way?
Q12 = Was appropriate statistical analysis used?
Q13 = Was the trial design appropriate, and any deviations from the standard RCT design (individual randomization, parallel groups) accounted for in the conduct and analysis of the trial?

frontiers

**Table S10:** Quality appraisal - JBI Quasi-experimental (n=23)

| Author | Q1 | Q2 | Q3 | Q4 | Q5 | Q6 | Q7 | Q8 | Q9 | Score/ maximum score |
|---|---|---|---|---|---|---|---|---|---|---|
| Abdouni (5) | Y | Y | Y | Y | Y | Y | Y | Y | Y | 9/9 |
| Amirnovin (6) | Y | Y | Y | Y | Y | NA | Y | Y | Y | 8/8 |
| Cavrois-Pietrzak (13) | Y | N | Y | Y | Y | NA | Y | Y | Y | 7/8 |
| Deindl (15) | Y | Y | Y | Y | Y | NA | Y | Y | Y | 8/8 |
| Di Nardo (32) | Y | N | Y | Y | Y | NA | Y | Y | Y | 7/8 |
| Dreyfus (17) | Y | Y | Y | Y | Y | Y | Y | Y | Y | 9/9 |
| Gaillard-Le Roux (18) | Y | Y | Y | Y | Y | Y | Y | Y | Y | 9/9 |
| Hanser (31) | Y | Y | Y | Y | Y | Y | Y | Y | Y | 9/9 |
| Hazwani (4) | Y | N | U | N | Y | NA | Y | Y | Y | 5/8 |
| Ista (19) | Y | Y | Y | Y | Y | Y | Y | Y | Y | 9/9 |
| Jin (33) | Y | Y | Y | Y | Y | NA | Y | Y | Y | 8/8 |
| Keogh (27) | Y | Y | Y | Y | Y | Y | Y | Y | Y | 9/9 |
| Larson (21) | Y | N | Y | Y | Y | NA | Y | Y | Y | 7/8 |
| Lincoln (28) | Y | N | Y | Y | Y | NA | Y | Y | Y | 7/8 |
| Loberger (22) | Y | N | Y | Y | Y | NA | Y | Y | Y | 7/8 |
| Magner (23) | Y | N | Y | Y | Y | Y | Y | Y | Y | 8/9 |
| Neunhoeffer (29) | Y | Y | Y | Y | Y | Y | Y | Y | Y | 9/9 |
| Neunhoeffer (30) | Y | Y | Y | Y | Y | NA | Y | Y | Y | 8/8 |
| Puthoff (24) | Y | U | Y | Y | Y | NA | Y | Y | Y | 7/8 |
| Rana (3) | Y | N | Y | Y | Y | Y | Y | Y | Y | 8/9 |
| Sanchez-Pinto (7) | Y | Y | Y | Y | Y | Y | Y | Y | Y | 9/9 |
| Stetson (34) | Y | Y | Y | Y | Y | Y | Y | Y | Y | 9/9 |
| Yang (25) | Y | N | Y | Y | Y | NA | Y | Y | Y | 7/8 |

**Abbreviations for response options**: Y= yes, N = No, U = unclear, NA = not applicable (fields scored as NA are considered not relevant and are not included in the maximum possible score)

**Questions for quality appraisal of quasi-experimental studies:**

Q1 = Is it clear in the study what is the 'cause' and what is the 'effect'?
Q2 = Were the participants included in any comparisons similar?
Q3 = Were the participants included in any comparisons receiving similar treatment/care, other than the exposure or intervention of interest?
Q4 = Was there a control group?
Q5 = Were there multiple measurements of the outcome both pre and post the intervention/exposure?
Q6 = Was follow up complete and if not, were differences between groups in terms of their follow up adequately described and analyzed?

| |
|---|
| Q7 = Were the outcomes of participants included in any comparisons measured in the same way?<br>Q8 = Were the outcomes measured in a valid and reliable way?<br>Q9 = Was appropriate statistical analysis used? |

**Table S11:** Quality appraisal - JBI Cohort (n=7)

| Author | Q1 | Q2 | Q3 | Q4 | Q5 | Q6 | Q7 | Q8 | Q9 | Q10 | Q11 | Score/maximum |
|---|---|---|---|---|---|---|---|---|---|---|---|---|
| Deeter (14) | Y | Y | Y | Y | U | Y | Y | NA | NA | NA | Y | 7/8 |
| Deindl (16) | N | Y | Y | U | U | Y | Y | NA | NA | NA | Y | 5/8 |
| Ford (8) | Y | Y | Y | Y | N | Y | Y | NA | NA | NA | Y | 6/8 |
| Kleiber (20) | N | Y | Y | Y | Y | Y | Y | NA | NA | NA | Y | 7/8 |
| Vipond (10) | Y | Y | Y | Y | N | Y | Y | NA | NA | NA | Y | 7/8 |
| Walters (11) | Y | Y | Y | NA | Y | Y | Y | NA | NA | NA | Y | 7/7 |
| Wilson (12) | U | Y | Y | Y | N | Y | Y | NA | NA | NA | Y | 6/8 |

**Abbreviations for response options**: Y= yes, N = No, U = unclear, NA = not applicable (fields scored as NA are considered not relevant and are not included in the maximum possible score)

**Questions for quality appraisal of cohort studies:**

Q1 = Were the two groups similar and recruited from the same population?
Q2 = Were the exposures measured similarly to assign people to both exposed and unexposed groups?
Q3 = Was the exposure measured in a valid and reliable way?
Q4 = Were confounding factors identified?
Q5 = Were strategies to deal with confounding factors stated?
Q6 = Were the groups/participants free of the outcome at the start of the study (or at the moment of exposure)?
Q7 = Were the outcomes measured in a valid and reliable way?
Q8 = Was the follow up time reported and sufficient to be long enough for outcomes to occur?
Q9 = Was follow up complete, and if not, were the reasons to loss to follow up described and explored?
Q10 = Were strategies to address incomplete follow up utilized?
Q11 = Was appropriate statistical analysis used?

## Subgroup analysis by algorithm type

**Figure S1**: Subgroup analysis - ICU length of stay

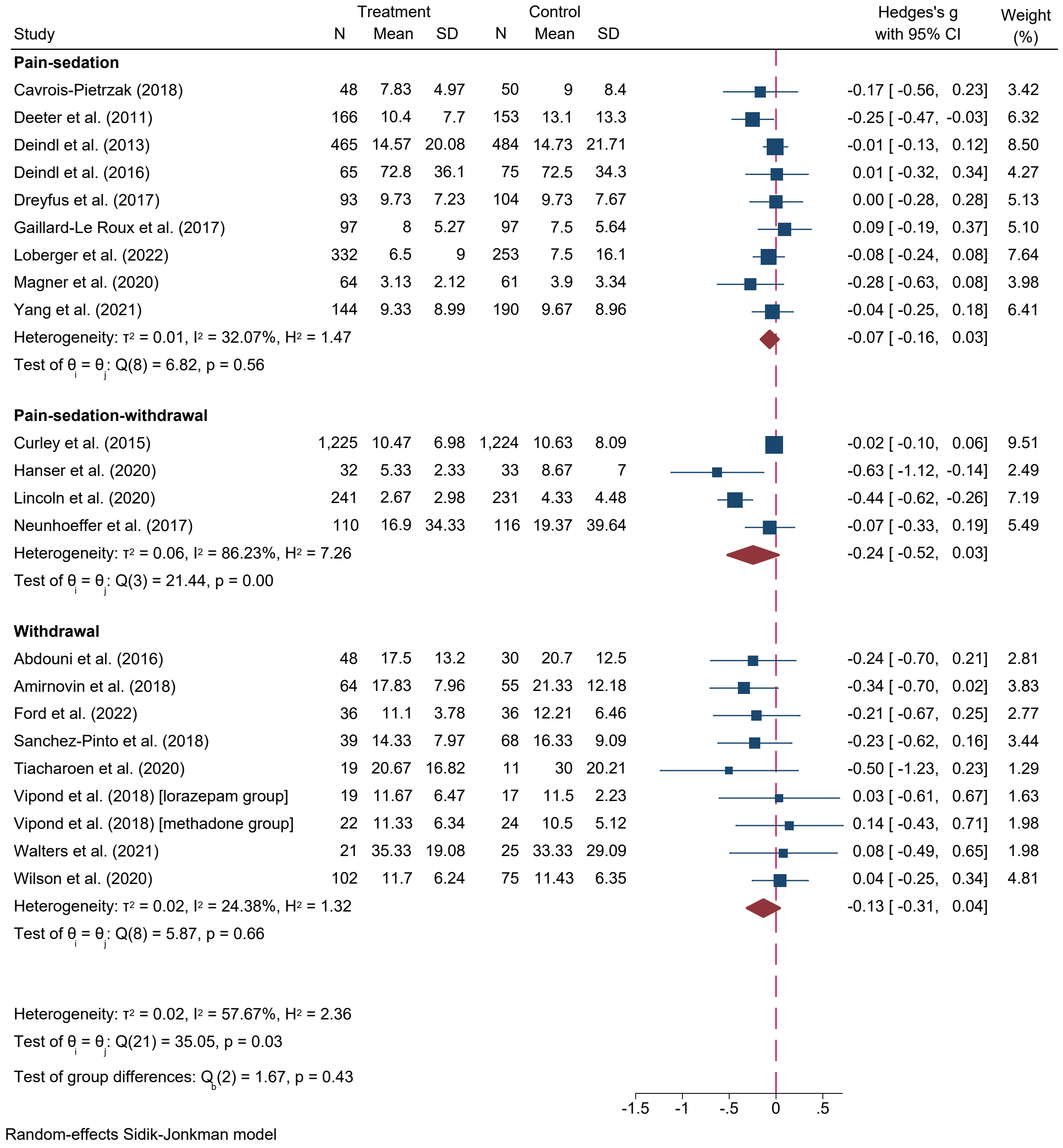

**Figure S2**: Subgroup analysis - Hospital length of stay

| Study | Treatment N | Treatment Mean | Treatment SD | Control N | Control Mean | Control SD | Hedges's g with 95% CI | Weight (%) |
|---|---|---|---|---|---|---|---|---|
| **Pain-sedation** | | | | | | | | |
| Cavrois-Pietrzak (2018) | 48 | 21.5 | 18.34 | 50 | 20.33 | 21.37 | 0.06 [ -0.33, 0.45] | 8.42 |
| Gaillard-Le Roux et al. (2017) | 97 | 19 | 14.3 | 97 | 20.33 | 15.8 | -0.09 [ -0.37, 0.19] | 10.60 |
| Yang et al. (2021) | 144 | 21.33 | 17.97 | 190 | 27.57 | 25.17 | -0.28 [ -0.50, -0.06] | 11.88 |
| Heterogeneity: $\tau^2$ = 0.01, $I^2$ = 37.29%, $H^2$ = 1.59 | | | | | | | -0.14 [ -0.35, 0.07] | |
| Test of $\theta_i = \theta_j$: Q(2) = 2.58, p = 0.28 | | | | | | | | |
| **Pain-sedation-withdrawal** | | | | | | | | |
| Curley et al. (2015) | 1,225 | 15.67 | 11.13 | 1,224 | 18 | 14.84 | -0.18 [ -0.26, -0.10] | 14.08 |
| Lincoln et al. (2020) | 241 | 6.33 | 3.72 | 231 | 10 | 8.95 | -0.54 [ -0.72, -0.36] | 12.52 |
| Heterogeneity: $\tau^2$ = 0.06, $I^2$ = 91.54%, $H^2$ = 11.82 | | | | | | | -0.35 [ -0.69, -0.00] | |
| Test of $\theta_i = \theta_j$: Q(1) = 12.54, p = 0.00 | | | | | | | | |
| **Withdrawal** | | | | | | | | |
| Abdouni et al. (2016) | 48 | 27.1 | 14.3 | 30 | 34 | 21.7 | -0.39 [ -0.85, 0.07] | 7.36 |
| Amirnovin et al. (2018) | 64 | 36.33 | 18.96 | 55 | 50.67 | 30.45 | -0.57 [ -0.94, -0.21] | 8.93 |
| Ford et al. (2022) | 36 | 18.07 | 5.71 | 36 | 23.87 | 8.65 | -0.78 [ -1.26, -0.31] | 7.07 |
| Sanchez-Pinto et al. (2018) | 39 | 29 | 20.01 | 68 | 35.33 | 23.48 | -0.28 [ -0.67, 0.11] | 8.43 |
| Vipond et al. (2018) [lorazepam group] | 19 | 17.33 | 9.61 | 17 | 17.67 | 7.26 | -0.04 [ -0.68, 0.60] | 4.98 |
| Vipond et al. (2018) [methadone group] | 22 | 17.83 | 10.3 | 24 | 15.67 | 6.3 | 0.25 [ -0.32, 0.82] | 5.75 |
| Heterogeneity: $\tau^2$ = 0.08, $I^2$ = 60.21%, $H^2$ = 2.51 | | | | | | | -0.34 [ -0.64, -0.03] | |
| Test of $\theta_i = \theta_j$: Q(5) = 9.85, p = 0.08 | | | | | | | | |
| Heterogeneity: $\tau^2$ = 0.06, $I^2$ = 76.66%, $H^2$ = 4.28 | | | | | | | | |
| Test of $\theta_i = \theta_j$: Q(10) = 27.90, p = 0.00 | | | | | | | | |
| Test of group differences: $Q_b(2)$ = 1.61, p = 0.45 | | | | | | | | |

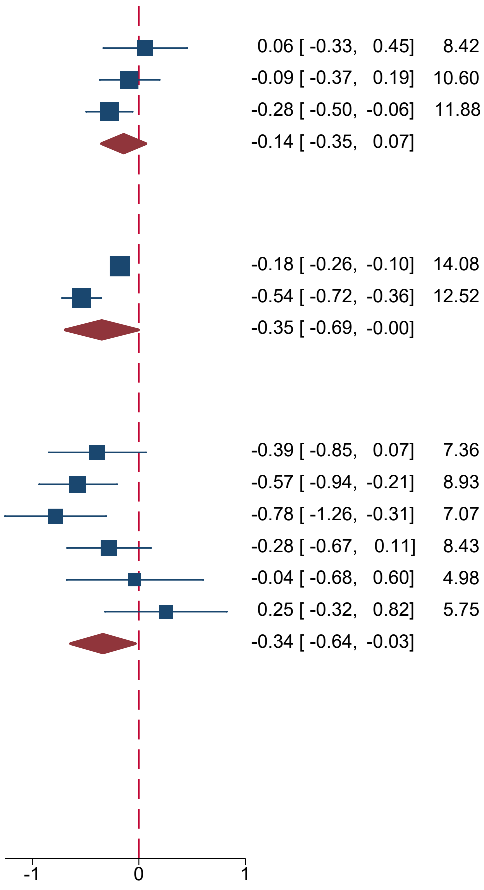


Random-effects Sidik-Jonkman model

**Figure S3**: Subgroup analysis - Length of MV

| Study | Treatment N | Treatment Mean | Treatment SD | Control N | Control Mean | Control SD | Hedges's g with 95% CI | Weight (%) |
|---|---|---|---|---|---|---|---|---|
| **Pain-sedation** | | | | | | | | |
| Cavrois-Pietrzak (2018) | 48 | 3.37 | 2.06 | 50 | 4.4 | 3.82 | -0.33 [ -0.73, 0.06] | 3.78 |
| Deeter et al. (2011) | 166 | 6.3 | 5.4 | 153 | 8.1 | 9 | -0.24 [ -0.46, -0.02] | 7.70 |
| Deindl et al. (2013) | 465 | 3.43 | 4.54 | 484 | 3.2 | 4.61 | 0.05 [ -0.08, 0.18] | 11.13 |
| Deindl et al. (2016) | 65 | 3.4 | 6.1 | 75 | 5 | 8.6 | -0.21 [ -0.54, 0.12] | 4.85 |
| Dreyfus et al. (2017) | 93 | 5.3 | 4.14 | 104 | 6.43 | 5.49 | -0.23 [ -0.51, 0.05] | 5.99 |
| Gaillard-Le Roux et al. (2017) | 97 | 5 | 3.76 | 97 | 5.17 | 3.39 | -0.05 [ -0.33, 0.23] | 5.97 |
| Loberger et al. (2022) | 332 | 3.1 | 4 | 253 | 3.95 | 5.6 | -0.18 [ -0.34, -0.01] | 9.71 |
| Heterogeneity: $\tau^2$ = 0.01, $I^2$ = 40.92%, $H^2$ = 1.69 | | | | | | | -0.13 [ -0.25, -0.02] | |
| Test of $\theta_i = \theta_j$: Q(6) = 10.60, p = 0.10 | | | | | | | | |
| **Pain-sedation-withdrawal** | | | | | | | | |
| Curley et al. (2015) | 1,225 | 7.27 | 5.27 | 1,224 | 7.43 | 6.23 | -0.03 [ -0.11, 0.05] | 12.91 |
| Neunhoeffer et al. (2017) | 110 | 9.77 | 20.81 | 116 | 18.23 | 40.23 | -0.26 [ -0.52, -0.00] | 6.47 |
| Heterogeneity: $\tau^2$ = 0.02, $I^2$ = 62.20%, $H^2$ = 2.65 | | | | | | | -0.11 [ -0.32, 0.10] | |
| Test of $\theta_i = \theta_j$: Q(1) = 2.81, p = 0.09 | | | | | | | | |
| **Withdrawal** | | | | | | | | |
| Abdouni et al. (2016) | 144 | 11.9 | 8.3 | 42 | 12.8 | 6.4 | -0.11 [ -0.46, 0.23] | 4.64 |
| Amirnovin et al. (2018) | 64 | 12.17 | 7.21 | 55 | 12.67 | 6.85 | -0.07 [ -0.43, 0.29] | 4.36 |
| Sanchez-Pinto et al. (2018) | 39 | 9.33 | 4.62 | 68 | 10.83 | 4.54 | -0.33 [ -0.72, 0.07] | 3.81 |
| Tiacharoen et al. (2020) | 19 | 12.33 | 5.61 | 11 | 19.83 | 19.51 | -0.58 [ -1.32, 0.15] | 1.34 |
| Vipond et al. (2018) [lorazepam group] | 19 | 7.33 | 3.69 | 17 | 8.33 | 2.53 | -0.31 [ -0.95, 0.34] | 1.71 |
| Vipond et al. (2018) [methadone group] | 22 | 7.83 | 4.75 | 24 | 7 | 3.15 | 0.20 [ -0.37, 0.77] | 2.11 |
| Wilson et al. (2020) | 102 | 7.33 | 3.68 | 75 | 7.97 | 5.37 | -0.14 [ -0.44, 0.15] | 5.57 |
| Heterogeneity: $\tau^2$ = 0.02, $I^2$ = 31.46%, $H^2$ = 1.46 | | | | | | | -0.16 [ -0.36, 0.04] | |
| Test of $\theta_i = \theta_j$: Q(6) = 4.04, p = 0.67 | | | | | | | | |
| Heterogeneity: $\tau^2$ = 0.01, $I^2$ = 47.34%, $H^2$ = 1.90 | | | | | | | | |
| Test of $\theta_i = \theta_j$: Q(15) = 19.31, p = 0.20 | | | | | | | | |
| Test of group differences: $Q_b(2)$ = 0.13, p = 0.94 | | | | | | | | |

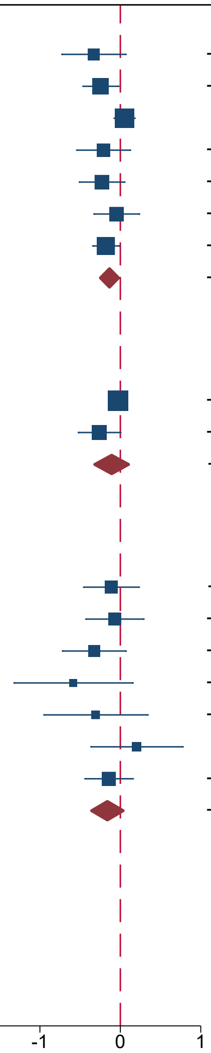


Random-effects Sidik-Jonkman model

**Figure S4**: Subgroup analysis: Duration of analgesic medications

| Study | Treatment N | Treatment Mean | Treatment SD | Control N | Control Mean | Control SD | Hedges's g with 95% CI | Weight (%) |
|---|---|---|---|---|---|---|---|---|
| **Pain-sedation** | | | | | | | | |
| Deeter et al. (2011) | 166 | 7 | 6.3 | 153 | 9.9 | 9.9 | -0.35 [ -0.57, -0.13] | 13.64 |
| Deindl et al. (2013) | 465 | 6.67 | 9.74 | 484 | 8.07 | 11.82 | -0.13 [ -0.26, -0.00] | 15.97 |
| Gaillard-Le Roux et al. (2017) | 97 | 5 | 3.01 | 97 | 4.67 | 3.01 | 0.11 [ -0.17, 0.39] | 12.03 |
| Heterogeneity: $\tau^2$ = 0.04, $I^2$ = 77.19%, $H^2$ = 4.38 | | | | | | | -0.13 [ -0.38, 0.11] | |
| Test of $\theta_i = \theta_j$: Q(2) = 6.58, p = 0.04 | | | | | | | | |
| **Pain-sedation-withdrawal** | | | | | | | | |
| Curley et al. (2015) | 1,225 | 9.67 | 7.42 | 1,224 | 11.67 | 12.62 | -0.19 [ -0.27, -0.11] | 16.84 |
| Heterogeneity: $\tau^2$ = 0.00, $I^2$ = .%, $H^2$ = . | | | | | | | -0.19 [ -0.27, -0.11] | |
| Test of $\theta_i = \theta_j$: Q(0) = 0.00, p = . | | | | | | | | |
| **Withdrawal** | | | | | | | | |
| Abdouni et al. (2016) | 48 | 11 | 6.1 | 30 | 12.8 | 4.8 | -0.32 [ -0.77, 0.14] | 8.00 |
| Amirnovin et al. (2018) | 64 | 20 | 11.38 | 55 | 33.33 | 24.36 | -0.71 [ -1.08, -0.35] | 9.80 |
| Ford et al. (2022) | 36 | 6.96 | 3.19 | 36 | 6.98 | 2.06 | -0.01 [ -0.46, 0.45] | 7.95 |
| Walters et al. (2021) | 21 | 11.33 | 6.36 | 25 | 9.67 | 9.67 | 0.20 [ -0.38, 0.77] | 6.08 |
| Wilson et al. (2020) [short wean] | 68 | 7.67 | 2.73 | 45 | 8.2 | 2.19 | -0.21 [ -0.58, 0.17] | 9.67 |
| Heterogeneity: $\tau^2$ = 0.08, $I^2$ = 60.63%, $H^2$ = 2.54 | | | | | | | -0.24 [ -0.55, 0.07] | |
| Test of $\theta_i = \theta_j$: Q(4) = 9.51, p = 0.05 | | | | | | | | |
| Heterogeneity: $\tau^2$ = 0.05, $I^2$ = 79.27%, $H^2$ = 4.82 | | | | | | | | |
| Test of $\theta_i = \theta_j$: Q(8) = 17.71, p = 0.02 | | | | | | | | |
| Test of group differences: $Q_b(2)$ = 0.30, p = 0.86 | | | | | | | | |

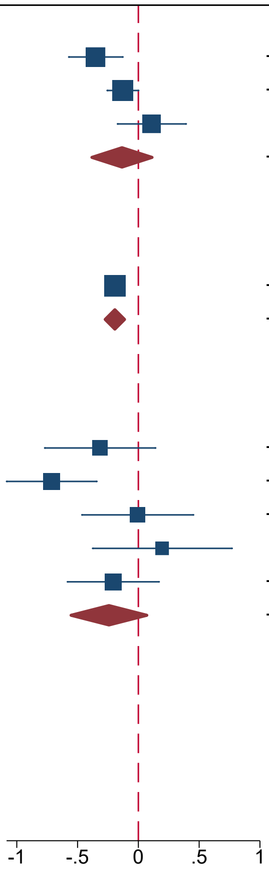


Random-effects Sidik-Jonkman model

**Figure S5**: Subgroup analysis: Duration of sedative medications

| Study | Treatment N | Treatment Mean | Treatment SD | Control N | Control Mean | Control SD | Hedges's g with 95% CI | Weight (%) |
|---|---|---|---|---|---|---|---|---|
| **Pain-sedation** | | | | | | | | |
| Deeter et al. (2011) | 166 | 1.6 | 3.7 | 153 | 6.6 | 11.2 | -0.61 [ -0.83, -0.38] | 13.40 |
| Deindl et al. (2013) | 465 | 5.03 | 6.32 | 484 | 9.33 | 13.9 | -0.40 [ -0.52, -0.27] | 14.62 |
| Gaillard-Le Roux et al. (2017) | 97 | 3.67 | 3.01 | 97 | 3.33 | 1.51 | 0.14 [ -0.14, 0.42] | 12.51 |
| Heterogeneity: $\tau^2$ = 0.13, $I^2$ = 92.13%, $H^2$ = 12.70 | | | | | | | -0.30 [ -0.72, 0.13] | |
| Test of $\theta_i = \theta_j$: Q(2) = 17.28, p = 0.00 | | | | | | | | |
| **Withdrawal** | | | | | | | | |
| Amirnovin et al. (2018) | 29 | 7.93 | 11.85 | 34 | 31.1 | 40.16 | -0.75 [ -1.25, -0.24] | 8.87 |
| Sanchez-Pinto et al. (2018) | 39 | 15 | 8.47 | 68 | 19 | 15.91 | -0.29 [ -0.68, 0.10] | 10.66 |
| Vipond et al. (2018) | 19 | 6 | 4.8 | 17 | 5.33 | 2.43 | 0.17 [ -0.47, 0.81] | 7.08 |
| Wilson et al. (2020) | 55 | 5.23 | 2.21 | 43 | 8.07 | 5.91 | -0.66 [ -1.07, -0.26] | 10.43 |
| Heterogeneity: $\tau^2$ = 0.11, $I^2$ = 64.96%, $H^2$ = 2.85 | | | | | | | -0.41 [ -0.82, -0.01] | |
| Test of $\theta_i = \theta_j$: Q(3) = 6.61, p = 0.09 | | | | | | | | |
| Heterogeneity: $\tau^2$ = 0.10, $I^2$ = 82.17%, $H^2$ = 5.61 | | | | | | | | |
| Test of $\theta_i = \theta_j$: Q(6) = 24.25, p = 0.00 | | | | | | | | |
| Test of group differences: $Q_b(1)$ = 0.16, p = 0.69 | | | | | | | | |

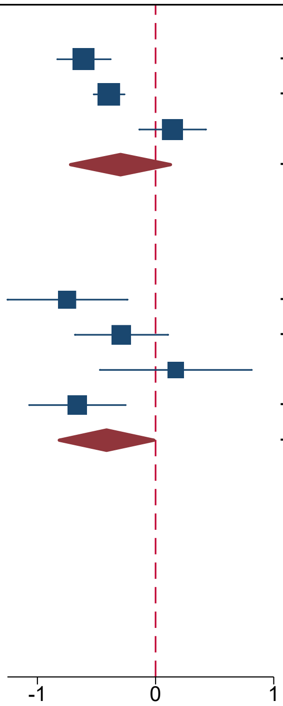


Random-effects Sidik-Jonkman model

**Figure S6**: Subgroup analysis: Cumulative dose of analgesic medications

| Study | Treatment N | Treatment Mean | Treatment SD | Control N | Control Mean | Control SD | Hedges's g with 95% CI | Weight (%) |
|---|---|---|---|---|---|---|---|---|
| **Pain-sedation** | | | | | | | | |
| Cavrois-Pietrzak (2018) | 48 | 3.53 | 3.13 | 50 | 1.7 | 2.21 | 0.67 [ 0.27, 1.08] | 5.34 |
| Deindl et al. (2013) | 465 | 6.67 | 9.74 | 484 | 8.07 | 11.82 | -0.13 [ -0.26, -0.00] | 7.21 |
| Deindl et al. (2016) | 65 | 15.3 | 41.4 | 75 | 89 | 228.3 | -0.43 [ -0.77, -0.10] | 5.87 |
| Loberger et al. (2022) | 332 | .015 | .02 | 253 | .018 | .02 | -0.15 [ -0.31, 0.01] | 7.03 |
| Heterogeneity: $\tau^2 = 0.19$, $I^2 = 94.13\%$, $H^2 = 17.03$ | | | | | | | -0.03 [ -0.47, 0.42] | |
| Test of $\theta_i = \theta_j$: Q(3) = 18.27, p = 0.00 | | | | | | | | |
| **Pain-sedation-withdrawal** | | | | | | | | |
| Curley et al. (2015) | 1,225 | 19.1 | 24.49 | 1,224 | 25.3 | 35.33 | -0.20 [ -0.28, -0.12] | 7.38 |
| Hanser et al. (2020) | 32 | 1.84 | 1.38 | 33 | 2.08 | 1.59 | -0.16 [ -0.64, 0.32] | 4.76 |
| Neunhoeffer et al. (2015) | 172 | .47 | 1 | 165 | .59 | 1.2547 | -0.11 [ -0.32, 0.11] | 6.74 |
| Neunhoeffer et al. (2017) | 110 | 24.7 | 53.26 | 116 | 23.03 | 50.03 | 0.03 [ -0.23, 0.29] | 6.42 |
| Heterogeneity: $\tau^2 = 0.00$, $I^2 = 27.98\%$, $H^2 = 1.39$ | | | | | | | -0.14 [ -0.26, -0.03] | |
| Test of $\theta_i = \theta_j$: Q(3) = 3.36, p = 0.34 | | | | | | | | |
| **Withdrawal** | | | | | | | | |
| Abdouni et al. (2016) | 48 | 1 | .9 | 30 | 2.8 | 3.7 | -0.74 [ -1.21, -0.28] | 4.87 |
| Amirnovin et al. (2018) | 64 | 32.27 | 17.29 | 55 | 57.07 | 45.99 | -0.73 [ -1.10, -0.36] | 5.60 |
| Vipond et al. (2018) | 22 | 21.6 | 16.4 | 24 | 19.7 | 16.5 | 0.11 [ -0.46, 0.68] | 4.16 |
| Wilson et al. (2020) | 45 | 37.63 | 20.53 | 68 | 52.57 | 23.43 | -0.66 [ -1.05, -0.28] | 5.49 |
| Heterogeneity: $\tau^2 = 0.11$, $I^2 = 68.87\%$, $H^2 = 3.21$ | | | | | | | -0.54 [ -0.93, -0.15] | |
| Test of $\theta_i = \theta_j$: Q(3) = 6.99, p = 0.07 | | | | | | | | |
| Heterogeneity: $\tau^2 = 0.13$, $I^2 = 91.38\%$, $H^2 = 11.60$ | | | | | | | | |
| Test of $\theta_i = \theta_j$: Q(11) = 44.70, p = 0.00 | | | | | | | | |
| Test of group differences: $Q_b(2) = 4.02$, p = 0.13 | | | | | | | | |

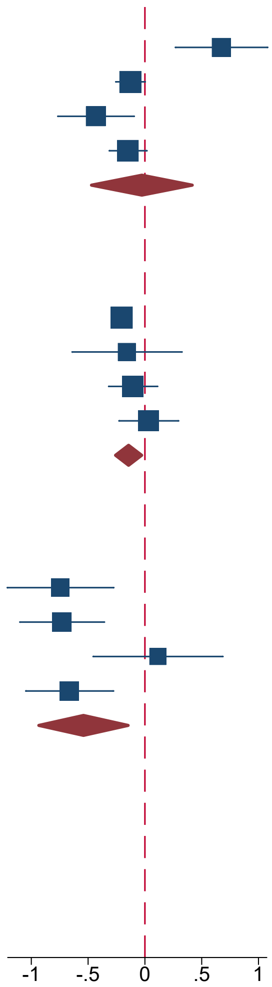


Random-effects Sidik-Jonkman model

**Figure S7**: Subgroup analysis: Cumulative dose of sedative medications

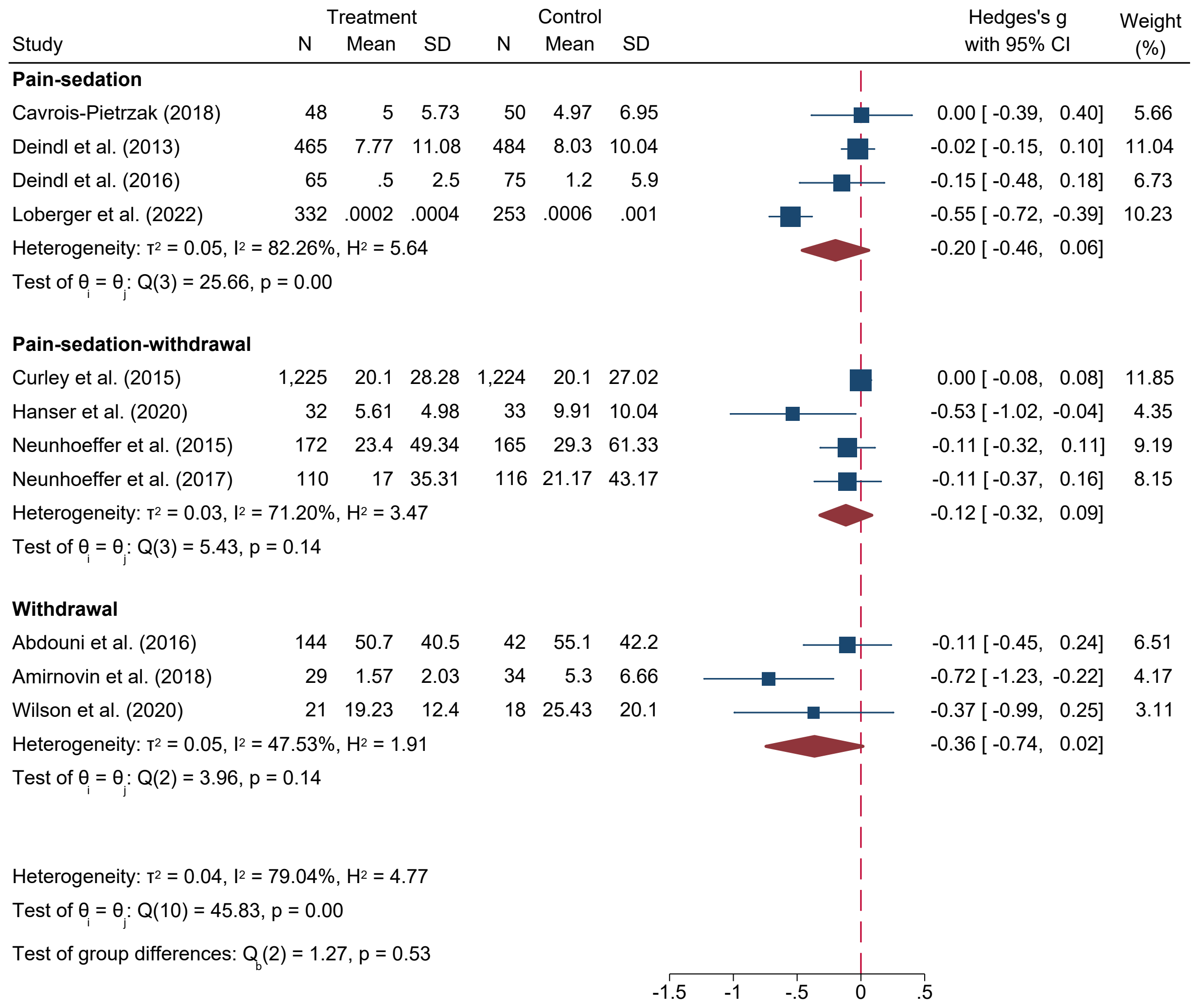


Random-effects Sidik-Jonkman model

**Figure S8**: Subgroup analysis: Incidents of iatrogenic withdrawal syndrome

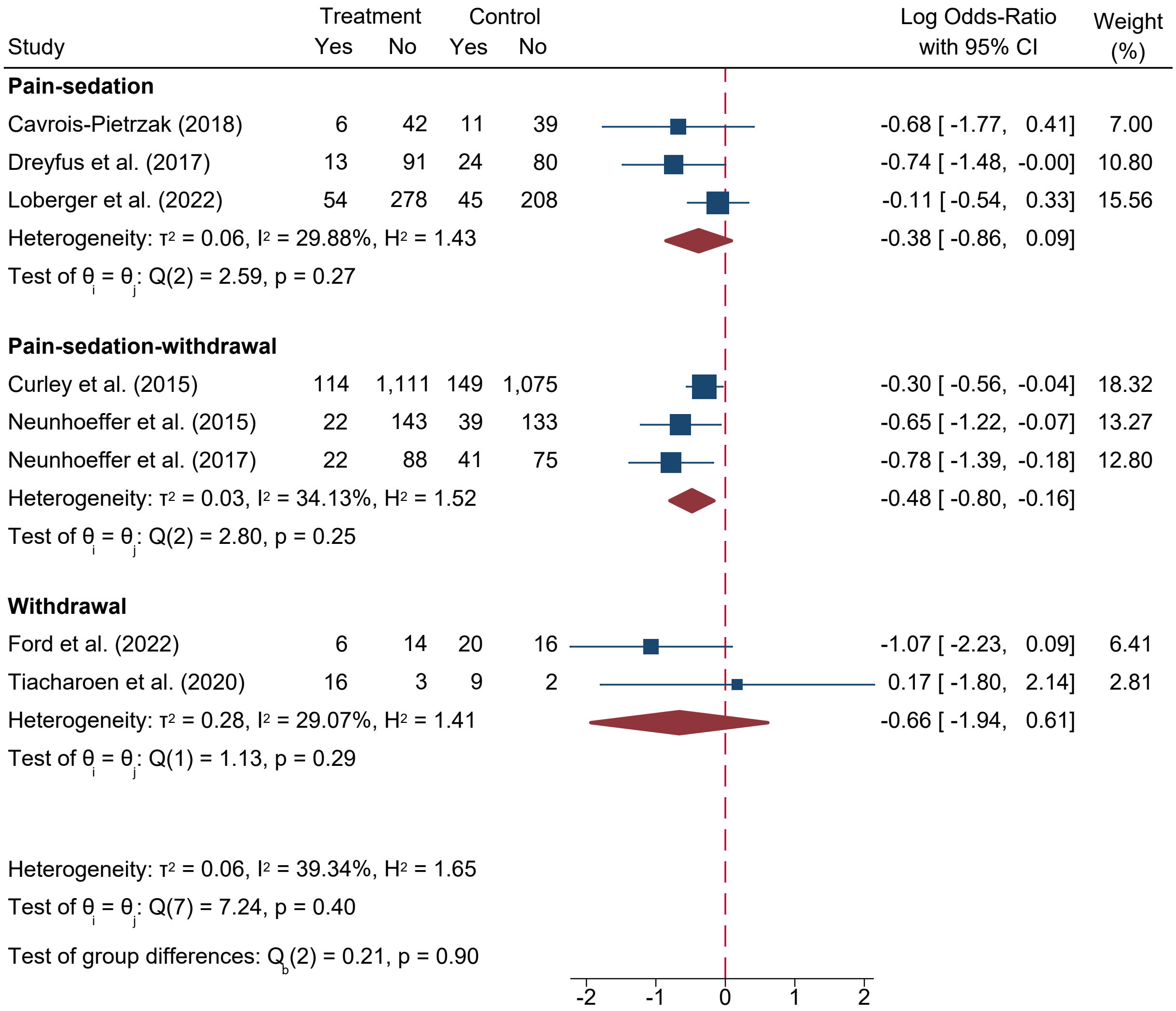


Random-effects Sidik-Jonkman model

**Figure S9**: Funnel plots for continuous outcomes of interest

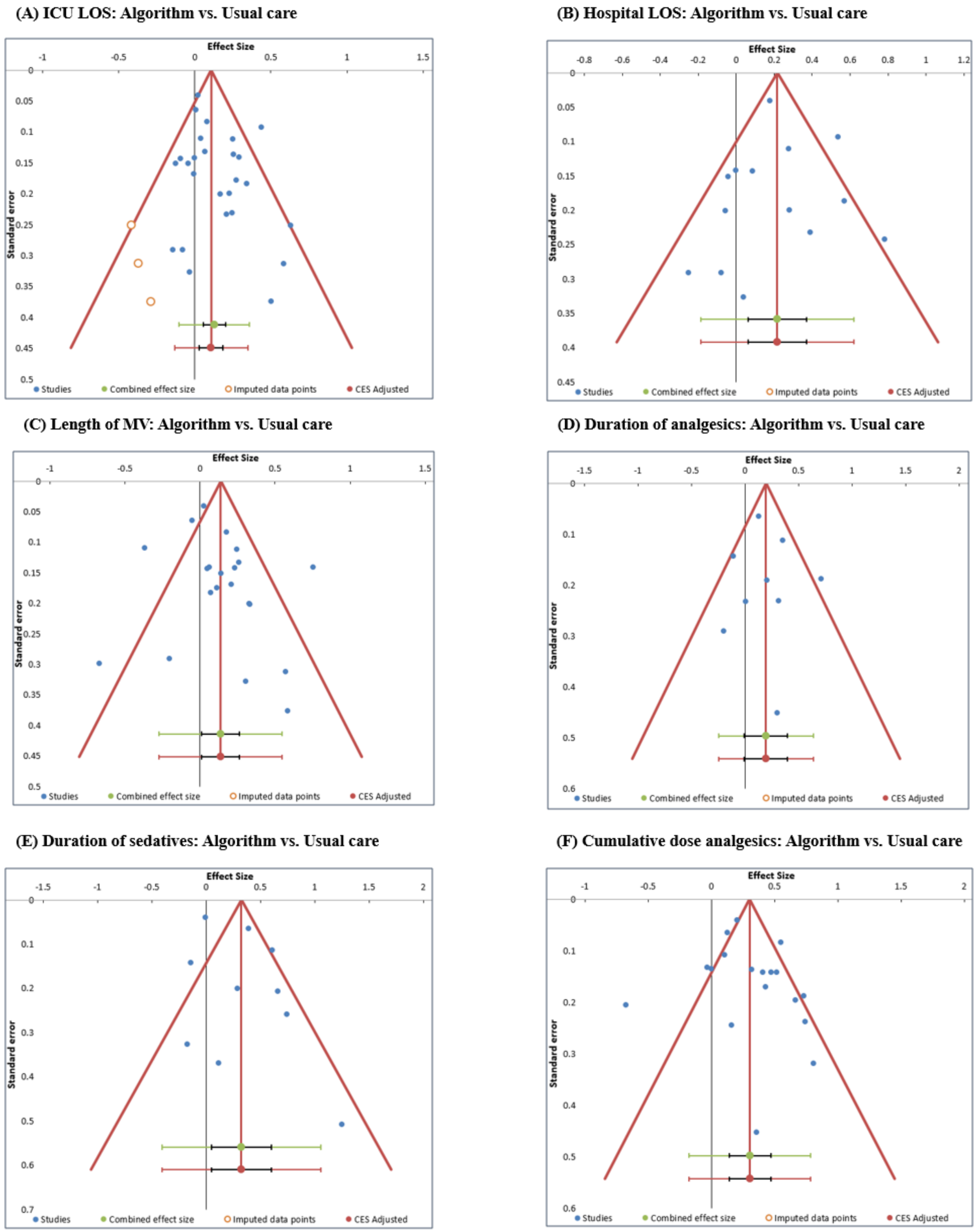

(G) Cumulative dose sedatives: Algorithm vs. Usual care

108 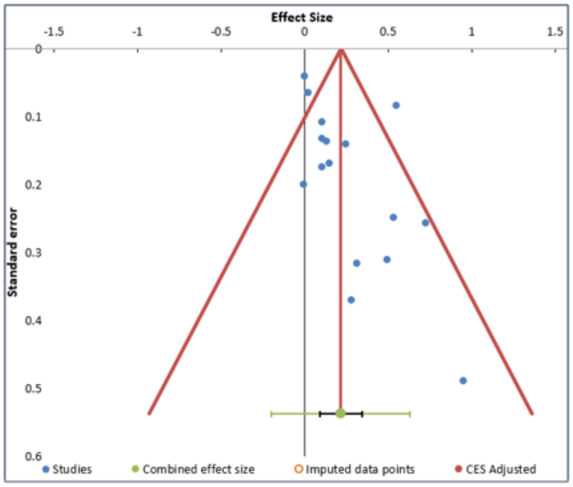

**Sensitivity analysis (leave one study at a time out)**

**Figure S10:** Sensitivity analysis – intensive care unit LOS

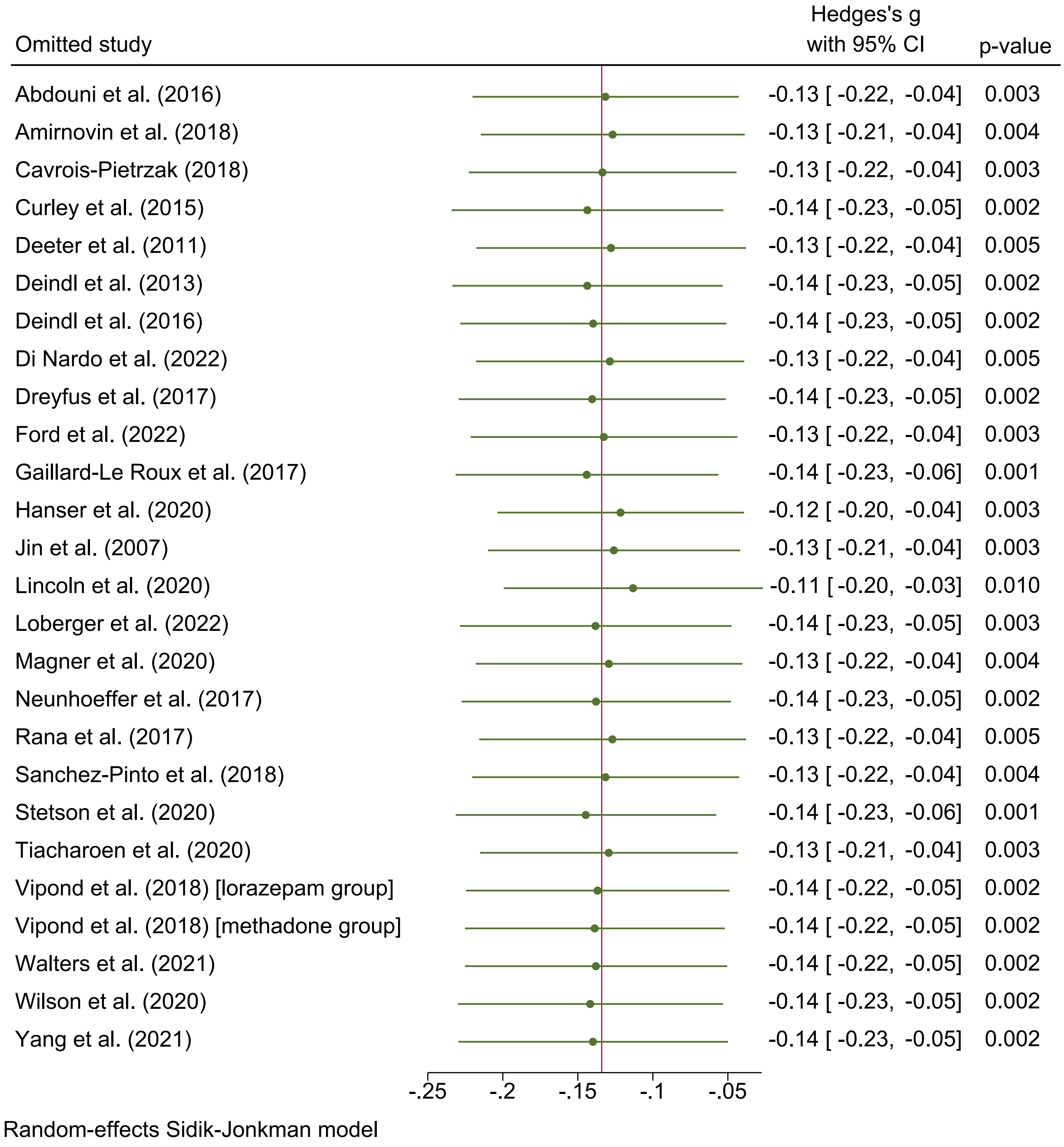

**Figure S11:** Sensitivity analysis – hospital LOS

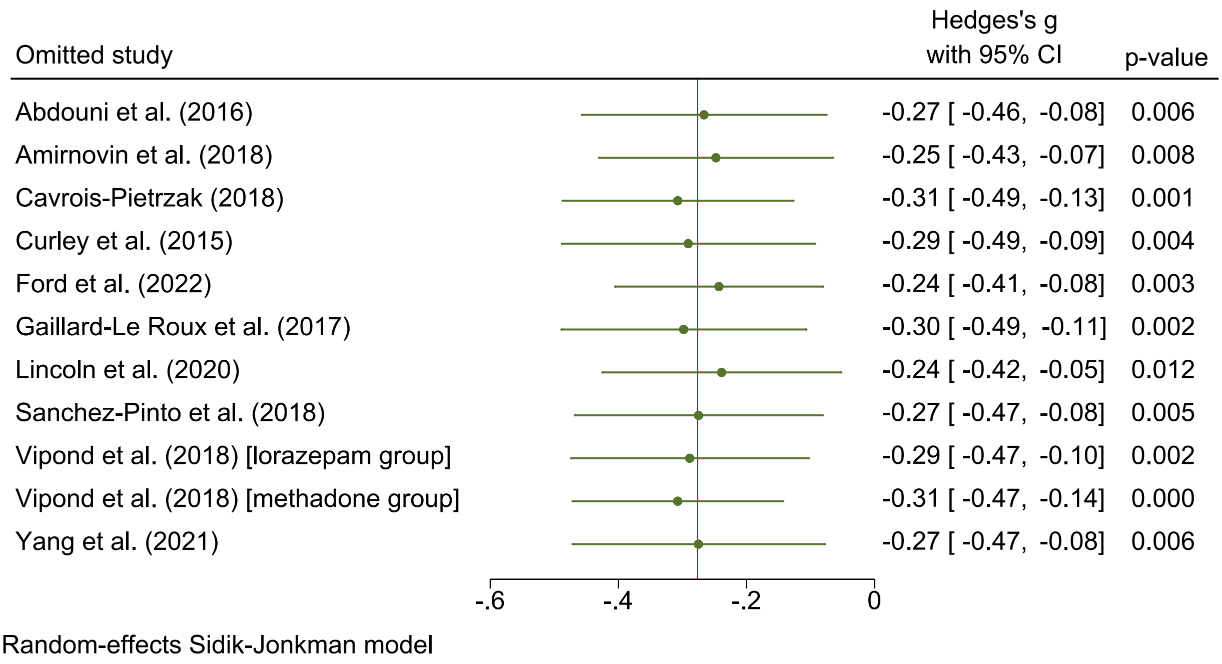

**Figure S12:** Sensitivity analysis – length of MV

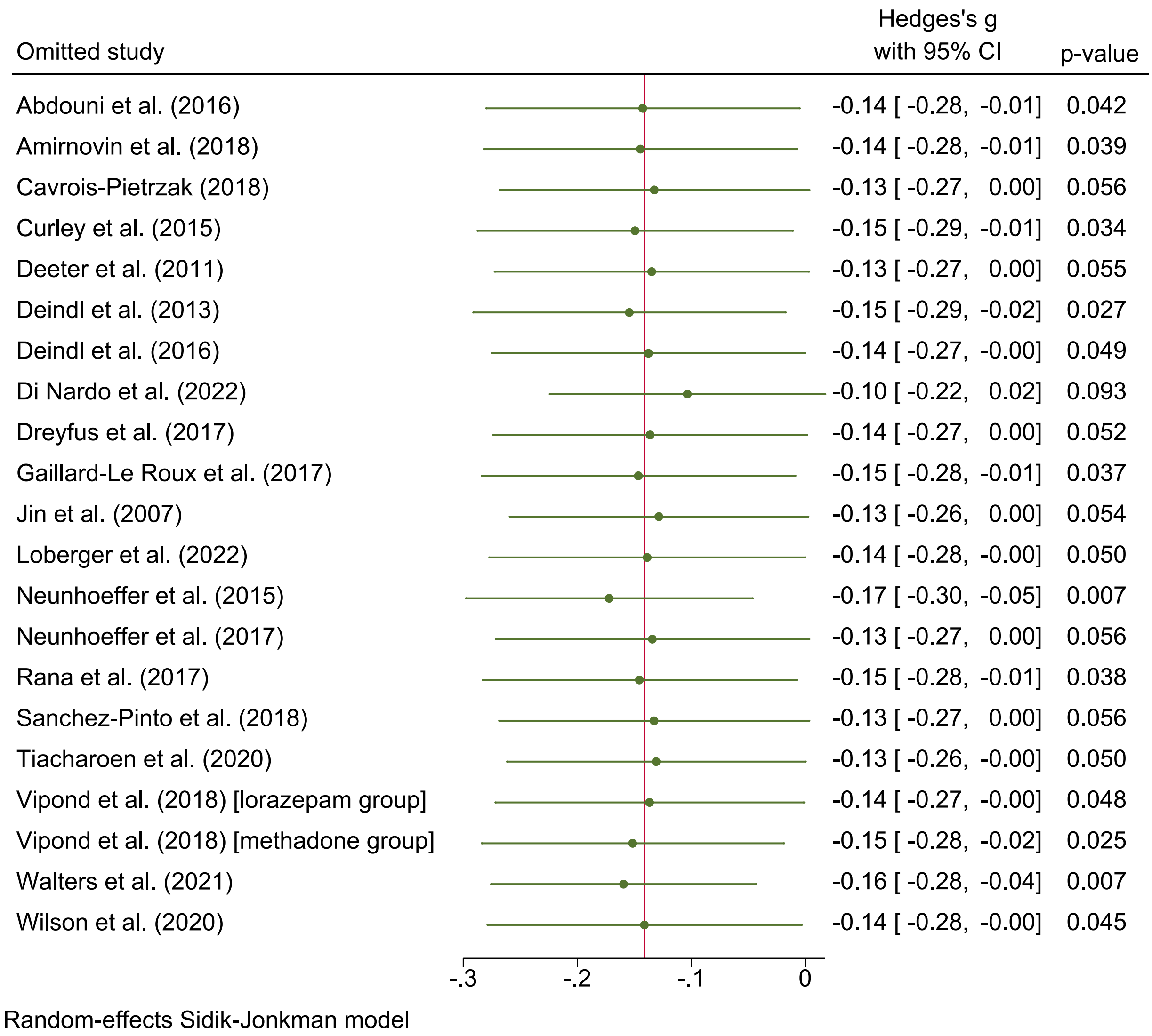

**Figure S13**: Sensitivity analysis – Duration of analgesic medications

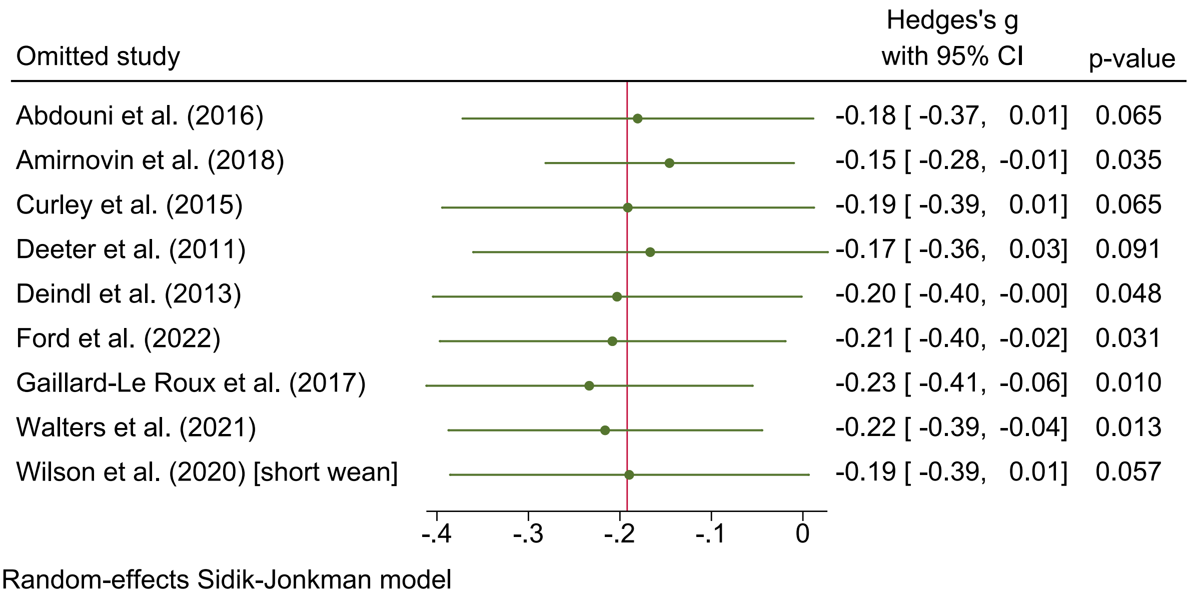


**Figure S14:** Sensitivity analysis – Duration of sedative medications

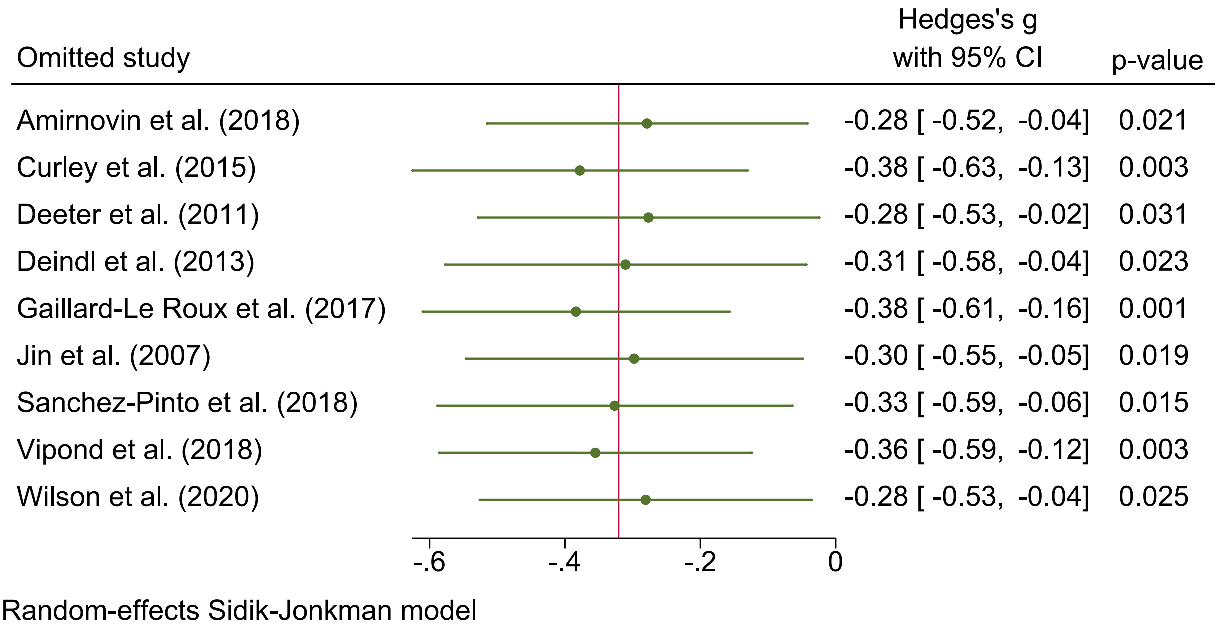

**Figure S15:** Sensitivity analysis – Cumulative dose of analgesic medications

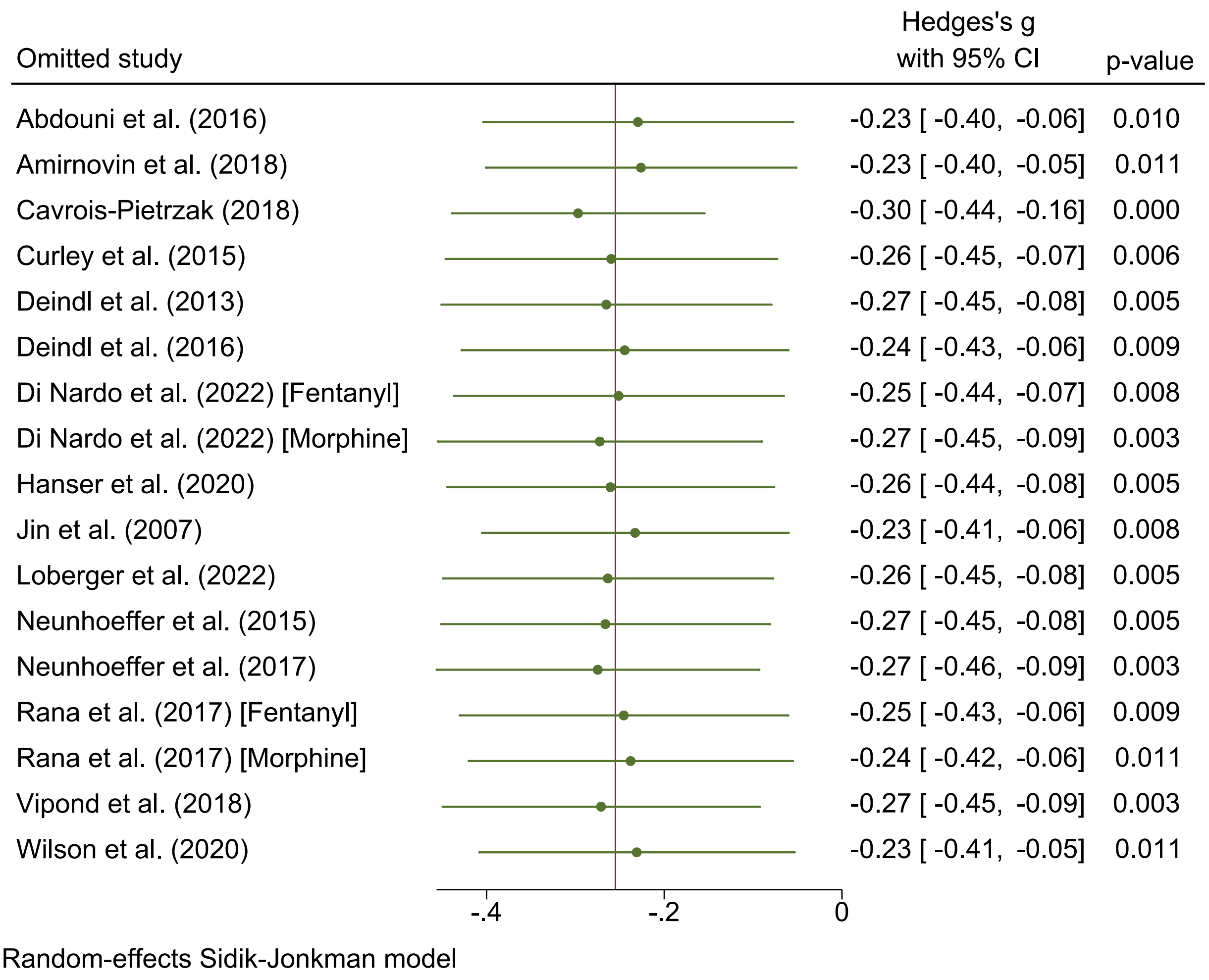

**Figure S16:** Sensitivity analysis – Cumulative dose of sedative medications

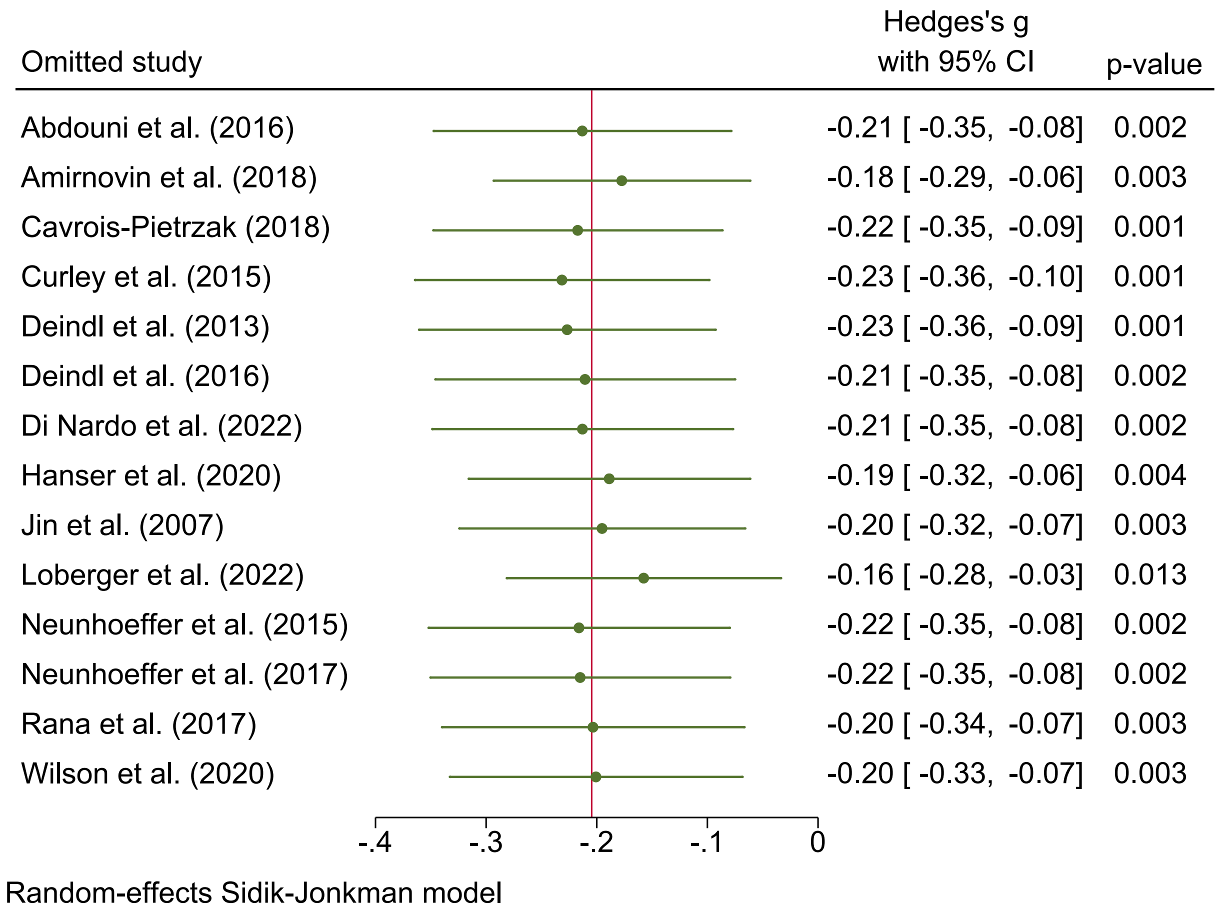

**Figure S17:** Sensitivity analysis – Incidents of iatrogenic withdrawal syndrome

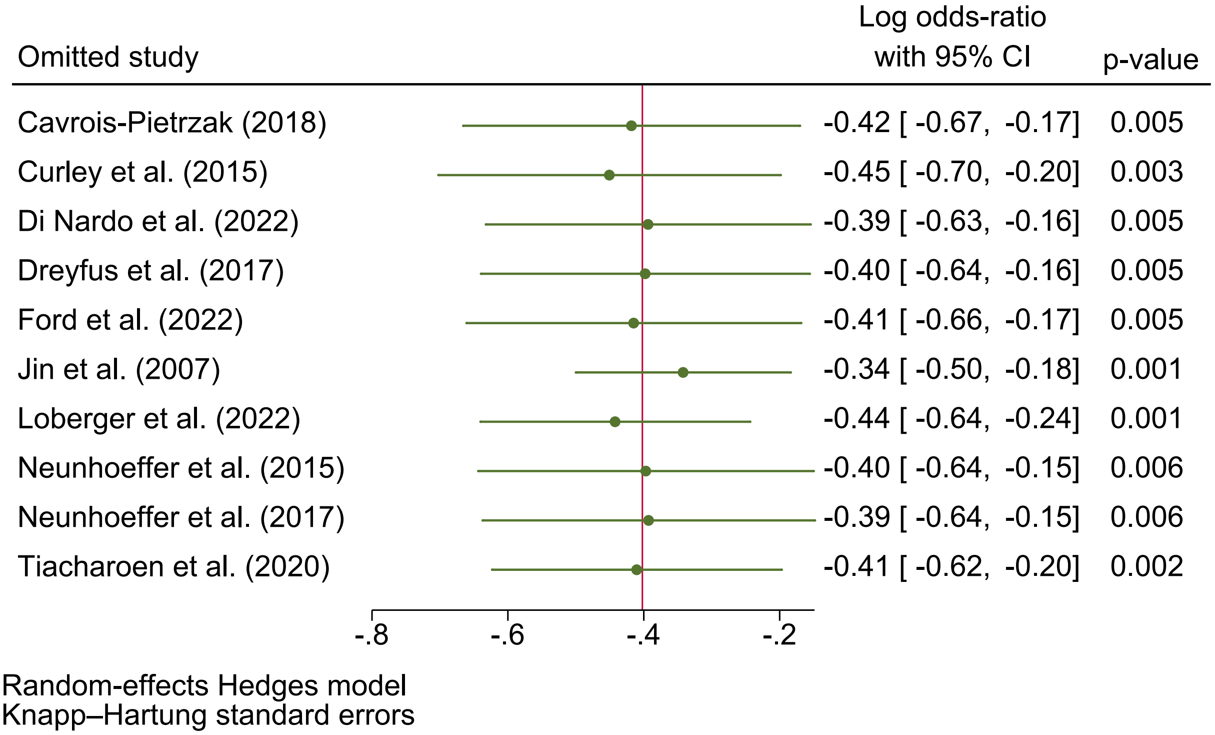


**Figure S18:** Sensitivity analysis – Incidents of under-sedation

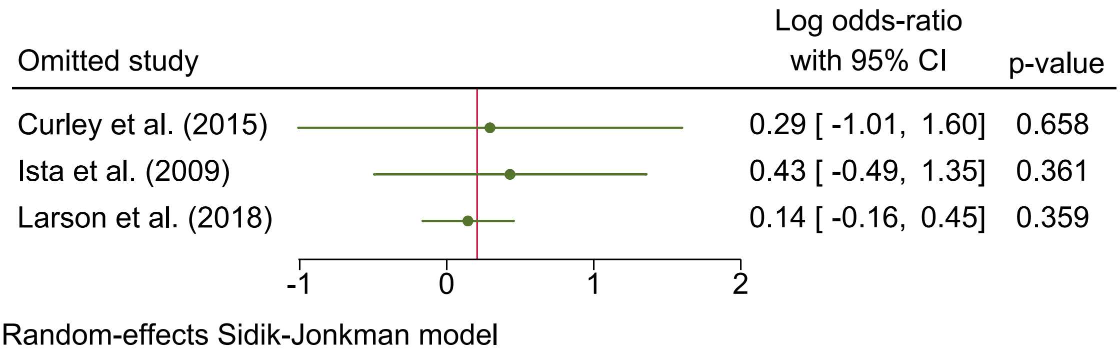

**Figure S19:** Sensitivity analysis – Duration of weaning

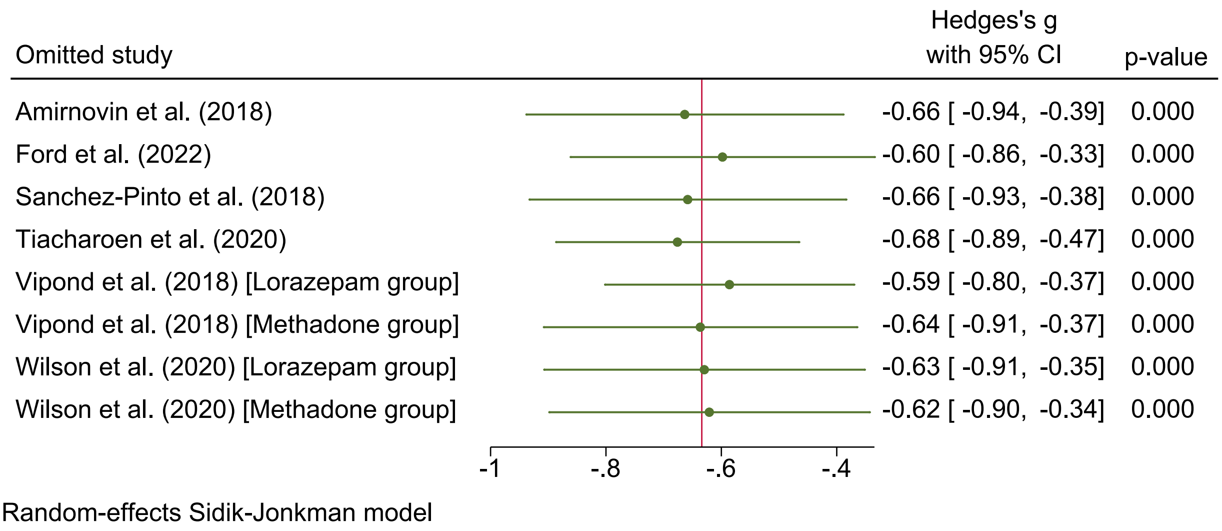


**Figure S20:** Sensitivity analysis – Duration of methadone

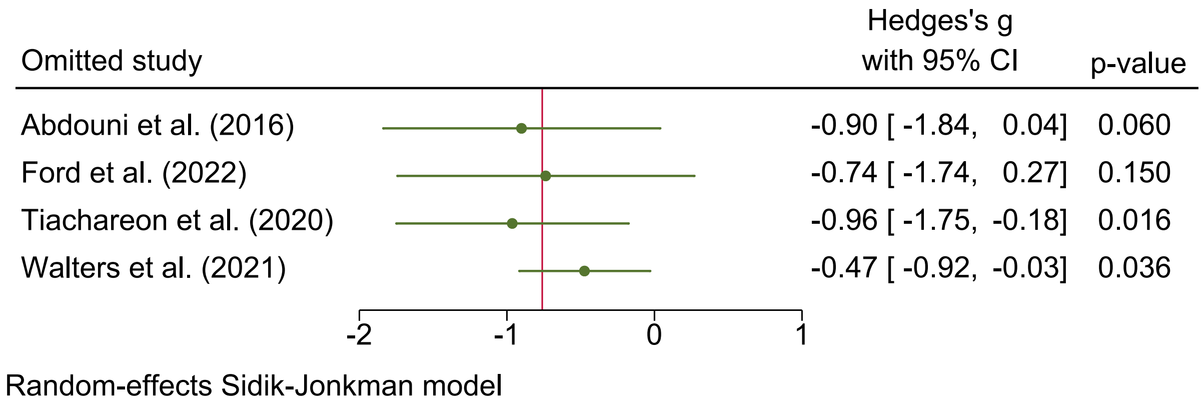

137 **Table S12:** PROFILE: Items and Quality Scores

| Three processes | Development | | | | | | | | | | | | | | | Content | | | | | Implementation | | | | |
|---|---|---|---|---|---|---|---|---|---|---|---|---|---|---|---|---|---|---|---|---|---|---|---|---|---|
| Five Domains | 1:Scope and purpose | | | 2: Stakeholder involvement | | | 3:Rigor of development | | | | | | | | | 4: Content, structure, documentation, roles | | | | | 5: Implementation | | | | |
| Item number | | 2 | 3 | 4 | 5 | 6 | 7 | 8 | 9 | 10 | 11 | 12 | 13 | 14 | 15 | 16 | 17 | 18 | 19 | 20 | 21 | 22 | 23 | 24 | |
| Item description / Author | Objectives | Interprofessional management | Population | Interprofessional development team | Staff views sought | Family/patient views sought | Method for searching evidence | Evaluation of evidence quality | CPG used | Strenght limitations | Development process | Finalization process | Health benefits, side-effects, risks | Link to evidence | Process for reviewing | Easy to follow | Manegement options | Documentation process | Roles and responsibilities | Targets timeframes | Need identified | Barriers and facilitators | Monitoring criteria | Multiple implementation strategies | Total |
| Abdouni [5] | 1 | 1 | 1 | 1 | 1 | | 1 | | | | | 1 | 1 | 1 | 1 | 1 | 1 | 1 | 1 | 1 | 1 | 1 | 1 | 1 | 19 |
| Amirnovin[6]/Sanchez-Pinto[7] | 1 | 1 | 1 | | | | | | | 1 | | | | 1 | | 1 | 1 | 1 | | 1 | | | | 1 | 10 |
| Cavrois-Pietrzak [13] | 1 | 1 | 1 | 1 | | | | | 1 | | | | | | | 1 | 1 | | 1 | 1 | 1 | 1 | 1 | 1 | 13 |
| Curley [26] | 1 | 1 | 1 | 1 | 1 | | 1 | | | 1 | 1 | 1 | 1 | 1 | | 1 | 1 | 1 | 1 | 1 | 1 | 1 | 1 | 1 | 20 |
| Deeter [14] | 1 | 1 | 1 | 1 | 1 | | 1 | | | | | 1 | 1 | | | 1 | 1 | | 1 | | | 1 | | 1 | 13 |
| Deindl [15, 16] | 1 | 1 | 1 | 1 | | | 1 | | | | | | | | | 1 | 1 | | | 1 | 1 | 1 | | 1 | 11 |
| Di Nardo [32] | 1 | 1 | 1 | 1 | 1 | | 1 | | | 1 | 1 | 1 | 1 | | 1 | | | 1 | 1 | 1 | 1 | 1 | 1 | 1 | 18 |
| Dreyfus [17] | 1 | 1 | 1 | | | | | | | | | | | | | 1 | 1 | | 1 | 1 | | | | 1 | 8 |
| Ford [8] | 1 | 1 | 1 | | 1 | | 1 | 1 | | 1 | 1 | 1 | 1 | 1 | 1 | 1 | 1 | 1 | 1 | 1 | 1 | | 1 | 1 | 20 |
| Gaillard-Le Roux [18] | 1 | 1 | 1 | 1 | | | 1 | | | | | | | | | 1 | 1 | | 1 | 1 | | | | 1 | 10 |
| Hazwani [4] | 1 | 1 | 1 | 1 | 1 | | 1 | | | | 1 | 1 | | | | | | 1 | 1 | 1 | 1 | 1 | 1 | 1 | 15 |
| Ista [19] | 1 | 1 | 1 | 1 | 1 | | 1 | | | | 1 | 1 | | 1 | 1 | 1 | 1 | 1 | 1 | 1 | 1 | 1 | | 1 | 18 |
| Jin [33] | 1 | 1 | 1 | | | | | | | | | | | | | 1 | 1 | | | 1 | | | | | 6 |
| Keogh [27] | 1 | 1 | 1 | | | | 1 | 1 | 1 | 1 | 1 | | 1 | 1 | 1 | 1 | 1 | 1 | | 1 | 1 | | | 1 | 17 |
| Kleiber [20] | 1 | | 1 | | | | | | | | | | | | | 1 | 1 | | | 1 | | | | 1 | 6 |
| Larson [21] | 1 | 1 | 1 | 1 | | | | | | | | | | | | 1 | 1 | | 1 | 1 | 1 | | | 1 | 10 |
| Lincoln [28] | 1 | 1 | 1 | 1 | 1 | | | | | | | | | | | | | 1 | 1 | 1 | | | 1 | 1 | 10 |
| Loberger [22] | 1 | 1 | 1 | 1 | 1 | | 1 | | | | | 1 | | | 1 | 1 | 1 | 1 | 1 | 1 | 1 | 1 | 1 | 1 | 17 |
| Magner [23] | 1 | 1 | 1 | 1 | 1 | 1 | 1 | 1 | 1 | 1 | 1 | 1 | 1 | 1 | 1 | 1 | 1 | 1 | 1 | 1 | 1 | 1 | 1 | 1 | 24 |
| Neunheoffer [29, 30]/Hanser[31] | 1 | 1 | 1 | 1 | | | 1 | | | | | | | | | 1 | 1 | | 1 | 1 | | | | 1 | 10 |
| Puthoff [24] | 1 | 1 | 1 | 1 | | | | | | 1 | 1 | | | 1 | | 1 | 1 | 1 | 1 | | 1 | 1 | 1 | 1 | 15 |
| Rana [3] | 1 | 1 | 1 | 1 | | | 1 | | | | | | | 1 | | 1 | 1 | | | | | | | | 8 |
| Stetson [34] | 1 | 1 | 1 | 1 | 1 | | | | | | | 1 | | | | 1 | 1 | 1 | | 1 | 1 | 1 | 1 | 1 | 14 |
| Tiachareon [9] | 1 | 1 | 1 | | | | 1 | | | | | | | | | 1 | 1 | | | 1 | | | | | 7 |
| Vipond [10] | 1 | 1 | 1 | | | | 1 | | | 1 | | | 1 | | 1 | 1 | 1 | | | 1 | 1 | | | | 11 |
| Walters [11] | 1 | 1 | 1 | | | | | | | | | | | | | 1 | 1 | 1 | 1 | 1 | | | | | 8 |
| Wilson [12] | 1 | | 1 | | | | 1 | | | 1 | | | 1 | 1 | | 1 | 1 | | | 1 | | | | 1 | 10 |
| Yang [25] | 1 | 1 | 1 | 1 | 1 | | 1 | | 1 | | | 1 | | | 1 | 1 | 1 | | 1 | 1 | 1 | | | 1 | 15 |

**Table S13:** EPOC strategies for algorithm implementation

| Author | Implementation strategies (EPOC, 2015 categories) | | | | | Education | | | | | | | | | | | | Total |
|---|---|---|---|---|---|---|---|---|---|---|---|---|---|---|---|---|---|---|
| | A&F | CIR | MP | CoP | CQI | Games | Materials | Meetings | Outreach visits | Inter-professional | Tests | LC | LOL | MS | R | rPROM | TI | |
| Abdouni [5] | | | | | ■ | | ■ | ■ | ■ | | | | ■ | | | | ■ | **6** |
| Amirnovin[6] | | | | | ■ | | ■ | ■ | ■ | | ■ | | | | | | | 5 |
| Cavrois-Pietrzak [13] | | | | | ■ | | ■ | ■ | | ■ | | | | | | | | 4 |
| Curley [26] | ■ | | | | | | ■ | ■ | ■ | ■ | ■ | ■ | ■ | | | | | **8** |
| Di Nardo [32] | | | | | | | ■ | | ■ | ■ | | | | | | | | **3** |
| Deeter [14] | | | | | ■ | | ■ | ■ | ■ | | | | | | | | | 4 |
| Deindl [15] | | | | | ■ | | ■ | ■ | ■ | | | | | | ■ | | | 5 |
| Dreyfus [17] | | | | | | | ■ | ■ | | | | | ■ | | | | | 3 |
| Ford [8] | | | ■ | | | | ■ | | | | | | ■ | | | | | **3** |
| Gaillard-Le Roux [18] | | | | | | | ■ | ■ | ■ | ■ | | | | | | | | 4 |
| Hawzani [4] | ■ | | | | ■ | | ■ | ■ | ■ | ■ | ■ | | ■ | | | | ■ | **9** |
| Ista [19] | | | | | | | ■ | ■ | | | ■ | | | | | | | **3** |
| Keogh [27] | | | | | | | ■ | ■ | ■ | | | | | | | | | **3** |
| Kleiber [20] | | | | | | | | ■ | ■ | | | | | | | | | 2 |
| Larson [21] | | | | | | | ■ | ■ | | | | | ■ | | | | | 3 |
| Lincoln [28] | | | | | ■ | | ■ | ■ | | | ■ | | ■ | | ■ | | | 6 |
| Loberger [22] | | | | | | | ■ | ■ | | | | | | | | | | 2 |
| Magner [23, 35] | | | | | ■ | | ■ | ■ | ■ | ■ | ■ | | ■ | ■ | ■ | | | **9** |
| Neunhoeffer [29] | | | | | | | ■ | ■ | ■ | | ■ | | ■ | | | | | 5 |
| Puthoff [24] | | | | | ■ | | ■ | ■ | | | | | | | | | | **3** |
| Rana [3] | | | | | | | ■ | ■ | | | | | | | | | | 2 |
| Sanchez-Pinto[7] | | | | | ■ | | ■ | ■ | | | | | | | | | | 3 |
| Stetson [34] | | | | | ■ | | ■ | ■ | | | | | | | ■ | | | 4 |
| Walters [11] | | | | | | | | ■ | | | | | | | | | | 1 |
| Wilson [12] | | | | | | | ■ | ■ | | | | | | | | | | 2 |
| Yang [25] | | | | | ■ | | ■ | ■ | ■ | | ■ | | ■ | | ■ | | | **7** |
| Total | 2 | 0 | 1 | 0 | 12 | 0 | 23 | 24 | 13 | 6 | 8 | 1 | 9 | 1 | 5 | 0 | 2 | |

A&F = audit feedback, CIR = clinical incident reporting, MP = monitoring the performance, CoP = community of practice, CQI = continuous quality improvement, LC = local consensus, LOL = local opinion leaders, MS = managerial supervision, R = reminders, rPROM = Routine patient-reported outcome measures, TI = tailored interventions

High quality algorithm

**Table S14:** Barriers and facilitators of algorithm implementation

| | Sub-category | Theme | Pre-implementation | Implementation | | Post-implementation | |
|---|---|---|---|---|---|---|---|
| | | | Barrier | Barrier | Facilitator | Barrier | Facilitator |
| Organizational | Culture | Leadership | | Lack of endorsement or leadership ownership by physicians and staff (5, 27) | | | |
| | | Organizational readiness and planning | Knowledge deficits (34) | Locum physicians/ temporary nursing staff (5)<br>Rotation of residents (4)<br>Lack of physician and staff awareness of the guideline (5) | | Poor knowledge of the protocol (13)<br>Lack of staff availability for training due to new electronic health record (34)* | Physician commitment to development (19)* |
| | Relationship | Inter-professional | Inconsistent communication between HCPs (34) | | | | |
| | Processes and systems | | Lack of familiarity with pain scores (34)<br>No standardized approach/ treatment protocol (15, 34)<br>Inconsistent review of scores during rounds (34)<br>Assessment tools unavailable (15, 34)<br>Not using pain scores to provide intervention (34)<br>Interpretation of pain scores is challenging (34) | Nurses not reporting sedation scores at rounds (4) | Daily PICU rounds/huddles (4) (5)*<br>Staff meetings with monitoring criteria presented (4) | | Night shift (15)*<br>Screening for delirium and introduction of the sedation protocol (32) |
| | Skill mix | Clarity about roles/ responsibilities | | Lack of role clarity (27) | | | |

| | Sub-category | Theme | Pre-implementation | Implementation | | Post-implementation | |
|---|---|---|---|---|---|---|---|
| | | | Barrier | Barrier | Facilitator | Barrier | Facilitator |
| | Involvement | Support from team members and management | Medical interventions initiated too late (15) | | Escalation process where physicians support nurses with decision-making (4) | Nurses hesitant to titrate medications on night shift (19)* | Physicians supervise use of protocol (19)* |
| | | Collaborative working | | | | | Interprofessional collaboration (15)* (32)<br>Teamwork (15)*<br>Team feedback (15)* |
| | | Shared vision | | | Increased awareness of sedation management (27) | | |
| **Professional** | Underlying philosophy | Relationship between professional and patient | Inability to report pain (34) | Patients with complex treatments/unstable difficult for nurses to make decisions (4)<br>Difficulty with applying protocol to some patients (27)<br>Lack of accommodation of increased drug tolerance with long-term patients (27) | | Nurses perceived children had fluctuating agitation, children were judged as having pain, child considered too alert, need to maintain ventilation (13)<br>Delirium screening is more difficult in patients below 2 years old and developmentally delayed (32) | |
| | Attitudes to change | Attitudes and beliefs | | | | | Positive attitudes of staff (15)* |
| | | Prior experience | Treatment not effective (15) | | | Fear of accidental extubation (13) | |
| | Competencies | | | Nurses lack of experience and confidence (4) | Staff education (4) (5)* | | |

| | | | Pre-implementation | Implementation | | Post-implementation | |
|---|---|---|---|---|---|---|---|
| | Sub-category | Theme | Barrier | Barrier | Facilitator | Barrier | Facilitator |
| **Intervention** | Nature and characteristics | Complexity | | Complexity of the algorithm (27)<br>Confusion with delineation and movement between phases of the algorithm (27) | | | Physicians found the protocol less confusing (10) |
| | | Evidence of benefit | | | | | Physicians stated protocol was more likely to meet patient needs (10)<br>Physicians found the protocol less time consuming (10) |
| | | Practicality and utility | | | Assists with decision-making (27)<br>Structured nature of the guideline (27) | | Data monitoring system for alerting nurses to assess patients comfort and pain each shift (19)* |

*Researcher reported barriers and facilitators, often in the discussion

**Table S15:** Fidelity to algorithms and subcomponents

| Author | % Adherence (range) | % dose of algorithm components (range) | | | | | | | | | | |
|---|---|---|---|---|---|---|---|---|---|---|---|---|
| | | Assessment | | | | | Medications | | | | | |
| | | Pain assessment | Sedation assessment | Withdrawal assessment | Pain and sedation assessment | Delirium screening | Medication rate | Loading dose | Bolus prescribed | Medication prescribed | Medication regime | Initial medication dose |
| Abdouni [5] | 67 | | | 76 | | | | | | | | |
| Amirnovin[6] | 95 (90 – 100) | | | | | | | | | | | |
| Cavrois-Pietrzak [13] | 46.3 | | | | | | | | | | | |
| Curley [26] | (71-100) | 89 | 85 | 65 | | | | | | | | |
| Deindl [15] | | | | | 81 | | | | | | | |
| Di Nardo [32] | 81.1 | | | | | 90.9 | | | | | | |
| Gaillard-Le Roux [18] | | | | | 95.9 | | | | | | | |
| Hazwani [4] | 75-86 | | | | | | | | | | | |
| Ista [19] | | | | | | | | | | | U-S: 45<br>O-S: 12 | |
| Keogh [27] | 36 | | | 75 | 95 | | | | | | | |
| Magner [23] | | | | | 70.3 | | 61 | 8.2 | 90.6 | (90.6-98) | | |
| Larson [21] | | | | | 94 | | | | | | | |
| Neunhoeffer [29] | (71-86.4) | | | | | | | | | | | |
| Neunhoeffer [30] | (74.8-88.9) | | | | | | | | | | | |
| Rana [3] | 95 | | | | | | | | | | 89 | |
| Sanchez-Pinto [7] | 90 (80-100) | | | | | | | | | | | |
| Vipond [10] | (68.4 – 77.3) | | | | | | | | | | | |
| Wilson [12] | | | | | | | | | | | | (68.4 – 77.3) |
| Yang [25] | 94.4 | | | | | | | | | | | |
| **Overall range** | **36 - 100** | | | **65 - 75** | **70.3 – 95.9** | | | | | | **12 - 89** | |
| U-S = under-sedation, O-S = over-sedation | | | | | | | | | | | | |

**Table S16:** Staff and parental satisfaction

| Author | Healthcare professional group | Satisfaction Pre-implementation (%) | Satisfaction Post-implementation (%) |
|---|---|---|---|
| Deindl et al. (15) | Physicians | 31 | 89 |
| | Nurses | 17 | 55 |
| Di Nardo et al. (32) | All staff | | 80.8 |
| Keogh et al. (27) § | Nurses | | 88.5 |
| Magner (35) | Nurses | 26.9 | 59.7 |
| Rana et al. (3) | Parents | 75 | 85 |
| Vipond et al. (10)* | Physicians | 20 | 91 |

§ satisfaction based on response to question: the guidelines improve overall sedation management
* grouped "satisfied" and "very satisfied" responses together

## References


1. Page MJ, Moher D, Bossuyt PM, Boutron I, Hoffmann TC, Mulrow CD, et al. PRISMA 2020 explanation and elaboration: updated guidance and exemplars for reporting systematic reviews. BMJ. 2021;372:n160.
2. Rethlefsen ML, Kirtley S, Waffenschmidt S, Ayala AP, Moher D, Page MJ, et al. PRISMA-S: an extension to the PRISMA Statement for Reporting Literature Searches in Systematic Reviews. Systematic Reviews. 2021;10(1):39.
3. Rana D, Bellflower B, Sahni J, Kaplan AJ, Owens NT, Arrindell EL, et al. Reduced narcotic and sedative utilization in a NICU after implementation of pain management guidelines. Journal of Perinatology. 2017;37(9):1038-42.
4. Hazwani T, Al Ahmady A, Kazzaz Y, Al Smari A, Al Enizy S, Alali H. Implementation of a sedation protocol: a quality improvement project to enhance sedation management in the paediatric intensive care unit. BMJ Open Qual. 2022;11(1):e001501.
5. Abdouni R, Reyburn-Orne T, Youssef TH, Haddad IY, Gerkin RD. Impact of a Standardized Treatment Guideline for Pediatric Iatrogenic Opioid Dependence: A Quality Improvement Initiative. The journal of pediatric pharmacology and therapeutics : JPPT : the official journal of PPAG. 2016;21(1):54-65.
6. Amirnovin R, Sanchez-Pinto LN, Okuhara C, Lieu P, Koh JY, Rodgers JW, et al. Implementation of a Risk-Stratified Opioid and Benzodiazepine Weaning Protocol in a Pediatric Cardiac ICU. Pediatr Crit Care Med. 2018;19(11):1024-32.
7. Sanchez-Pinto LN, Nelson LP, Lieu P, Koh JY, Rodgers JW, Larson KA, et al. Implementation of a risk-stratified opioid weaning protocol in a pediatric intensive care unit. Journal of Critical Care. 2018;43:214-9.
8. Ford J, Harthan A, McGarvey J, Mischler M. Implementation of an Opioid Weaning Protocol at a Tertiary Care Children’s Hospital. Hospital Pediatrics. 2022;12(11):945-53.
9. Tiacharoen D, Lertbunrian R, Veawpanich J, Suppalarkbunlue N, Anantasit N. Protocolized sedative weaning vs usual care in pediatric critically ill patients: a pilot randomized controlled trial. Indian Journal of Critical Care Medicine. 2020;24(6):451-8.
10. Vipond JM, Heiberger AL, Thompson PA, Huber JN. Shortened taper duration after implementation of a dtandardized protocol for iatrogenic benzodiazepine and opioid withdrawal in pediatric patients: Results of a cohort study. Pediatric quality safety. 2018;3(3).
11. Walters RA, Izquierdo M, Rodriguez JC, Stevens JS, Lavandosky G. Iatrogenic Opiate Withdrawal in Pediatric Patients: Implementation of a Standardized Methadone Weaning Protocol and Withdrawal Assessment Tool. Journal of Pharmacy Practice. 2021;34(3):417-22.
12. Wilson AK, Ragsdale CE, Sehgal I, Vaughn M, Padilla-Tolentino E, Barczyk AN, et al. Exposure-Based Methadone and Lorazepam Weaning Protocol Reduces Wean Length in Children. The journal of pediatric pharmacology and therapeutics : JPPT : the official journal of PPAG. 2021;26(1):42-9.
13. Cavrois-Pietrzak C. Evaluation d'un protocole de sédation-analgésie dans le service de réanimation pédiatrique de CHRU de Lille 2018.
14. Deeter KH, King MA, Ridling D, Irby GL, Lynn AM, Zimmerman JJ. Successful implementation of a pediatric sedation protocol for mechanically ventilated patients. Critical Care Medicine. 2011;39(4):683-8.
15. Deindl P, Unterasinger L, Kappler G, Werther T, Czaba C, Giordano V, et al. Successful implementation of a neonatal pain and sedation protocol at 2 NICUs. Pediatrics. 2013;132(1):e211-8.
16. Deindl P, Giordano V, Fuiko R, Waldhoer T, Unterasinger L, Berger A, et al. The implementation of systematic pain and sedation management has no impact on outcome in extremely preterm infants. Acta Paediatrica. 2016;105(7):798-805.
17. Dreyfus L, Bordet F, Touzet S, Denis A, Javouhey E. Implementation and evaluation of a paediatric nurse-driven sedation protocol in a PICU. Annals of Intensive Care. 2017;6.

18. Gaillard-Le Roux B, Liet JM, Bourgoin P, Legrand A, Roze JC, Joram N. Implementation of a Nurse-Driven Sedation Protocol in a PICU Decreases Daily Doses of Midazolam. Pediatr Crit Care Med. 2017;18(1):e9-e17.
19. Ista E, de Hoog M, Tibboel D, van Dijk M. Implementation of standard sedation management in paediatric intensive care: effective and feasible? J Clin Nurs. 2009;18(17):2511-20.
20. Kleiber N, de Wildt SN, Cortina G, Clifford M, van Rosmalen J, van Dijk M, et al. A comparative analysis of preemptive versus targeted sedation on cardiovascular stability after high-risk cardiac surgery in infants. Pediatric Critical Care Medicine. 2016;17(4):321-31.
21. Larson GE, McKeever S. Nurse titrated analgesia and sedation in intensive care increases the frequency of comfort assessment and reduces midazolam use in paediatric patients following cardiac surgery. Australian critical care : official journal of the Confederation of Australian Critical Care Nurses. 2018;31(1):31-6.
22. Loberger JM, Waddell KC, Prabhakaran P, Jones RM, Lawrence MV, Bittles LA, et al. Pediatric Ventilation Liberation: Bundled Extubation Readiness and Analgosedation Pathways Decrease Mechanical Ventilation Duration and Benzodiazepine Exposure. Respiratory Care. 2022;67(11):1385-95.
23. Magner C, Valkenburg AJ, Doherty D, van Dijk M, O'Hare B, Segurado R, et al. The impact of introducing nurse-led analgesia and sedation guidelines in ventilated infants following cardiac surgery. Intensive & critical care nursing. 2020;60:102879.
24. Puthoff TD, Shah H, Slaughter JL, Bapat R. Reduction of Analgesia Duration after Tracheostomy during Neonatal Intensive Care: A Quality Initiative. Pediatric quality & safety. 2018;3(5):e106-e.
25. Yang Y, Akhondi-Asl A, Geva A, Dwyer D, Stickney C, Kleinman ME, et al. Implementation of an Analgesia-Sedation Protocol Is Associated With Reduction in Midazolam Usage in the PICU. Pediatric Critical Care Medicine. 2021;22(10):e513-e23.
26. Curley MA, Wypij D, Watson RS, Grant MJ, Asaro LA, Cheifetz IM, et al. Protocolized sedation vs usual care in pediatric patients mechanically ventilated for acute respiratory failure: a randomized clinical trial. JAMA. 2015;313(4):379-89.
27. Keogh SJ, Long DA, Horn DV. Practice guidelines for sedation and analgesia management of critically ill children: a pilot study evaluating guideline impact and feasibility in the PICU. BMJ open. 2015;5(3):e006428.
28. Lincoln PA, Whelan K, Hartwell LP, Gauvreau K, Dodsen BL, LaRovere JM, et al. Nurse-Implemented Goal-Directed Strategy to Improve Pain and Sedation Management in a Pediatric Cardiac ICU. Pediatr Crit Care Med. 2020;21(12):1064-70.
29. Neunhoeffer F, Kumpf M, Renk H, Hanelt M, Berneck N, Bosk A, et al. Nurse-driven pediatric analgesia and sedation protocol reduces withdrawal symptoms in critically ill medical pediatric patients. Paediatric Anaesthesia. 2015;25(8):786-94.
30. Neunhoeffer F, Seitz G, Schmidt A, Renk H, Kumpf M, Fideler F, et al. Analgesia and Sedation Protocol for Mechanically Ventilated Postsurgical Children Reduces Benzodiazepines and Withdrawal Symptoms-But Not in All Patients. European Journal of Pediatric Surgery. 2017;27(3):255-62.
31. Hanser A, Neunhoeffer F, Hayer T, Hofbeck M, Schlensak C, Mustafi M, et al. A nurse-driven analgesia and sedation protocol reduces length of PICU stay and cumulative dose of benzodiazepines after corrective surgery for tetralogy of Fallot. J Spec Pediatr Nurs. 2020;25(3):e12291.
32. Di Nardo M, Boldrini F, Broccati F, Cancani F, Satta T, Stoppa F, et al. The LiberAction Project: Implementation of a Pediatric Liberation Bundle to Screen Delirium, Reduce Benzodiazepine Sedation, and Provide Early Mobilization in a Human Resource-Limited Pediatric Intensive Care Unit. Frontiers in Pediatrics. 2021;9.
33. Jin HS, Yum MS, Kim SL, Shin HY, Lee EH, Ha EJ, et al. The efficacy of the COMFORT scale in assessing optimal sedation in critically ill children requiring mechanical ventilation. J Korean Med Sci. 2007;22(4):693-7.

34. Stetson RC, Smith BN, Sanders NL, Misgen MA, Ferrie LJ, Schuning VS, et al. Reducing Opioid Exposure in a Level IV Neonatal Intensive Care Unit. Pediatric quality & safety. 2020;5(4):e312-e.
35. Magner C. The Impact of a Changed Approach to Analgesia and Sedation Management in the Paediatric Intensive Care Unit [PhD Thesis] 2014.
